# Supplementary figures and images for: Utilisation of semiconductor sequencing for detection of actionable fusions in solid tumours
Source: PLoS One. 2022 Aug 19;17(8):e0246778. doi: 10.1371/journal.pone.0246778 (PMC9390944; doi:10.1371/journal.pone.0246778)

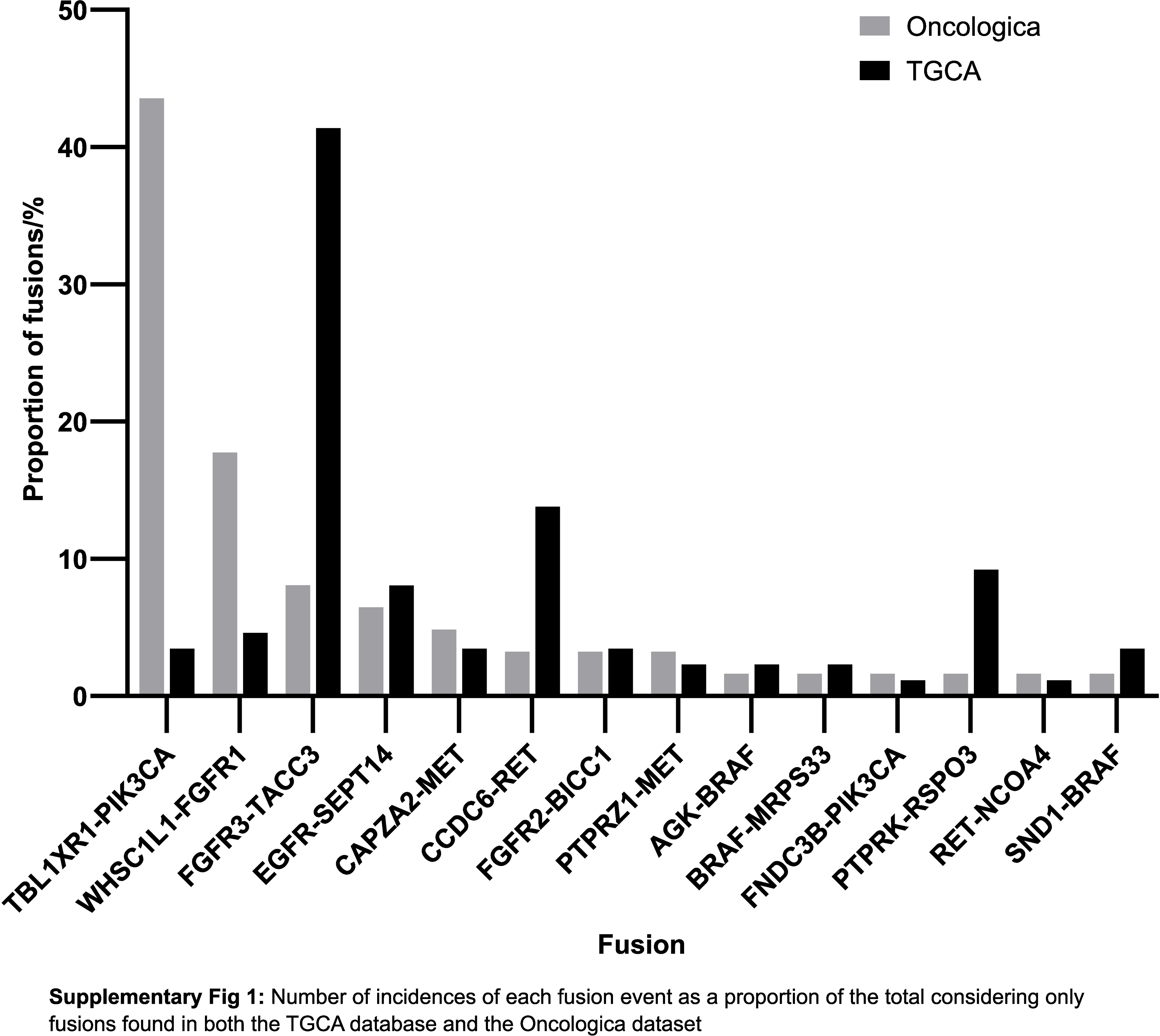

Supplement: S1 Fig — (TIF) [file pone.0246778.s001.tif]

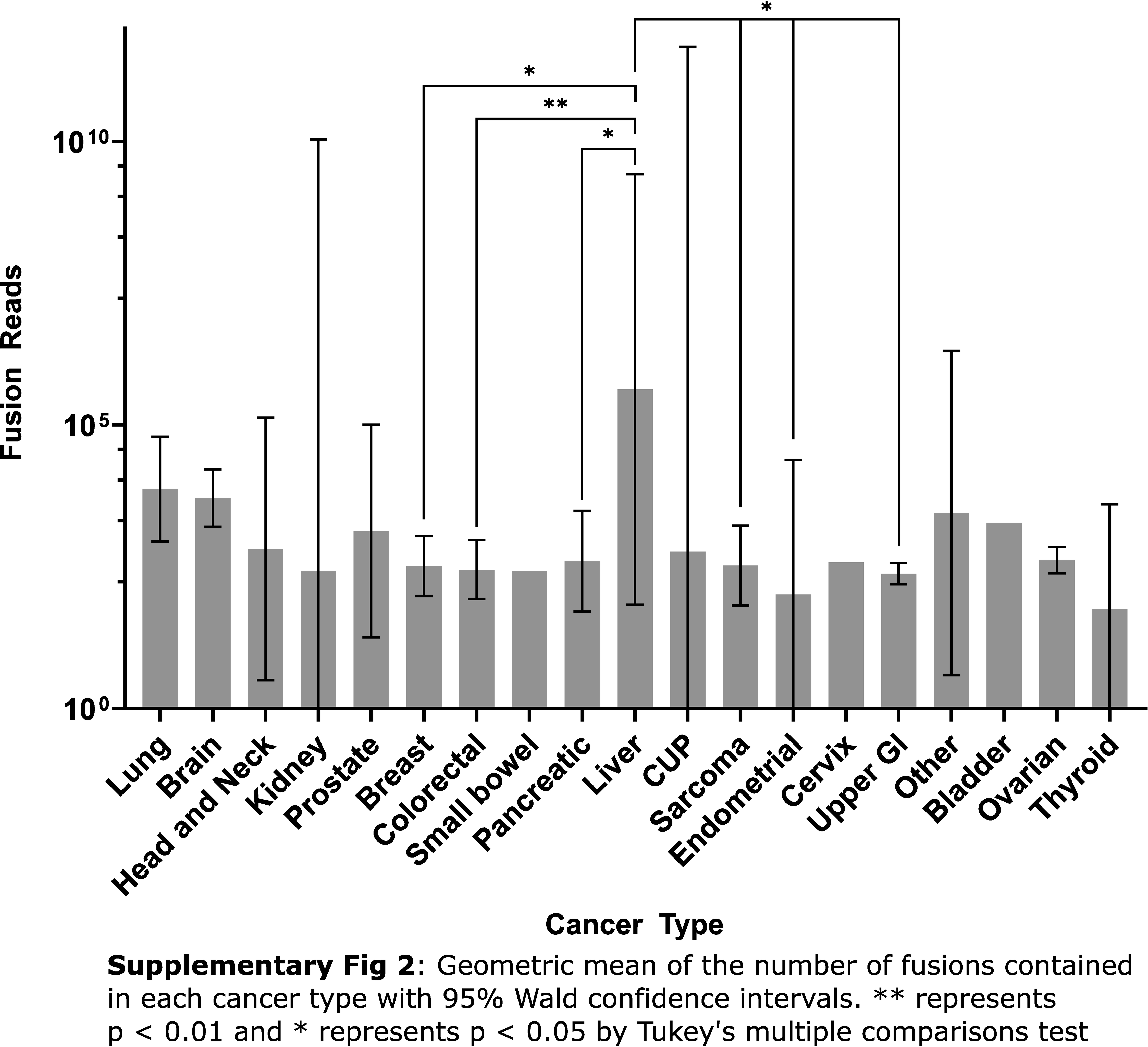

Supplement: S2 Fig — ** represents p < 0.01 and * < 0.05 by Tukey’s multipl comparisons test. (TIF) [file pone.0246778.s002.tif]
