# Supplementary material for: Utilisation of semiconductor sequencing for detection of actionable fusions in solid tumours
Source: PLoS One. 2022 Aug 19;17(8):e0246778. doi: 10.1371/journal.pone.0246778 (PMC9390944; doi:10.1371/journal.pone.0246778)
Supplement: S1 Table — (PDF) [file pone.0246778.s003.pdf]

Supplementary Table 1. Cancer type and histological classification of the study cohort.

Cancer Type	Primary / Metastatic lesion tested	N= 1112
**Breast**	*Primary carcinoma*<br>Invasive ductal (70)<br>Invasive lobular (5)<br>*Metastatic carcinoma* (101)	176
**Colorectal**	*Primary carcinoma*<br>Colorectal adenocarcinoma (109)<br>Appendiceal adenocarcinoma (4)<br>Appendiceal neuroendocrine carcinoma (1)<br>Anal squamous cell carcinoma (5)<br>*Metastatic carcinoma*<br>Colorectal adenocarcinoma (54)<br>Anal squamous cell carcinoma (1)<br>Rectal squamous cell carcinoma (1)<br>Appendiceal adenocarcinoma (1)<br>Appendiceal neuroendocrine carcinoma (1)	177
**Ovarian**	*Primary carcinoma*<br>Serous (38)<br>Mucinous (2)<br>Endometrioid (2)<br>Clear cell (3)<br>Undifferentiated (2)<br>Malignant sex cord stromal tumour (1)<br>Granulosa cell tumour (1)<br>*Metastatic carcinoma* (36)	85
**Glioma**	Astrocytoma<br>Oligodendroglioma<br>Glioblastoma	81
**Lung**	*Primary carcinoma*<br>NSCLC (58)<br>SCLC (14)<br>Mucoepidermoid (1)<br>*Metastatic carcinoma* (2)	75
**Upper GI**	*Primary carcinoma*<br>Oesophageal adenocarcinoma (23)<br>Oesophageal squamous cell carcinoma (10)<br>Oesophageal lymphoepithelial carcinoma (1)<br>Gastric adenocarcinoma (25)<br>Gastric neuroendocrine carcinoma (1)<br>Gastro-oesophageal junction adenocarcinoma (6)<br>*Metastatic carcinoma*<br>Oesophageal (4)<br>Gastric (4)<br>GOJ (1)	75
**Pancreatic**	*Primary carcinoma*<br>Adenocarcinoma (41)<br>Anaplastic carcinoma (1)<br>Adenosquamous carcinoma (1)<br>Neuroendocrine carcinoma (1)	71

	Metastatic carcinoma (27)	
**Sarcoma**	*Primary*<br>Leiomyosarcoma (11)<br>Liposarcoma (5)<br>Chordoma (3)<br>Ewing's sarcoma (3)<br>Pleomorphic sarcoma (3)<br>Rhabdomyosarcoma (3)<br>Angiosarcoma (2)<br>Chondrosarcoma (2)<br>Malignant peripheral nerve sheath tumour (2)<br>Other (11)<br>*Metastatic sarcoma* (12)	58
**Prostate**	*Primary carcinoma*<br>Adenocarcinoma (44)<br>*Metastatic carcinoma* (1)	45
**CUP**	*Metastatic carcinoma*<br>Poorly differentiated carcinoma (9)<br>Adenocarcinoma (22)<br>Squamous cell carcinoma (3)<br>Neuroendocrine carcinoma (4)	38
**Head & Neck**	*Primary carcinoma*<br>Squamous cell carcinoma (23)<br>Adenoid cystic carcinoma (3)<br>Acinic cell carcinoma (1)<br>Mucoepidermoid (3)<br>Salivary duct carcinoma (3)<br>Low grade parotid tumour (1)	34
**Liver**	*Primary carcinoma*<br>Cholangiocarcinoma (19)<br>Biliary tract adenocarcinoma (3)<br>Hepatocellular carcinoma (7)<br>Hepatoblastoma (1)<br>*Metastatic carcinoma*<br>Hepatocellular carcinoma (2)	32
**Bladder**	*Primary carcinoma*<br>Transitional cell carcinoma (17)<br>Adenocarcinoma (4)<br>Urethral adenocarcinoma (1)<br>*Metastatic carcinoma*<br>Transitional cell carcinoma (2)	24
**Other**	*Primary tumours*<br>Vulva squamous cell carcinoma (3)<br>Right buttock squamous cell carcinoma (1)<br>Mediastinal tumour (1)<br>NUT midline carcinoma (1)<br>Pecoma (1)<br>Merkel cell carcinoma (1)<br>Neurocytoma (1)<br>Pseudomyxoma peritonei (2)<br>Adrenal carcinoma (1)	19

	Peritoneal high grade serous carcinoma (1)<br>Testicular adenocarcinoma/germ cell tumour (1)<br>Yolk sac tumour (1)<br>Diffuse B-cell lymphoma (1)<br>Teratoma (1)<br>Neurocytoma (1)<br>Choroid plexus carcinoma (1)	
**Endometrial**	*Primary carcinoma*<br>Adenocarcinoma (8)<br>Serous carcinoma (4)<br>Carcinosarcoma (4)<br>*Metastatic carcinoma*<br>Adenocarcinoma (7)	23
**Cervix**	*Primary carcinoma*<br>Squamous cell carcinoma (12)<br>Adenocarcinoma (6)<br>Adenosquamous carcinoma (1)<br>*Metastatic carcinoma* (3)	22
**Mesothelioma**	*Primary*<br>Epitheliod (17)<br>Sarcomatoid (2)<br>Biphasic (2)	19
**Kidney**	*Primary carcinoma*<br>Transitional cell carcinoma (3)<br>Renal cell carcinoma (11)<br>*Metastatic carcinoma* (4)	18
**Melanoma**	*Primary*<br>Malignant melanoma (4)<br>Ocular spindle cell malignant melanoma (1)<br>*Metastatic malignant melanoma* (12)	17
**Thyroid**	*Primary carcinoma*<br>Papillary (2)<br>Follicular (3)<br>Anaplastic (3)<br>*Metastatic carcinoma (1)*	9
**Small Bowel**	*Primary carcinoma*<br>Adenocarcinoma (6)<br>*Metastatic carcinoma* (1)	7
**Gallbladder**	*Primary carcinoma*<br>Adenocarcinoma (7)	7
