# Supplementary material for: Utilisation of semiconductor sequencing for detection of actionable fusions in solid tumours
Source: PLoS One. 2022 Aug 19;17(8):e0246778. doi: 10.1371/journal.pone.0246778 (PMC9390944; doi:10.1371/journal.pone.0246778)
Supplement: S2 Table — (PDF) [file pone.0246778.s004.pdf]

Supplementary Table 2. Study cohort demographics by primary vs metastatic disease, age and gender.

Gender	Primary		Metastatic		Total	
	Number	Median Age	Number	Median Age	Number	Median Age
**Male**	343	61	108	59	451	61
**Female**	394	57	267	56	661	57
**Total**	737	59	375	57	1112	58
