# Supplementary material for: Utilisation of semiconductor sequencing for detection of actionable fusions in solid tumours
Source: PLoS One. 2022 Aug 19;17(8):e0246778. doi: 10.1371/journal.pone.0246778 (PMC9390944; doi:10.1371/journal.pone.0246778)
Supplement: S3 Table — (PDF) [file pone.0246778.s005.pdf]

Supplementary Table 3. Assay performance characteristics.

	**SNVs**	**Indel**	**CNV**	**Fusions**
**PPV***	>99%	>99%	>99%	>99%
**NPV****	>99%	>99%	>99%	>99%
**Accuracy**	>99%	>99%	>99%	>99%
**Sensitivity**	>99%	89%	>99%	>99%
**Specificity**	>99%	>99%	>99%	>99%

*Positive predictive value

**Negative predictive value
