# Supplementary material for: Utilisation of semiconductor sequencing for detection of actionable fusions in solid tumours
Source: PLoS One. 2022 Aug 19;17(8):e0246778. doi: 10.1371/journal.pone.0246778 (PMC9390944; doi:10.1371/journal.pone.0246778)
Supplement: S4 Table — (PDF) [file pone.0246778.s006.pdf]

Supplementary Table 4. Sequences of the 867 driver partner fusion events targeted by the Oncofocus Test.

>EGFR-SEPT14.E24S10

AGGAGGAGATAAGGAAGCTCGAGGAAGAGAAAAAACAACT

>NF1-ABCB5.N48A5

CAGCAAATTATCAGAACTGTACTCAGTCTCAAGAGAAGCTG

>NF1-ACACA.N1A42

CATTGTTATTGGCAATGACATCACATACCGAATTGGGTCCTTT

>EGFR-ACADM.E1A3

GCCGGAGCGAGCTCTTCGGGGAGCAGCGATGCGACCCTCCGGGACGGCCGGGGCAGCGCTCCTGGCGCTGCTGGCTGCGCTCTGCCCGGCGAGTCGGGCTCTGGAG
GAAAAGAAAGAGTTCACCGAACAGCAGAAAGAATTTCAAGCTACTGCTCGTAAATTTGCCAGAGAGGAAATCATCCCAGTGGCTGCA

>FGFR1-ADAM32.F17A14

CATCCTGAATGTGACATCGCTGAAAATTGTAATGG

>FGFR2-AFF3.F17A8.1

CTCCCAGAGACCAACGTTCAAGCAGTTGGTAGAAGACTTGGATCGAATTCTCACTCTCACAACCAATGAGGAGAGTAGATCTGGAGAAACCAACAGCTGTGTTGAAGA
AATAATCCGGGAGATGACCTGGCTTCCACCACTTTCTGCTATTCAAGCACCTGGCAAAGTGGAACCAACCAAATT

>FGFR2-AHCYL1.F17A2

CTCCCAGAGACCAACGTTCAAGCAGTTGGTAGAAGACTTGGATCGAATTCTCACTCTCACAACCAATGAGCAAATCCAGTTTGCTGATGACATGCAGGAGTTCACCAAAT

>ESR1-AKAP12.E5A4

TGAGCAAAAGAGACTCCGATAAAGAGATGGCTACTAAGTCAGCG

>ESR1-AKAP12.E6A4

GCTTACTGACCAACCTGGCAGACAGGGAGCTGGTTCACATGATCAACTGGGCGAAGAGGGTGCCAGTTGGACAGAGAGACTCTGAAGATGTGAGCAAAAGAGACTCC

>BCAM-AKT2.B13A5

CTCCTGCTCCTCGTCGTTGCTGTCTTCTACTGCGTGAGACGCAAAGGGGGCCCCTGCTGCCGCCAGCGGCGGGAGAAGGGGGCTCCGGAGGAGTGGATGCGGGCCAT

>ZNF226-AKT2.Z2A5

GACGACGTAGCAGCCATCTTTTCCCTGGCTTTGGTGATTCAGCCCTGACTTCTCAAAAAGCACTGCACAGAGGAGGAGGCAGCAGAACCCCATGGAGGAGTGGATGCG

>AR-AR.A4A8.V12

TTGCTCTCTAGCCTCAATGAACTGGGAGAGAGACAGCTTGTACACGTGGTCAAGTGGGCCAAGGCCTTGCCTGATTGCGAGAGAGCTGCATCAGTTCACTTTTGACCTG

>AR-AR.A6A8.V7es

ACCTGCTAATCAAGTCACACATGGTGAGCGTGGAC

>AR-AR.A3A4.V7

GTCCATCTTGTCGTCTTCGGAAATGTTATGAAGCAGGGATGACTCTGGGAGAAAAATTCCGGGTTGGCAATTGCAAGCATCTCAAAATGACCAGACCCTGAAGAA

>AR-AR.A3A4.V1

GTCCATCTTGTCGTCTTCGGAAATGTTATGAAGCAGGGATGACTCTGGGAGCAGCTGTTGTTGTTTCTGAAAGAATCTTGAGGGTGTTTGGAGTCTCAGAATGGCTTCCT

>AR-AR.A2A3.V3

ACTATGGAGCTCTCACATGTGGAAGCTGCAAGGTCTTCTTCAAAAGAGCCGCTGAAGGATTTTTCAGAATGAACAAATTAAAAGAATCATAATCAGACACTAACCCCAAG

>AR-AR.A3A3.V4

GTCCATCTTGTCGTCTTCGGAAATGTTATGAAGCAGGGATGACTCTGGGAGGATTTTTCAGAATGAACAAATTAAAAGAATCATAATCAGACACTAACCCCAAGCCATAC

>AR-AR.A1ins69A3

ACTTGTGTCAAAAGCGAAATGGGCCCCTGGATGGATAGCTACTCCGGACCTTACGGGGACATGCGAAATACCCGAAGAAAGAGACTCTGGAAACTCATTATCAGGTCT

>AR-AR.A3int4A3.V5

GTCCATCTTGTCGTCTTCGGAAATGTTATGAAGCAGGGATGACTCTGGGAGGACTAGAATTCCAAAGACCCTCAGGCTGGTGATGCAAGTGGGAAGTCTCATTTCTGAG

>AR-AR.A3A3int4.V6

GTCCATCTTGTCGTCTTCGGAAATGTTATGAAGCAGGGATGACTCTGGGAGGCAGGCAGCAGAGTGTCATAAAGAATTAACAACGTGGAACTCAGTTACTGGGATTTCT

>AR-AR.A3A3int4.V8

GTCCATCTTGTCGTCTTCGGAAATGTTATGAAGCAGGGATGACTCTGGGAGGGTTTTGACAACCTCTGTGAATTATCTAGTTGAGAGGATGGCTCAAGGAGCCTATTGC

>AR-AR.A3A3int4.V9

GTCCATCTTGTCGTCTTCGGAAATGTTATGAAGCAGGGATGACTCTGGGAGACAACTTACCTGAGCAAGCTGCTTTTTGGAGACATTTGCACATCTTTTGGGATCACGTT

>AR-AR.A3A5.V10

GTCCATCTTGTCGTCTTCGGAAATGTTATGAAGCAGGGATGACTCCCTCCAGCGGGACCAATAGTGTTTTCCTACCTCACAGGGATGTTGTGAGGACGGGCTGTAGAAG

>AR-AR.A6A8.V15

CTCTCTCAAGAGTTTGGATGGCTCCAAATCACCCCCCAGGAATTCCTGTGCATGAAAGCACTGCTACTCTTCAGCATTAGTCCTGTGAAGGAGCAGAGGGATAAAAAGA
GTAGAGGACATGATACATTGTACTTTACTAGTTCAAGACAGATGAATGTGGAAAGCA

>AR-AR.A6A9.V18

CTCTCTCAAGAGTTTGGATGGCTCCAAATCACCCCCCAGGAATTCCTGTGCATGAAAGCACTGCTACTCTTCAGCATTAGTTCCAGATCACACCTGATGCCATGTACTTGT

>AR-AR.A4A6.V5es

TTGCTCTCTAGCCTCAATGAACTGGGAGAGAGACAGCTTGTACACGTGGTCAAGTGGGCCAAGGCCTTGCCTGGTACCGCATGCACAAGTCCCGGATGTACAGCCAGT

>AR-AR.A8A9.V17

CCGGAAATGATGGCAGAGATCATCTCTGTGCAAGTGCCCAAGATCCTTTCTGGATCACACCTGATGCCATGTACTTGTGAGAGAGGATGCAGTTTTGTT

>AR-AR.A6A8.V13

CTCTCTCAAGAGTTTGGATGGCTCCAAATCACCCCCCAGGAATTCCTGTGCATGAAAGCACTGCTACTCTTCAGCATTAATCACACCTGATGCCATGTACTTGTGAGAGAG

>ESR1-ARMT1.E3A4

GCCCTACTACCTGGAGAACGAGCCCAGCGGCTACACGGTGCGCGAGGCCGGCCCGCCGGCATTCTACAGTCCACCAATCGATTACTTTGATGTATTTAAAGAATCAAAA

>NF1-ASIC2.N27A2

ACTCAGGCGAGGATGGCAAACCTCTGCTCACCACGGTCAAG

>NF1-ATAD5.N5A11

CATGCTTTGGACAGAAAAGTATCAACCTCAGACTGCCAGTG

>FGFR2-BICC1.F17B2

TTTCAAAAGATCATGGAGGAAACAAATACGCAGATTGCTTGGCCA

>FGFR2-BICC1.F17B3.1

CTCCCAGAGACCAACGTTCAAGCAGTTGGTAGAAGACTTGGATCGAATTCTCACTCTCACAACCAATGAGATCATGGAGGAAACAAATACGCAGATTGCTTGGCCA

>FGFR2-BICC1.F17B18

CACTTGGGAGGTGGAAGCGAATCTGATAACTGGAGAGACCGAAATG

>AKAP9-BRAF.A8B9.COSF1013.1

CTTAGAGCAACTCAACCAAGTGAAAATGATAAACTTCAGAAAGAACTCAATGTACTTAAATCAGAACAGGACTTGATTAGAGACCAAGGATTTCGTGGTGATGGAGGAT

>CLCN6-BRAF.C2B11.COSF1440

ACAGGAGGAGGAGGATGAGATTCTTCCAAGGAAAGACTATGAGAAAACACTTGGTAGACGGGACTCGAGTGATGATTGGGAGATTCCTGATGGGC

>FAM131B-BRAF.F1B10.COSF1191

GCCCCGAGTCGGTGAGGGCCCGGCTCTGCGGCCCCCGGAGCCATGGGCTGCATCGGCTCCCGGACTGTGGGATCAACCACAGGTTTGTCTGCTACCCCCCCTGCCTCAT

>FAM131B-BRAF.F2B9.COSF1189.1

GGCCTGAAGGATGTCGATCAAATCAACATGGACAGCACCAGCTCACTGCACGGGAGCAGCCTCCATCGGCCATCGACTGAGGACTTGATTAGAGACCAAGGATTTCGT

>FAM131B-BRAF.F3B9.COSF1193

GGCCTGAAGGATGTCGATCAAATCAACATGGACAGCACCAGCTCACTGCACGGGAGCAGCCTCCATCGGCCATCGACTGAGCAAACTCGAACTGATTTCTCCTGGGAC
GGCATCAACGACTTGATTAGAGACCAAGGATTTCGTGGTGATGGAGGATCAACCACAGGTTT

>FCHSD1-BRAF.F13B9.COSF403

AGGATGAGGTGGAGCAGGAGCGGCGGCTCAGTGAGGCTCGGCTGTCCCAGAGGGACCTCTCTCCAACCGACTTGATTAGAGACCAAGGATTTCGTGGTGATGGAGGA

>GNAI1-BRAF.G1B10.COSF1442

GTAAGATGATCGACCGCAACCTCCGTGAGGACGGCGAGAAGGCGGCGCGCGAGGTCAAGCTGCTGCTGCTCGGATCAACCACAGGTTTGTCTGCTACCCCCCCTGCCT

>KIAA1549-BRAF.K14B9.COSF483

TCATCACCACAGACAGCGATGGCACCTACAGGAGGCCCCCCGGCGTCCACAACTCAGCCTACATCGGATGCCCAGACTTGATTAGAGACCAAGGATTTCGTGGTGATGG

>KIAA1549-BRAF.K14B11.COSF1226

TCATCACCACAGACAGCGATGGCACCTACAGGAGGCCCCCCGGCGTCCACAACTCAGCCTACATCGGATGCCCAAAAACACTTGGTAGACGGGACTCGAGTGATGATTG

>KIAA1549-BRAF.K15B9.COSF481.1

GGTGTAGGCCCCGGAGTCCCACCCGGCCTGCCCGCAAACAGCACCCCTTCCCAGGAAGAGAGGCGAGCCACCCAGTGGGGGTCCTTCTACAGCCCAGCCCAGACGGC
CAACAATCCCTGCAGTGACTTGATTAGAGACCAAGGATTTCGTGGTGATGGAGGATCAACCACAGGTTT

>KIAA1549-BRAF.K15B11.COSF485.1

GGTGTAGGCCCCGGAGTCCCACCCGGCCTGCCCGCAAACAGCACCCCTTCCCAGGAAGAGAGGCGAGCCACCCAGTGGGGGTCCTTCTACAGCCCAGCCCAGACGGC
CAACAATCCCTGCAGTAAAACACTTGGTAGACGGGACTCGAGTGATGATTGGGAGATTCCTGATGGGC

>KIAA1549-BRAF.K17B10.COSF509

GCCCCATTTGCTGCCAGAGGGATCTACTCGGAGGAGATGCCGTCGGTGGCCCGGCCTCGGCCTGTCGGGGGTACCACAGGATCAACCACAGGTTTGTCTGCTACCCCC

>KIAA1549-BRAF.K18B9.COSF511

CAGAGGCAGCCAGTATGGGGGGCCAGGCTGGCCTTCGTACGGGGAGGACGAAGCGGGGCGAAGAGAGGCCGACTTGATTAGAGACCAAGGATTTCGTGGTGATGG

>MKRN1-BRAF.M4B11.COSF1444

CACGGAGATTCTTGTGACATGTGTGGGCTGCAGGTCCTGCATCCAATGGATGCTGCCCAGAGATCGCAGCATATCAAAAAAACACTTGGTAGACGGGACTCGAGTGAT

>RNF130-BRAF.R3B9.COSF1483

GCATGGCTCATATTCTACTTCATTCAGAAGATCAGGTACACAAATGCACGCGACAGGAACCAGGACTTGATTAGAGACCAAGGATTTCGTGGTGATGGAGGATCAACCA

>SLC45A3-BRAF.S1B8.COSF871

CTGGCTCCGGGTGACAGCCGCGCGCCTCGGCCAGGCCCCAAATTCTCACCAGTCCGTCTCCTTCAAAATCCATTCCAATTCCACAGCCCTTCCGA

>KIAA1549-BRAF.K12B9.COSF1474

GACTTGATTAGAGACCAAGGATTTCGTGGTGATGGAGGATCAACCACAGGTTT

>KIAA1549-BRAF.K15B10.COSF1283.1

GGTGTAGGCCCCGGAGTCCCACCCGGCCTGCCCGCAAACAGCACCCCTTCCCAGGAAGAGAGGCGAGCCACCCAGTGGGGGTCCTTCTACAGCCCAGCCCAGACGGC
CAACAATCCCTGCAGTAAATCTCCAGGACCTCAGCGAGAAAGGAAGTCATCTTCATCCTCAGAAGACAGGAATCGAATGAAAACACT

>AGK-BRAF.A2B8

GCTCTGCCTGCTGACCTGGGGAGGCCATTGGCTCTATGGAAAACACTGGCCCCAAATTCTCACCAGTCCGTCTCCTTCAAAATCCATTCCAATTCCACAGCCCTTCCGA

>KCTD7-BRAF.K4B8

GGGTGTGTGACAAGCACCTCGTGAACCACTACTACTGCAAGCGCCCCATCTATGAGTTCAAGATCACATGGTGGCCCCAAATTCTCACCAGTCCGTCTCCTTCAAAATCC

>MAD1L1-BRAF.M16B9

AGTCGGCTGGAGGAGGAAAAGAGGATGCTGGAGGCACAGCTGGAGCGGCGAGCTCTGCAGGACTTGATTAGAGACCAAGGATTTCGTGGTGATGGAGGATCAACCA

>NUDCD3-BRAF.N4B9

GGAAGCTCACCCACAAGATCAACACTGAGAGTTCTCTCTGGAGTCTCGAGCCCGGGAAGTGCGTTTTGGACTTGATTAGAGACCAAGGATTTCGTGGTGATGGAGGAT

>PLIN3-BRAF.P1B9

GACGGTTTCCAAGCTGGTTTTGAAGTCGCGGCAGCTGTTCCTGGGACGTCCGGTTGACCGCGGACTTGATTAGAGACCAAGGATTTCGTGGTGATGGAGGATCAACCA

>SOX6-BRAF.S5B9

CAACAGCAGCAACTTCTGCAACAGCAGCACAAAATTAATCTCCTGCAGCAACAGATCCAGGACTTGATTAGAGACCAAGGATTTCGTGGTGATGGAGGATCAACCACAG

>TRIM24-BRAF.T5B8

CACTAATCACCAAACTGATGGAAAAAACAAAATACATAAAATTCACAGGAAATCAGATCCAAAACAGGCCCCAAATTCTCACCAGTCCGTCTCCTTCAAAATCCATTCCA

>ZKSCAN5-BRAF.Z2B9

GAACATCACCCTGAAAGTGGAGAAGAGGCGGTGGCCGTGATAGAAAATATACAGCGAGAACTTGAGGAACGCAGACAGCAGGACTTGATTAGAGACCAAGGATTTCG

>CDC27-BRAF.C16B9.1

CAAGAACCCTCTATGCAAATTTCACAGAGCCTCAGTTTTATTTGCAAATGAAAAATATAAGGACTTGATTAGAGACCAAGGATTTCGTGGTGATGGAGGATCAACCACA

>KIAA1549-BRAF.K16B10

AATCCCTGCAGTAGATACGAAGACTATGGAATGACTCCCCCGACGGGTCCATTGCCAAGGATCAACCACAGGTTTGTCTGCTACCCCCCCTGCCTCATTACCTGGCTCAC

>PAPSS1-BRAF.P5B9.1

GATGTAAATGACTGTGTCCAGCAAGTTGTGGAACTTCTACAGGAACGGGACTTGATTAGAGACCAAGGATTTCGTGGTGATGGAGGATCAACCACAGGTTT

>SND1-BRAF.S16B9.1

GCTTGGTGCAGGAAGGAGAGCCCTTCAGCGAGGAAGCTACACTTTTCACCAAGGAACTGGTGCTGCAGCGAGAGGACTTGATTAGAGACCAAGGATTTCGTGGTGAT

>TAX1BP1-BRAF.T8B11.1

GCTGCAGTTATGTTTGGCTGAAAAGGAAAATCTGCAAAGAACTTTCCTGCTTACAACCTCAAGTAGACGGGACTCGAGTGATGATTGGGAGATTCCTGATGGGC

>TRIM24-BRAF.T9B9.1

CAACACAGATCAGCCTAGCTCAATTACGGCTCCAGCATATGCAGCAACAGGACTTGATTAGAGACCAAGGATTTCGTGGTGATGGAGGATCAACCACAGGTTT

>AGTRAP-BRAF.A5B8.COSF828.1

TCTCCTTCAAAATCCATTCCAATTCCACAGCCCTTCCGA

>SND1-BRAF.S10B11

AACTCAGGCGATTACAAGACGATTCACCTGTCCAGCATCCGACCACCGAGGCTGGAGGGGGAGAACACCCAGAAAACACTTGGTAGACGGGACTCGAGTGATGATTG

>ATG7-BRAF.A18B9

CTAGCCAAGGTGTTTAATTCTTCACATTCCTTCTTAGAAGACTTGACTGGTCTTACATTGCTGCATCAAGAAACCCAAGCTGCTGAGGACTTGATTAGAGACCAAGGATTT

>GATM-BRAF.G2B11

CTTACAACGAATGGGACCCCTTAGAGGAAGTGATAGTGGGCAGAGCAGAAAACGCCTGTGTTCCACCGTTCACCATCGAGGTGAAGAAAACACTTGGTAGACGGGACT

>SND1-BRAF.S14B11

GCTATTAAGAATGGCAAAGGATTGCATAGCAAGAAGGAAGTGCCTATCCACCGTGTTGCAGATATATCTGGGAAAACACTTGGTAGACGGGACTCGAGTGATGATTGG

>CEP89-BRAF.C16B9

GGATTTCGTGGTGATGGAGGATCAACCACAGGTTT

>TMPRSS2-BRAF.T3B11

CTGACGCAGGCTTCCAACCCCGTCGTCTGCACGCAGCCCAAATCCCCATCCGGGACAGTGTGCACCTCAAAAAACACTTGGTAGACGGGACTCGAGTGATGATTGGGAG

>MACF1-BRAF.M60B9

GATTTCGTGGTGATGGAGGATCAACCACAGGTTT

>SND1-BRAF.S9B9

AGGCTGAGAATATGGAGAGACTATGTGGCTCCCACAGCTAATTTGGACCAAAAGGACAAGCAGTTTGTTGCCAAGGACTTGATTAGAGACCAAGGATTTCGTGGTGAT

>KDM7A-BRAF.K11B11

TGTATCTGAACATGCCTTTGAAATTCCAGACAATGTTAGACCTGGACACCTTATTAAAGAACTTTCTAAAGTAATTCGAGCAATAGAGAAAACACTTGGTAGACGGGACT

>C7orf73-BRAF.C2B9

CCTCCCCACCGACATCATGCTCCAGTTCCTGCTTGGATTTACACTGGGCAACGTGGTTGGAATGTATCTGGCTCAGAACTATGATGACTTGATTAGAGACCAAGGATTTC

>SND1-BRAF.S14B9

GCTATTAAGAATGGCAAAGGATTGCATAGCAAGAAGGAAGTGCCTATCCACCGTGTTGCAGATATATCTGGGGACTTGATTAGAGACCAAGGATTTCGTGGTGATGGA

>ZSCAN30-BRAF.Z3B10

AGAGAATGGAGAGGAAGCTGTGACTATGCTGGAGGAGCTGGAAAAAGAACTGGAGGAGCCAAGGCAACAGGATCAACCACAGGTTTGTCTGCTACCCCCCCTGCCTC

>AP3B1-BRAF.A22B9

AGTTGCACTTCCCACACCAGCTCTTTCTCCAAGTTTGATGGCTGATCTTGAAGGTTTACACTTGTCAACTTCCTCTTCAGTCATCAGTGACTTGATTAGAGACCAAGGATTT

>HERPUD1-BRAF.H4B7

ACAGGAAGAGGCGTCCTTAGCAGAGACTGCCCTAACATCT

>LSM14A-BRAF.L9B9

TCACTGCCCCTCGAGGATTTCGCGGTGGATTCAGAGGAGGTCGTGGGGGCCGGGAGTTTGCGGATTTTGAATATAGGGACTTGATTAGAGACCAAGGATTTCGTGGTG

>BCL2L11-BRAF.B3B10

GTGACAAATCAACACAAACCCCAAGTCCTCCTTGCCAGGCCTTCAACCACTATCTCAGTGCAATGGGATCAACCACAGGTTTGTCTGCTACCCCCCCTGCCTCATTACCTG

>FAM114A2-BRAF.F9B11

GGGATGAAGATTTTACCAAGGACATAACAGAGCTGTTTTCCCAGCTGCACGTTTCCTCCAAACCAGAGAAACTTGCCAGGAAAACACTTGGTAGACGGGACTCGAGTGA

>SND1-BRAF.S10B9

AACTCAGGCGATTACAAGACGATTCACCTGTCCAGCATCCGACCACCGAGGCTGGAGGGGGAGAACACCCAGGACTTGATTAGAGACCAAGGATTTCGTGGTGATGGA

>ZC3HAV1-BRAF.Z7B11

ATTCTGTCTTCACCACCAAATGGATTTGGTATTGGAAGAATGAATCTGGCACATGGATTCAGTATGGAGAAGAGAAAACACTTGGTAGACGGGACTCGAGTGATGATTG

>CCNY-BRAF.C1B10

TGCAACCTGCAGCACATCAGCGACCGGGAGAACATAGACGGATCAACCACAGGTTTGTCTGCTACCCCCCCTGCCTCATTACCTGGCTCACTAACTAACGTG

>MKRN1-BRAF.M4B9

CACGGAGATTCTTGTGACATGTGTGGGCTGCAGGTCCTGCATCCAATGGATGCTGCCCAGAGATCGCAGCATATCAAAGACTTGATTAGAGACCAAGGATTTCGTGGTG

>SND1-BRAF.S18B10

TGTGACTGAGATCACTGATGACCTGCACTTCTACGTGCAGGATGTGGAGACCGGATCAACCACAGGTTTGTCTGCTACCCCCCCTGCCTCATTACCTGGCTCACTAACTA

>TRIM4-BRAF.T6B10

TTGAAGCTGTAAAGGTGAAGACAGTGTGCCAGATACCATTGATGAAGGAAATGCTAAAGCGATTCCAAGGATCAACCACAGGTTTGTCTGCTACCCCCCCTGCCTCATT

>CUL1-BRAF.C7B9

TCTTGCAGCAGAACCCAGTTACTGAATATATGAAAAAGGACTTGATTAGAGACCAAGGATTTCGTGGTGATGGAGGATCAACCACAGGTTT

>CUX1-BRAF.C10B9

AGACCTTAAGGGAACAGCTCTCATCGGCCAATCACTCCCTCCAGCTGGCCTCACAGATCCAGAAGGCACCAGACGTGGACTTGATTAGAGACCAAGGATTTCGTGGTGA

>GTF2I-BRAF.G4B10

AAATCGGATGAGTGTAGATGCTGTAGAAATTGAAACACTCAGAAAAACAGTTGAGGACTATTTCTGCTTTTGCTATGGATCAACCACAGGTTTGTCTGCTACCCCCCCTG

>SND1-BRAF.S11B11

CGTCCCCTGTATGACATTCCTTACATGTTTGAGGCCCGGGAATTTCTTCGAAAAAAGCTTATTGGGAAGAAGAAAACACTTGGTAGACGGGACTCGAGTGATGATTGGG

>TRIM24-BRAF.T3B10

CGTGTATCAGAGCTCATCAGAGGGTAAAGTTCACAAAAGACCACACTGTCAGACAGAAAGAGGAAGTATCTCCAGGATCAACCACAGGTTTGTCTGCTACCCCCCCTGC

>GHR-BRAF.G1B10

GAACCCGCGCTCTCTGATCAGAGGCGAAGCTCGGAGGATCAACCACAGGTTTGTCTGCTACCCCCCCTGCCTCATTACCTGGCTCACTAACTAACGTG

>SLC12A7-BRAF.S17B11

CAAGGGCCTGACCATCGTGGGCTCGGTGCTGGAGGGGACGTACCTGGACAAGCACATGGAGGCTCAGCGGGCCGAGGAGAAAACACTTGGTAGACGGGACTCGAGT

>EPS15-BRAF.E22B10

CCCCTTTGCTTCTGTTTTTGGGAATGAATCATTTGGAGGTGGATTTGCTGACTTCAGCACATTGTCAAAGGATCAACCACAGGTTTGTCTGCTACCCCCCCTGCCTCATTAC

>KCTD7-BRAF.K3B8

GGAGAACATGCAGCCACTGAAGGGCGAGAAGGTGCGCCAAGCGTTTCTGGGACTCATGCCCTATTACAAAGGCCCCAAATTCTCACCAGTCCGTCTCCTTCAAAATCCA

>RAD18-BRAF.R7B10

CAACAGCTCATTAAAAGGCACCAAGAATTTGTACACATGTACAATGCCCAATGCGATGCTTTGCATCCTAAATCAGGATCAACCACAGGTTTGTCTGCTACCCCCCCTGCC

>CCDC91-BRAF.C11B9

TTTCGTGGTGATGGAGGATCAACCACAGGTTT

>ARMC10-BRAF.A4B11

GACTCGAGTGATGATTGGGAGATTCCTGATGGGC

>AKAP9-BRAF.A28B9

AGGATTTCGTGGTGATGGAGGATCAACCACAGGTTT

>KIAA1549-BRAF.K13B9

GGCACAGATGCAGATCGACAAGATCCTGGACCCCACGGCCAGCGTGCCCTCCGTGTTCATAGAGCCCAGGAAGAGGACTTGATTAGAGACCAAGGATTTCGTGGTGAT

>AKAP9-BRAF.A22B9

CTCTCGACTACAAGCAGCAGTTGAAAAACTCCTAGAAGCCATAAGTGAAACTAGCAGTCAGGACTTGATTAGAGACCAAGGATTTCGTGGTGATGGAGGATCAACCAC

>KLHL7-BRAF.K5B9

TGTCAAGCGAGTAACACATCTTCTCAACCAGGACACTCTGACTGTGAGAGCAGAGGATCAGGACTTGATTAGAGACCAAGGATTTCGTGGTGATGGAGGATCAACCAC

>AGAP3-BRAF.A9B9

GGATTTCGTGGTGATGGAGGATCAACCACAGGTTT

>NUB1-BRAF.N3B9

TTCGTGGTGATGGAGGATCAACCACAGGTTT

>TRIM24-BRAF.T10B9

GATTTCGTGGTGATGGAGGATCAACCACAGGTTT

>AKAP9-BRAF.A7B11

AGAAAAAGAAAGAAGACTTCACAATGCAAATTAGTTTCTTGCAAGAGAAAATTAAAGTATATGAAATGAAAACACTTGGTAGACGGGACTCGAGTGATGATTGGGAGA

>STRN3-BRAF.S3B10

ACAAATTAAAATATGGCACGGAACTGAACCAAGGTGACTTGAAAATGCCAACCTTTGAGTCAGGATCAACCACAGGTTTGTCTGCTACCCCCCCTGCCTCATTACCTGGC

>TMEM178B-BRAF.T2B9

CCCCAGGAACCTCACTTTCAATATCACGAAGACCATCCGTCAGGATGAGTGGCATGCCCTACGACTTGATTAGAGACCAAGGATTTCGTGGTGATGGAGGATCAACCAC

>RP2-BRAF.R3B10

GCTGAGGATGCTCAAAGGGTTTTTCGGGAAAAAGCACCTGACTTCCTTCCTCTTCTGAACAAAGGATCAACCACAGGTTTGTCTGCTACCCCCCCTGCCTCATTACCTGGC

>KIAA1549-BRAF.K12B11

AAAACACTTGGTAGACGGGACTCGAGTGATGATTGGGAGATTCCTGATGGGC

>CCDC6-BRAF.C1B9

GGAGACCTACAAACTGAAGTGCAAGGCACTGCAGGAGGAGAACCGCGACCTGCGCAAAGCCAGCGTGACCATCGACTTGATTAGAGACCAAGGATTTCGTGGTGATG

>TANK-BRAF.T4B9

AGCCACAAGATAAAGTGATTTCAGGAATAGCAAGAGAAAAACTACCAAAGGACTTGATTAGAGACCAAGGATTTCGTGGTGATGGAGGATCAACCACAGGTTT

>RBMS3-BRAF.R11B11

AGCCAGCCAACATGATGGGCCCACTGACACAGCAGATGAATCACCTTTCGTTGGGCACAACAGGAACGAAAACACTTGGTAGACGGGACTCGAGTGATGATTGGGAGA

>MAD1L1-BRAF.M17B10

GTCCCAGCCGACCTTGAGGCTGCCGCCGCGAGTCTGCCATCGTCCAAGGAGGTGGCAGGATCAACCACAGGTTTGTCTGCTACCCCCCCTGCCTCATTACCTGGCTCACT

>KIAA1549-BRAF.K9B9

GATTTCGTGGTGATGGAGGATCAACCACAGGTTT

>BTF3L4-BRAF.B3B11

CTGATGACAAAAAGCTTCAGAGTTCTCTAAAAAAACTGGCTGTGAATAATATAGCTGGTATTGAAGAGAAAACACTTGGTAGACGGGACTCGAGTGATGATTGGGAGA

>SOX6-BRAF.S6B9

TTTTCCACATGACCAGCGGACTCTGGCAGCAGCTGCTGCTGCCCAACAGGGATTCCTCTTCCCCCCTGGAATAACATACAAACCAGGACTTGATTAGAGACCAAGGATTT

>AKAP9-BRAF.A21B10

TCATCTGCCAGCCTAATTTGGAGGTCAGAAGCAGAGGCATCTGTAAAGTCATGTGTCCATGAGGAACATACAAGAGGATCAACCACAGGTTTGTCTGCTACCCCCCCTG

>DYNC1I2-BRAF.D7B10

TCGACTTTCCTCCTCGAGAAATTGTCACGTATACAAAGGAAACTCAGACTCCAGTTATGGCTCAACCCAAAGAAGGATCAACCACAGGTTTGTCTGCTACCCCCCCTGCCT

>TRIM24-BRAF.T3B11

CGTGTATCAGAGCTCATCAGAGGGTAAAGTTCACAAAAGACCACACTGTCAGACAGAAAGAGGAAGTATCTCCAGAAAACACTTGGTAGACGGGACTCGAGTGATGAT

>MZT1-BRAF.M2B11

ACAAGGAATTAACCCAGAAGCTTTATCATCGGTTATTAAGGAGCTTCGCAAGGCTACTGAAGCACTGAAGAAAACACTTGGTAGACGGGACTCGAGTGATGATTGGGA

>NUP214-BRAF.N21B10

CCATGAAACAGGCACAACTGAGAAACTTCTTGGCCAAGAGGAAGACCCCACCAGTGAGATCCACTGCTCCAGGATCAACCACAGGTTTGTCTGCTACCCCCCCTGCCTCA

>ZC3HAV1-BRAF.Z3B10

CCGACGTGGTCCAGAACATCCAGGACATCTGCAACAGCAAGCACATGCAGAAGAATCCCCCAGGGCCCAGAGGATCAACCACAGGTTTGTCTGCTACCCCCCCTGCCTC

>MYRIP-BRAF.M16B9

TCTCCAAGGCTCCTCAACAAACAGGACTAAGGAAAGGAAAGGCACCACCAAGGATTTGATGGACTTGATTAGAGACCAAGGATTTCGTGGTGATGGAGGATCAACCAC

>AGAP3-BRAF.A10B11

AACAAGGAGTGGAAGAAGAAGTATGTGACGCTCTGTGACAACGGGCTGCTCACCTATCACCCCAGCCTGCATAAAACACTTGGTAGACGGGACTCGAGTGATGATTGG

>LSM12-BRAF.L3B9

CAGAAACCCCTCCTCCCCTAGCTTCACTCAATGTTAGTAAGGACTTGATTAGAGACCAAGGATTTCGTGGTGATGGAGGATCAACCACAGGTTT

>UBN2-BRAF.U3B11

ACACCGGAAGGATCGGCTACAAGATTTAATTGATATAGGCTTTGGCTATGATGAGACAGATCCATTTATTGATAACTCAGAGGCTAAAACACTTGGTAGACGGGACTCG

>CLIP2-BRAF.C6B11

CATCTGCGAGGTGGAGAAGGAGATTGCCCTGCTCAAGGCACAGCATGAGCAGAAAACACTTGGTAGACGGGACTCGAGTGATGATTGGGAGATTCCTGATGGGC

>FXR1-BRAF.F13B10

TGCGACAGATTGGTTCTAGGTCTTATAGCGGAAGAGGCAGAGGTCGTCGGGGACCTAATTACACCTCCGGTTATGGATCAACCACAGGTTTGTCTGCTACCCCCCCTGC

>RNF11-BRAF.R1B11

TCTCCCTGCTTCACGAGTCTCAGTCCGACCGGGCTAGCTTTGGCGAGGGGACGGAGCCGGATCAGGAGCCGCCGCCGCCATATCAGAAAACACTTGGTAGACGGGACT

>ERC1-BRAF.E12B10

GAGAAAGGAAGTCATCTTCATCCTCAGAAGACAGGAATCGAATGAAAACACT

>ERC1-BRAF.E17B8

ACGAGACCGATCCTCATCAGCTCCCAATGTGCA

>BRAF-BRAF.B3B9

CCACAGATGTGGCACGGAGCAACCCCAAGTCACCACAAAAACCTATCGTTAGAGTCTTCCTGCCCAACAAACAGAGGACAGTGGACTTGATTAGAGACCAAGGATTTCG

>BRAF-BRAF.B1B9

ACCAAGGATTTCGTGGTGATGGAGGATCAACCACAGGTTT

>BRAF-BRAF.B1B11

GACGGGACTCGAGTGATGATTGGGAGATTCCTGATGGGC

>BRAF-BRAF.B3B11

CCACAGATGTGGCACGGAGCAACCCCAAGTCACCACAAAAACCTATCGTTAGAGTCTTCCTGCCCAACAAACAGAGGACAGTGAAAACACTTGGTAGACGGGACTCGA

>BRCA1-BRCA1.B15B17.V16

TGAAGAGACTACTCATGTTGTTATGAAAACAGATGCTGAG

>BRCA1-BRCA1.B19B23.V20

GGGTGACCCAGTCTATTAAAGAAAGAAAAATGCTGAATGAGGGTGTCCACCCAATTGTGGTTGTGCAGCCAGATGCCTGGACAGAGGACAATGGCTTCCATG

>BRCA1-BRCA1.B10B14.V11
CCTGGAAGTAATTGTAAGCATCCTGAAATAAAAAAGCAAGAATATGAAGAAGTAGTTCAGACTGTTAATACAGATTTCTCTCCATATCTGATTTCAGATAACTTAGAACA
GCCTATGGGAATATTAACTTCACAGAAAAGTAGTGAATACCCTATAAGCCAGAATCCA

>BRCA1-BRCA1.B4B15.V5es

CCTTCACAGTGTCCTTTATGTAAGAATGATATAACCAAAAGGTCATCCCCTTCTAAATGCCCATCATTAGATGATAGGTGGTACATGCACAGTTGCTCTGGGAGTCTTCAG

>BRCA1-BRCA1.B7B12.V8es

GAACTGTGAGAACTCTGAGGACAAAGCAGCGGATACAACCTCAAAAGACGTCTGTCTACATTGAATTGGCAGAGGGATACCATGCAACATAACCTGATAAAGCTCCAG

>BRCA1-BRCA1.B7B14.V8es

GAACTGTGAGAACTCTGAGGACAAAGCAGCGGATACAACCTCAAAAGACGTCTGTCTACATTGAATTGGTATTAACTTCACAGAAAAGTAGTGAATACCCTATAAGCCA

>BRCA1-BRCA1.B15B18

AGTCTGGGCCACACGATTTGACGGAAACATCTTACTTGCCAAGGCAAGATCTAGATGCTGAGTTTGTGTGTGAACGGACACTGAAATATTTTCTAGGAATTGCGGGAGG

>BRCA1-BRCA1.B20B23.V21es

GAAGTCAGAGGAGATGTGGTCAATGGAAGAAACCACCAAGGTCCAAAGCGAGCAAGAGAATCCCAGGACAGAAAGGGTGTCCACCCAATTGTGGTTGTGCAGCCAGA

>BRCA2-BRCA2.B13B17.V14

GATGGATCATATGGAAACTGGCAGCTATGGAATGTGCC

>BRCA2-BRCA2.B1B3.V2

GTCAGCTTACTCCGGCCAAAAAAGAACTGCACCTCTGGAGCGGATTTAGGACCAATAAGTCTTAATTGGTTTGAAGAACTTTCTTCAGAAGCTCCACCCTATAATTCTGA

>BRCA2-BRCA2.B21B25.V22

CTTTCGTCTATTTGTCAGACGAATGTTACAATTTACTGGCA

>BRCA2-BRCA2.B7B10.V8

GGAATTCATTTAAAGTAAATAGCTGCAAAGACCACATTGG

>BRCA2-BRCA2.B11B11.D

TGATTTAATCAGAACTGAGCATAGTCTTCACTATTCACCTACGTCTAGACAA

>BRCA2-BRCA2.B11B22.V12

GCATGTCTAACAGCTATTCCTACCATTCTGATGAGGTATATAATGATTCAGGATATGGTTTATCAAGGGATGTCACAACCGTGTGGAAGTTGCGTATTGTAAGCTATTCA

>BRCA2-BRCA2.B11B27.V12

ACTTGATTCTGGTATTGAGCCAGTATTGAAGAATGTTGAAGATCAAAGTCCTTTATCACTTTGTATGGCCAAAAGGAAGTCTGTTTCCACACCTGTCTCAGCCCAGATGAC

>PTEN-BTAF1.P2B2

CTGCAGAAAGACTTGAAGGCGTATACAGGAACAATATTGATGATGTAGTAAGGCTAGATCGCCTTTTTATTTTACTGGATACTGGCACTACTCCTGTTACAAGAAAAGCT

>RAF1-C9orf153.R14C2

GACACCAGTCCAGCTGAGGACAATAGAGAAGCCACCCTTCC

>FGFR2-CASP7.F17C2

TGAGGATTCAGCAAATGAAGATTCAGTGGATGCTAAGCCAGAC

>FGFR2-CCAR2.F17C4

CAGCTTGCATGACTACTTTGGGGTTGTGGATGAAGAGG

>ESR1-CCDC170.E2C10

CTAAACAAATCCAGAGACCAACTGGAGAAGATGAAGGAGAAAGC

>ESR1-CCDC170.E2C6

GAAAGGGAAGTTAAGATCTTCCAAGAAAGGCTGCTTGCTGG

>ESR1-CCDC170.E2C7

TTGAACAGTTGGGAAAGGAGTCTGGGTTTCACCAGAAAGC

>ESR1-CCDC170.E2C8

AGTTGGACCAGATGGCTGCCGAACTTGGCTTTGACATGCGG

>FGFR2-CCDC6.F17C1

CAGCTCGGCGCTCCCCTGTGCTCGCCCGGCGCCCACTCATTCGCAGCCCG

>CDKN2A-CDKN2A.C1C3
CCTTCGGCTGACTGGCTGGCCACGGCCGCGGCCCGGGGTCGGGTAGAGGAGGTGCGGGCGCTGCTGGAGGCGGGGGCGCTGCCCAACGCACCGAATAGTTACGGT
CGGAGGCCGATCCAGACATCCCCGATTGAAAGAACCAGAGAGGCTCTGAGAAACCTCGGGAAACTTAGATCATCAGTCACCGAAGGTCCTACA

>FGFR2-CIT.F17C23

CGTAACAGCTGTACTGTAATCACAGACCTGGAGGAGCA

>FGFR2-COL14A1.F17C34

ATTGACTGCAAGCAAGTGGGTGAGAAGGCAATGAACGCATC

>FGFR2-CREB5.F17C8

TCAAATGGGAACATGAACACCATGGGACACATGATGGAGATGATG

>ESR1-DAB2.E6D3

GCTTACTGACCAACCTGGCAGACAGGGAGCTGGTTCACATGATCAACTGGGCGAAGAGGGTGCCAGGCCCTGAAAAGACAGATGAATATCTCTTAGCAAGGTTCAAAG

>EGFR-DYM.E1D3
GCCGGAGCGAGCTCTTCGGGGAGCAGCGATGCGACCCTCCGGGACGGCCGGGGCAGCGCTCCTGGCGCTGCTGGCTGCGCTCTGCCCGGCGAGTCGGGCTCTGGAG
GAAAAGAAAGTAGTGAGTTGAAACTCTTGGAGGAAGCAACCATTTCAGTCTGCAGGTCATTAGTTGAAAACAATCCTCGAACAG

>NTRK1-DYNC2H1.N17D85

CGCCACAGCATCAAGGATGTGCACGCCCGGCTGCAAGCCCTGGCCCAGAAGATCCCTTACAATACCTGAGAGGTCTTGTTGCCCGTGCCCTTGCAATACAGAACTGGGT

>CAND1-EGFR.C4E16

CTTACAAGTGCAATAGCAAAACAGGAAGATGTCTCTGTTCAGCTAGAAGCCTTGGATATTATGGCTGATATGTTGAGCAGATGCACTGGGCCAGGTCTTGAAGGCTGTC

>CDK6-EGFR.C2E23

CCAAGCCATATGACGGAATCCCTGCCAGCGAGATCTCCTC

>SEC61G-EGFR.S2E9

TGCTACGAATATTAAACACTTCAAAAACTGCACCTCCATCA

>EGFR-EGFR.E1E8.DelPositive.2

GCCGGAGCGAGCTCTTCGGGGAGCAGCGATGCGACCCTCCGGGACGGCCGGGGCAGCGCTCCTGGCGCTGCTGGCTGCGCTCTGCCCGGCGAGTCGGGCTCTGGAG
GAAAAGAAAGGTAATTATGTGGTGACAGATCACGGCTCGTGCGTCCGAGCCTGTGGGGCCGACAGCTATGAGATGGAGGAAGACGGC

>EGFR-EGFR.E24E28

GCTCTGTGCAGAATCCTGTCTATCACAATCAGCCTCTGAAC

>EGFR-EGFR.E3E5
CTGCAGATCATCAGAGGAAATATGTACTACGAAAATTCCTATGCCTTAGCAGTCTTATCTAACTATGATGCAAATAAAACCGGACTGAAGGAGCTGCCCATGAGAAATT
TACAGGGCCAAAAGTGTGATCCAAGCTGTCCCAATGGGAGCTGCTGGGGTGCAGGAGAGGAGAACTGCCAGAAACTGACCAAAATCATCT

>EGFR-EGFR.E13E15.vII
ACAGAGGTGAAAACAGCTGCAAGGCCACAGGCCAGGTCTGCCATGCCTTGTGCTCCCCCGAGGGCTGCTGGGGCCCGGAGCCCAACACCCTGGTCTGGAAGTACGCA
GACGCCGGCCATGTGTGCCACCTGTGCCATCCAAACTGCACCTACGGATGCACTGGGCCAGGTCTTGAAGGCTGTCCAACGAATGG

>WIPF2-ERBB2.W1E4

GTGTAGGCGGCAGAGGATTCGCTCCCAGAGCAGCTGCGGCCAGGTGGCAAAGCAAAGCTATATTCAAGACCACATGCAAAGCTACTCCCTGAGCAAAGAGTCACAGAT

>ERBB2-ERBB2.E19E21
CCTGACCTCTCCTACATGCCCATCTGGAAGTTTCCAGATGAGGAGGGCGCATGCCAGCCTTGCCCCATCAACTGCACCCACTCCCCTCTGACGTCCATCATCTCTGCGGT
GGTTGGCATTCTGCTGGTCGTGGTCTTGGGGGTGGTCTTTGGGATCCTCATCAAGCGACGGCAGCAGAAGATCCGGAAGTACACGATGC

>ATAD2-ERBB4.A8E15

TTTAAAGATGGCCCAAACTGTGTGGAAAAATGTCCAGATGG

>EZR-ERBB4.E12E18

TCAAGCTCAACTTCGTATTTTGAAAGAAACTGAGCTGAAGAGGG

>EGFR-ERP44.E1E5
GCCGGAGCGAGCTCTTCGGGGAGCAGCGATGCGACCCTCCGGGACGGCCGGGGCAGCGCTCCTGGCGCTGCTGGCTGCGCTCTGCCCGGCGAGTCGGGCTCTGGAG
GAAAAGAAAGCTGACATAGCCCAGAGATACAGGATAAGCAAATACCCAACCCTCAAATTGTTTCGTAATGGGATGATGATGAAGA

>FGFR2-FAM76A.F17F2

TGCAGGACTGAGTACCAGCAGGAGAGTAAAACCA

>BCR-FGFR1.B4F10
CAAGGAGTTCTATGATGGGCTCTTCCCCCGCGTGCAGCAGTGGAGCCACCAGCAGCGGGTGGGCGACCTCTTCCAGAAGCTGGTGTCTGCTGACTCCAGTGCATCCAT
GAACTCTGGGGTTCTTCTGGTTCGGCCATCACGGCTCTCCTCCAGTGGGACTCCCATGCTAG

>CNTRL-FGFR1.C40F10

CCATCACGGCTCTCCTCCAGTGGGACTCCCATGCTAG

>CUX1-FGFR1.C11F10

ATCACGGCTCTCCTCCAGTGGGACTCCCATGCTAG

>FGFR1OP-FGFR1.F5F10

AAGTGATCAGGCGCTGTCAACAGAAAGAAAAAGGGCCAACCACTGGGGAAGTGTCTGCTGACTCCAGTGCATCCATGAACTCTGGGGTTCTTCTGGTTCGGCCATCACG

>FGFR1OP-FGFR1.F6F10
GGGAAGGTGCACTTGATCTATCTGATGTACATTCTCCACCAAAGTCACCAGAGGGAAAAACAAGTGCACAGACAACACCAAGTAAGGTGTCTGCTGACTCCAGTGCAT
CCATGAACTCTGGGGTTCTTCTGGTTCGGCCATCACGGCTCTCCTCCAGTGGGACTCCCATGCTAG

>FGFR1OP-FGFR1.F7F10
CACAGACAACACCAAGTAAGATACCAAGGTATAAAGGACAAGGTAAGAAGAAGACAAGCGGGCAGAAGGCTGGTGACAAGGTGTCTGCTGACTCCAGTGCATCCAT
GAACTCTGGGGTTCTTCTGGTTCGGCCATCACGGCTCTCCTCCAGTGGGACTCCCATGCTAG

>LRRFIP1-FGFR1.L8F10

GAAGTCAAGGAGGCCCTGAAGCAAAGAGAGGAAATGCTCGAGGTGTCTGCTGACTCCAGTGCATCCATGAACTCTGGGGTTCTTCTGGTTCGGCCATCACGGCTCTCCT

>MYO18A-FGFR1.M33F10
GGCAGATGCCCAGCTCATGCTGGACCACCTGAAGAACAGTGCTCCCAGCAAGCGAGAGATTGCCCAGCTCAAGAACCAGGTGTCTGCTGACTCCAGTGCATCCATGAA
CTCTGGGGTTCTTCTGGTTCGGCCATCACGGCTCTCCTCCAGTGGGACTCCCATGCTAG

>TRIM24-FGFR1.T11F10
GCAGGATATGATGGAAAGGCTTTTGGTTCACCTATGATCGATTTGAGCTCACCAGTGGGAGGGTCTTATAATCTTCCCTCTCTTCCGGATGTGTCTGCTGACTCCAGTGC
ATCCATGAACTCTGGGGTTCTTCTGGTTCGGCCATCACGGCTCTCCTCCAGTGGGACTCCCATGCTAG

>BAG4-FGFR1.B2F6

GGGCAACTACACCTGCATTGTGGAGAATGAGTACGGC

>BAG4-FGFR1.B1F8
GGAGGAGGCGATGGCTACTATCCCTCGGGAGGCGCCTGGCCAGAGCCTGGTCGAGCCGGAGGAAGCCACCAGACTGCTGGAGTTAATACCACCGACAAAGAGATGG
AGGTGCTTCACTTAAGAAATGTCTCCTTTGAGGACGCAGGGGAGTATACGTGCTTGGCGGG

>ZMYM2-FGFR1.Z17F10

GGGTTCTTCTGGTTCGGCCATCACGGCTCTCCTCCAGTGGGACTCCCATGCTAG

>TPR-FGFR1.T22F10

CAAAACAAGTACGAGCAATGTGGAACAATATCAAGCAATGGTTACTAGTTTAGAAGAATCCCTGAACAAGGAAAAACAGGTGTCTGCTGACTCCAGTGCATCCATGAA
CTCTGGGGTTCTTCTGGTTCGGCCATCACGGCTCTCCTCCAGTGGGACTCCCATGCTAG

>FGFR1OP2-FGFR1.F4F10
AAGTACCGAGAACAAATGTTTAGATTGCTAATGGCTAGCAAAAAAGATGATCCGGGTATAATAATGAAGTTAAAAGAGCAGCACTCCAAGGTGTCTGCTGACTCCAGT
GCATCCATGAACTCTGGGGTTCTTCTGGTTCGGCCATCACGGCTCTCCTCCAGTGGGACTCCCATGCTAG

>WHSC1L1-FGFR1.W14F5

GCATGCAGTGCCGGCTGCCAAGACAGTGAAGTTCAAATGCC

>FN1-FGFR1.F22F3

TCTACACCATCCAAGTCCTGAGAGATGGACAGGAAAGAGATGCGCCAATTGTAAACAAAGTGGTGACACCCCAGCCCTGGGGAGCCCCTGTGGAAGTGGAGTCCTTCC[illegible]

>FN1-FGFR1.F23F3

AAGTGGAGTCCTTCCTGGTCCACCCCGGTGACCTGCTGCAGCTTCG

>FN1-FGFR1.F23F4

TGATGATGATGATGACTCCTCTTCAGAGGAGAAAGAAACAGATAACACC

>FN1-FGFR1.F28F5

AGTGCCGGCTGCCAAGACAGTGAAGTTCAAATGCC

>FN1-FGFR1.F22F4

ACTCCTCTTCAGAGGAGAAAGAAACAGATAACACC

>SQSTM1-FGFR1.S6F10
CAAGCCGGGTGGGAATGTTGAGGGCGCCACGCAGTCTCTGGCGGAGCAGATGAGGAAGATCGCCTTGGAGTCCGAGGGGCGCCCTGAGGTGTCTGCTGACTCCAGT
GCATCCATGAACTCTGGGGTTCTTCTGGTTCGGCCATCACGGCTCTCCTCCAGTGGGACTCCCATGCTAG

>CPSF6-FGFR1.C8int8F10
GCTGATGATCGTTGCAAAGTTCTTATTAGTTCTTTGCAAGATTGCCTTCATGGAATTGAGTCCAAGTCTTATGGTTCTGGATCAAGGTACACCAAATGTTTGACCACCTC
ACTTTATAAGGTGTCTGCTGACTCCAGTGCATCCATGAACTCTGGGGTTCTTCTGGTTCGGCCATCACGGCTCTCCTCCAGTGGGACTCCCATGCTAG

>ERVK3_1-FGFR1.E3F10
CAAGAAGCTGAGAAACTACTGGAGCGCCAGGGTCAGGCAAAAACCCCTGACTCCATGTTCTTGGCCATGCTAGCTGTAGTGTCCTGTGCGGTGTCTGCTGACTCCAGT
GCATCCATGAACTCTGGGGTTCTTCTGGTTCGGCCATCACGGCTCTCCTCCAGTGGGACTCCCATGCTAG

>RANBP2-FGFR1.R20F10int9

CTGGGGTTCTTCTGGTTCGGCCATCACGGCTCTCCTCCAGTGGGACTCCCATGCTAG

>SLC45A3-FGFR2.S1F1

GCGGGGCGCGGGGACAACACAGGTCGCGGAGG

>SLC45A3-FGFR2.S1F2

CCCGTAGAGGAAGTGTGCAGATGGGATTAACGTCCACATGG

>PARK2-FGFR2.P9F11

TTTGGGCAAGTGGTCATGGCGGAAGCAGTGGGAATTGACAAAG

>CD44-FGFR2.C1F3

GAAGTGTACGTGGCTGCGCCAGGGGAGTCGCTAGAGGTGCGC

>CTNNB1-FGFR2.C1F10

GGAGGAAGGTCTGAGGAGCAGCTTCAGTCCCCGCCGAGCCGCCACCGCAGGTCGAGGACGGTCGGACTCCCGCGGCGGGAGGAGCCTGTTCCCCTGAGGTTTCGGCT[illegible]

>PDHX-FGFR2.P1F7

GTAAGCCGCGGAGCTAATTGGAGATGGTTTCACAGCACGCAGTGGCTTCGGGAGCGATCGCCTCACCGGCCCATCCTCCAAGCCGGACTGCCGGCAAATGCCTCCACA[illegible]

>SNX19-FGFR2.S7F7

CTGCCGGCAAATGCCTCCACAGTGGTCGGAGGAGACGTAGA

>SNX19-FGFR2.S8F7

CAAATGCCTCCACAGTGGTCGGAGGAGACGTAGA

>ETV6-FGFR3.E5F9

CTGGAGTCCAACGCGTCCATGAGCTCCAACACACC

>WASF2-FGR.W1F2

ACCTGGAATGGGCTGTGTGTTCTGCAAGAAATTGGAGCCG

>ETV6-FLT3.E4F16

CACCATTCTTCCACCCTGGAAACTCTATACACACACAGCCGGAGGTCATACTGCATCAGAACCATGAAGAAGAAAAAGCAGACAGCTCTGAAAGAGAGGCACTCATGTC[illegible]

>ETV6-FLT3.E5F14

ATGCACCCTCTGATCCTGAACCCCCGGCACTCCGTGGATTTCAAACAGTCCAGGCTCTCCGAGGACGGGCTGCATAGGGAAGGGAGCCAGCTACAGATGGTACAGGTG[illegible]

>ETV6-FLT3.E5F16

ATGATCCGCCGCCTCTCCCCGGCTGAGAGAGCTCAGGGACCCAGGCCGCACCAGGAGAGCACACTGTCAGGACCAATTTACTTGATTTTTGAATACTGTTGCTATGGTGA

>SPTBN1-FLT3.S3F14

CTTGGCCTCATCTGGACCATCATCCTGCGCTTCCAGCAATTTAGGTATGAAAGCCAGCTACAGATGGTACAGGTGACCGGCTCCTCAGATAATGA

>ETV6-FLT3.E4ins16F14del11

CACCATTCTTCCACCCTGGAAACTCTATACACACACAGCCGGAGGTCATACTGCATCAGAACCATGAAGAAGGTTCTTTTATTTTGGGTGAAAGCCAGCTACAGATGGTAC

>ETV6-FLT3.E5ins6F14

AAGCCAGCTACAGATGGTACAGGTGACCGGCTCCTCAGATAATGA

>NOTCH1-GABBR2.N30G14.COSF1178
TGACCTGCGCATGTCTGCCATGGCCCCCACACCGCCCCAGGGTGAGGTTGACGCCGACTGCATGGACGTCAATGTCCGCGGGCCTGCCGGACCCAGCAGGACGGGAT
ATCTCCATCCGCCCTCTCCTGGAGCACTGTGAGAACACCCATATGACCATCTGGCTTGGCATCG

>ERBB2-GRB7.E29G10

GTCCCTTGGACAGCACCTTCTACCGCTCACTGCTGGAGGACGATGACATGGGGGACCTGGTGGTACGGGGTGCAGCTGTACAAGAATTACCAGCAGGCACAGTCTCGC

>ERBB2-GRB7.E30G11

GGCCTGGAGGAAGAAGACAAACCACCGCCTCAG

>NTRK3-HOMER1.N17H2

TGTCTTCCAAATTGACCCAAACACAAAGAAGAACTGGGTACC

>FGFR2-KCTD1.F17K2

TCCCCTGCATCTCCACTGAACAACCAAGGCATCCCTACTC

>UBE2L3-KRAS.U3K2.COSF1298.1

GGCGTAGGCAAGAGTGCCTTGACGATACAGCTAATTCAG

>MDM4-MDM4.M7M10

AGAGGTTACCTCTGAGGATGAGTGGCAGTGTACTGAATGCAA

>MDM4-MDM4.M8M10
GCCTTGGTGGTTTTTAGGAAACTTGAGAAGCAACTATACACCTAGAAGTAATGGCTCAACTGATTTACAGACAAATCAGGTGATTGAAGTGGGAAAAAATGATGACCT
GGAGGACTCTAAGTCCTTAAGTGATGATACCGATGTAGAGGTTACCTCTGAGGATGAGTGGCAGTGTACTGAATGCAA

>MDM4-MDM4.M3M10
CAAATCAATCAGGTACGACCAAAACTGCCGCTTTTGAAGATTTTGCATGCAGCAGGTGCGCAAGGTGAAATGTTCACTGTTAAAGAGGTGATTGAAGTGGGAAAAAAT
GATGACCTGGAGGACTCTAAGTCCTTAAGTGATGATACCGATGTAGAGGTTACCTCTGAGGATGAGTGGCAGTGTACTGAATGCAA

>MDM4-MDM4.M2M11

CCACCTCTGCTCAGTGTTCAACATCTGACAGTGCTTGCAGGATCTCTCCTGGACAAATCAATCAGGAAAATGAAGGAAATGATGTCCCTGATTGTCGAAGAACCATTTCG

>MDM4-MDM4.M5M7.S
TGGTGGAGATCTTTTGGGAGAACTACTGGGACGTCAGAGCTTCTCCGTGAAAGACCCAAGCCTCTCTATGATATGCTAAGAAAGAATCTTGTCACTTTAGCCACTGCTA
CTACAGCAAAGTGCAGAGGAAAGTTCCACTTCCAGAAAAAGAACTACAGAAGACGATATCCC

>BAIAP2L1-MET.B9M15

ACGGTTCATGCCGACAAGTGCAGTATCCTCTGACAG

>C8orf34-MET.C2M15

AAGGGAACCGTGGACAACTTCAAAGAACTTTGTCTGGATCTGCAGCTCTATGGGCAGAAAGTGAAAAATCAGATCAGTTTCCTAATTCATCTCAGAACGGTTCATGCCGA

>CAPZA2-MET.C4M11

CCACTCCTTCCCTGCAACAGCTGAATCTGCAACTCCC

>OXR1-MET.O9M13

TGAGGAGTCTGAAACAATTGAGGATTCTAGTAATCAAGCAGCAGCCAGAGAATGGGAGTGGAAGCAAGCAATTTCTTCAACCGTCCTTGGAAAAGTAATAGTTCAA

>PTPRZ1-MET.P1M2

GCGTTTCCTCGCTTGCATTCAGCTCCTCTGTGTTTGCCGCCTGGATAAACCTCTCATAATGAAGGCCCCCGCTGTGCTTGCACCTGGCATCCTCGTGCTCCTGTTTACCTTG

>PTPRZ1-MET.P3M2

CAGGGTTGGGATAAAACATCATTGGAAAACACATTCATTCATAACACTGGGAAAACAGATAAACCTCTCATAATGAAGGCCCCCGCTGTGCTTGCACCTGGCATCCTCGT

>PTPRZ1-MET.P8M2

CTGGCATCCTCGTGCTCCTGTTTACCTTGGTGCAGAGGAG

>TFG-MET.T5M15

AAAAATGTTATGTCAGCGTTTGGCTTAACAGATGATCAGGTTTCAGATCAGTTTCCTAATTCATCTCAGAACGGTTCATGCCGACAAGTGCAGTATCCTCTGACAG

>TPR-MET.T4M15

GGAAGAATTAGAAGCTGAGAAAAGAGACTTAATTAGAACCAATGAGAGACTATCTCAAGAACTTGAATACTTAACAGATCAGTTTCCTAATTCATCTCAGAACGGTTCAT

>LRRFIP1-MET.L19M15

TCAGAACGGTTCATGCCGACAAGTGCAGTATCCTCTGACAG

>DCTN1-MET.D26M15

TTTCCTAATTCATCTCAGAACGGTTCATGCCGACAAGTGCAGTATCCTCTGACAG

>TRIM4-MET.T6M15

TTGAAGCTGTAAAGGTGAAGACAGTGTGCCAGATACCATTGATGAAGGAAATGCTAAAGCGATTCCAAGATCAGTTTCCTAATTCATCTCAGAACGGTTCATGCCGACA

>EPS15-MET.E21M15

ACATCGGTAGAAACGTTGAAGCACAATGATCCTTTTGCTCCTGGTGGAACAGTTGTTGCAGCAAGCGATTCAGATCAGTTTCCTAATTCATCTCAGAACGGTTCATGCCG

>PPFIBP1-MET.P9M15

GGCTACAAGAAAAATTGGTTTGCAAGATGAAAGGAGAAGGGGTTGAAATTGTTGATAGAGATCAGTTTCCTAATTCATCTCAGAACGGTTCATGCCGACAAGTGCAGT

>ZKSCAN1-MET.Z3M15

CGAGCTTTGACCTTCATCACGAGGCCACCCAGTCCCACTTCAAACATTCGTCTCGGAAACCCCGCCTCTTACAGTCACGAGATCAGTTTCCTAATTCATCTCAGAACGGTT

>CAPZA2-MET.C1M6

TAATAAATTTGATTTAAAGAAAACTAGAGTTCTCCTTGGAAAT

>MET-MET.M13M15

AGTTTCCTAATTCATCTCAGAACGGTTCATGCCGACAAGTGCAGTATCCTCTGACAG

>MET-MET.M18M20.1
AAGATCTTATTGGCTTTGGTCTTCAAGTAGCCAAAGGCATGAAATATCTTGCAAGCAAAAAGTTTGTCCACAGAGACTTGGCTGCAAGAAACTGTATTGGTCCTTTGGC
GTGCTCCTCTGGGAGCTGATGACAAGAGGAGCCCCACCTTATCCTGACGTAAACACCTTTGATATAACTGTTTACTTGTTGCAAGGGAGA

>MET-MET.M17M20.1
TGAAGGGTCTCCGCTGGTGGTCCTACCATACATGAAACATGGAGATCTTCGAAATTTCATTCGAAATGAGACTCATTGGTCCTTTGGCGTGCTCCTCTGGGAGCTGATG
ACAAGAGGAGCCCCACCTTATCCTGACGTAAACACCTTTGATATAACTGTTTACTTGTTGCAAGGGAGA

>MET-MET.E2E3.AltT
GCGCAATATTCGGGTGGGAGTGACCCGATTTTCCAGGCAGAAAATGTGCTAGATTGGAGGTGAAGACCCTGGAGCCAGAGAGCCTAGGCTTAGTCCTAGCCCTGCAC
TGAAGACACTTCTGAGAAATTCATCAGGCTGTGAAGCGCGCCGTGATGAATATCGAACAGAGTTTACCACAGCTTTGCAGCGCGTTGACTT

>FGFR2-MGEA5.F17M12

CTCCCAGAGACCAACGTTCAAGCAGTTGGTAGAAGACTTGGATCGAATTCTCACTCTCACAACCAATGAGCGTTTGCTGCCAATTGATGGGGCAAATGATCTCTTTTTTC

>BRAF-MRPS33.B1M2

TTGCCACTCAAGAAATGTCCTCCCTTTCAGAATATGCC

>ESR1-MTHFD1L.E6M21

GCTTACTGACCAACCTGGCAGACAGGGAGCTGGTTCACATGATCAACTGGGCGAAGAGGGTGCCAGTGACCGAAGCTGGCTTTGGTGCTGACATCGGAATGGAGAAA

>MYB-NFIB.M8N9.COSF891
CCTGAGAAGGAAAAGCGAATAAAGGAATTAGAATTGCTCCTAATGTCAACCGAGAATGAGCTAAAAGGACAGCAGGTGCTACCACCTAACGGCAGTGGTCAAGTAGT
AGGGAAAGTGCCTGGCCATTTCACTCCTGTCTTGGCACCCTCTCCCCATCCCAGTGCAGTGCGA

>MYB-NFIB.M8N11.COSF784

GAATCAAAACGGCAGGCATTTATACCCCAGTACCAGTGAGGATACATTGGGA

>MYB-NFIB.M8N12.COSF786

TTCCTTTCCAAGTGTCAAATAGGACACCCATCTTACCGGCCAATG

>MYB-NFIB.M13N11.COSF876

GGCAGGCATTTATACCCCAGTACCAGTGAGGATACATTGGGA

>MYB-NFIB.M13N12.COSF877

GTGTCAAATAGGACACCCATCTTACCGGCCAATG

>MYB-NFIB.M15N8.COSF883

AGAAGATGAAGACAATGTTCTCAAAGCATTTACAGTACCTAAAAACAGGTCCCTGGCGAGCCCCTTGCAGTCATCTCAACTCGAACTCCACCTCCACCTTCACCGTTGCC

>MYB-NFIB.M15N9.COSF878

GCCATTTCACTCCTGTCTTGGCACCCTCTCCCCATCCCAGTGCAGTGCGA

>MYB-NFIB.M15N10.COSF879

TGGGACTAAGCCCAAGAGACCCATCCTTCCTACATCA

>MYB-NFIB.M15N11.COSF880

GCATTTATACCCCAGTACCAGTGAGGATACATTGGGA

>MYB-NFIB.M15N12.COSF884

AGAAGATGAAGACAATGTTCTCAAAGCATTTACAGTACCTAAAAACAGGTCCCTGGCGAGCCCCTTGCAGTCCTGGTACCTGGGCTAGCTTGGTTCCTTTCCAAGTGTCA

>MYBL1-NFIB.M8N11

AGGAACTTGAGATGCTTCTTATGTCAGCTGAGAATGAAGTTAGAAGAAAGCGAATTCCATCACTGAGGATTTGTGACTGGACCATGAATCAAAACGGCAGGCATTTATA

>MYBL1-NFIB.M8N12

AGGAACTTGAGATGCTTCTTATGTCAGCTGAGAATGAAGTTAGAAGAAAGCGAATTCCATCATCCTGGTACCTGGGCTAGCTTGGTTCCTTTCCAAGTGTCAAATAGGAC

>MYBL1-NFIB.M9N11

CTGTGTTATCCTCTTTGCAGACCATCCCAGAATTTGCAGAGACTCTAGAACTTATTGAATCTCTGAGGATTTGTGACTGGACCATGAATCAAAACGGCAGGCATTTATACC

>MYBL1-NFIB.M14N11

AAGAAGAATCAGGCACTCAACTGTTGACTGAAGACATTTCAGACATGCAGCTGAGGATTTGTGACTGGACCATGAATCAAAACGGCAGGCATTTATACCCCAGTACCAG

>MYBL1-NFIB.M15N11

GAAACCAAACCCTAACACTTCCAAAGTTGTCAAATTGGAAAAGAATCTTCAGCTGAGGATTTGTGACTGGACCATGAATCAAAACGGCAGGCATTTATACCCCAGTACCA

>FGFR2-NOL4.F17N7

CTCCCAGAGACCAACGTTCAAGCAGTTGGTAGAAGACTTGGATCGAATTCTCACTCTCACAACCAATGAGTCTCCTGCACATAGTTACTCCAGCTATGACTCTGGCAAAA

>SEC16A-NOTCH1.S1N27

CTGAGGTGTCTGTGCTCGTCGCCAGCGTCGGGTGGGCTTTCGCCCGCGGCTCCTGAGGGATCGGTCTCAGCCGCGCGGCTCCATCGTCTACCTGGAGATTGACAACCGG

>SEC16A-NOTCH1.S1N28

CTGAGGTGTCTGTGCTCGTCGCCAGCGTCGGGTGGGCTTTCGCCCGCGGCTCCTGAGGGATCGGTCTCAGCCGCGCGGGTGAGACCGTGGAGCCGCCCCCGCCGGCG

>MIR143HG-NOTCH1.M1N27

GCTCCATCGTCTACCTGGAGATTGACAACCGGCAGTGT

>NSD1-NOTCH4.N14N18

AGAAGGCATAGACGTCTCTTCCCTTTGCCACAATGGAGGC

>CD74-NRG1.C6N6

CACCATTGGCTCCTGTTTGAAATGAGCAGGCACTCCTTGGAGCAAAAGCCCACTGACGCTCCACCGAAAGCTACATCTACATCCACCACTGGGACAAGCCATCTTGTAAA

>CD74-NRG1.C8N6

CTTTCTGCCGACCCCTAGTTCCCTCTGCTCAGCCAAGCTTGTTATCAGCTTTCAGGGCCATGGCTACATCTACATCCACCACTGGGACAAGCCATCTTGTAAAATGTGCGG

>PDE7A-NRG1.P3N6

CCACTGGGACAAGCCATCTTGTAAAATGTGCGGAGAAGGAGAAAACT

>SDC4-NRG1.S4N6

GGTGTCAATGTCCAGCACTGTGCAGGGCAGCAACATCTTTGAGAGAACGGAGGTCCTGGCAGCTACATCTACATCCACCACTGGGACAAGCCATCTTGTAAAATGTGCG

>SLC3A2-NRG1.S5N6

GCTGCAGATCGACCCCAATTTTGGCTCCAAGGAAGATTTTGACAGTCTCTTGCAATCGGCTAAAAAAAAGACTACATCTACATCCACCACTGGGACAAGCCATCTTGTAA

>TENM4-NRG1.T12N2

GCCTCCCCGATTGAAAGAGATGAAAAGCCAGGAATCGGCT

>VAMP2-NRG1.V4N4

AACTGAAGGAGCATATGTGTCTTCAGAGTCTCCCATTAGAA

>PCM1-NRG1.P2N8

GCTTCTTGTAGAGGAAACAGCTTTGAAGTGTGGAGCGGGAAAGGAGCAGTTTCTGAGCTGCAAAAACTAGTTTCTAAACAGAGCATCTTGGGATTGAATTTATGGAGG

>FGFR1-NTM.F1N2

ACTCTCCCGAGGCGGAACCTCCACGCCGAGCGAGGAGTGCCCGTGCGCAGCGGAGATGCCACCTTCCCCAAAGCTATGGACAACGTGACGGTCC

>NOTCH1-NUP214.N2N25

CCCAGTGGCTGCTAACCAAGCCAAGCAGGGGTCTCTAATAAA

>BRD4-NUTM1.B15N2

GCCCCGTCTCCATCCCCTGCACTTCCCTTTCTCCCACCAAC

>BRD4-NUTM1.B11N2

CGCCCCGTCTCCATCCCCTGCACTTCCCTTTCTCCCACCAAC

>WHSC1L1-NUTM1.W7N2

AAAGCCAACGCAGAGTGTATCATCTCCTGAAGCAACATCTGGTTCTACAGCATCTGCATTGCCGGGACCGGATATGAGCATGAAACCTAGTGCCGCCCCGTCTCCATCCC

>BRD3-NUTM1.B10N2

CGCCCCGTCTCCATCCCCTGCACTTCCCTTTCTCCCACCAAC

>BRD4-NUTM1.B14N2del585

GCCAAGCCTCAGCAAGTCATCCAGCACCACCATTCACCCCGGCACCACAAGTCGGACCCCTACTCAACCGGTGACCGCTCCAAAATTTCCAAGGACGTTTATGAGAACTT

>FGFR2-OFD1.F17O3

CTCCCAGAGACCAACGTTCAAGCAGTTGGTAGAAGACTTGGATCGAATTCTCACTCTCACAACCAATGAGACACAACTTCGAAACCAGCTAATTCATGAGTTGATGCACC

>MYB-PCDHGA1.M9P2

CCCGGCACCAGCGGCTCCCAAAATGGCGATGACAC

>ESR1-PDE10A.E7P6

CATCACTCAGGGCACCACCGTCTCTGCTTATGTGGCCAAGT

>EGFR-PDP1.E1P2

GCCGGAGCGAGCTCTTCGGGGAGCAGCGATGCGACCCTCCGGGACGGCCGGGGCAGCGCTCCTGGCGCTGCTGGCTGCGCTCTGCCCGGCGAGTCGGGCTCTGGAG
GAAAAGAAAGGAATCCCAGTCAGAAGTTCCAGCCTGCCACTGTTCTCTGATGCCATGCCAGCACCAACTCAACT

>FNDC3B-PIK3CA.F3P2

TAGTTTTATATGTAAAACTTGCAAAGAATCAGAACAATGC

>TBL1XR1-PIK3CA.T1P2

GGGAATTTCCTTGTGCCTCCATTCCCGGGAGGGGGGAGCGGCGTTGGAGGCCACCGTTTCCAGGTTTCTGCTTTGGGACAACCATACATCTAATTCCTTAAAGTAGTTTT

>FGFR1-PLAG1.F1P2.COSF1108

ACTCTCCCGAGGCGGAACCTCCACGCCGAGCGAGGTTGCCTCTTGGTGCTGCCTTGGCCGTATTTGGCACCCAGAATGCTTCATTCTGTGACGGTCTATTAATAAGGTTG

>FGFR1-PLAG1.F1P3.COSF1110

ACTCTCCCGAGGCGGAACCTCCACGCCGAGCGAGATTGGCCAAAATGGGAAGGATTGGATTCCACTCTCTTCCACGAAGAGTCAATGGGACTGGCTAAGATCAA

>ESR1-POLH.E6P2

ACATGGACTGTTTTTTTGTTCAAGTGGAGCAGCG

>PAX8-PPARG.P10P2.COSF1219

GGCAGCTATGCCTCCTCTGCCATCGCAGGCATGGTGGCAGAAATGACCATGGTTGACACAGAGATGCCATTCTGGCCCACCAACTTTGGGATCAGCTCCGTGGATCTCT

>PAX8-PPARG.P7P2.COSF1223

TGGGATCAGCTCCGTGGATCTCTCCGTAATGGAAGACCACT

>PAX8-PPARG.P8P2.COSF1215

GCAACCTCTCGACTCACCAGACCTACCCCGTGGTGGCAGAAATGACCATGGTTGACACAGAGATGCCATTCTGGCCCACCAACTTTGGGATCAGCTCCGTGGATCTCTCC

>PAX8-PPARG.P9P2.COSF1217

TCCGTGTACGGGCAGTTCACGGGCCAGGCCCTCCTCTCAGAAATGACCATGGTTGACACAGAGATGCCATTCTGGCCCACCAACTTTGGGATCAGCTCCGTGGATCTCTC

>CREB3L2-PPARG.C2P2

CCAGGCTGAGCACAGCTACTCCCTGTGCGAGGAGCCTCGGGCCCAGTCGCCCTTCACCCACATTACCACCAGTGACAGCTTCAATGACGAAATGACCATGGTTGACACA
GAGATGCCATTCTGGCCCACCAACTTTGGGATCAGCTCCGTGGATCTCTCCGTAATGGAAGACCACT

>TSEN2-PPARG.T5P4

TGAAGCTTATCTATGACAGATGTGATCTTAACTGTCGGATCCAC

>TSEN2-PPARG.T6P6

GGAGAAGATAAAATCAAGTTCAAACACATCACCC

>FGFR2-PPHLN1.F17P3

CCACCACTGCTAGACAGACCTGGTGAAGGAAGCTACAATAG

>ATP1B1-PRKACA.A1P2

CTTAAAAAATGGGAAAGTCCCGCTCAGAACACAGCCCACTTGG

>DNAJB1-PRKACA.D1P2

CGCAAGCGCGAGATCTTCGACCGCTACGGGGAGGAAGTGAAAGAATTCTTAGCCAAAGCCAAAGAAGATTTTCTTAAAAAATGGGAAAGTCCCGCTCAGAACACAGCC

>DNAJB1-PRKACA.D2P2

TAAAAAATGGGAAAGTCCCGCTCAGAACACAGCCCACTTGG

>ATP1B1-PRKACB.A1P2

TTGAAAAAATGGGAGAATCCAACTCAGAATAATGCCGGACT

>NF1-PSMD11.N5P2

CTGTTTGTTCAGAAGACAATGTTGATGTTCATGATATAGAATTGTTACAGTATATCAATGTGGATTGTGCAAAATTAAAACGACTCCTGAAGGTGAAGCGTGACATTCAG

>EGFR-PSPH.E24P6

GGTTTTCCTAATATCTGGTGGCTTTAGGAGTATTGTAGA

>MYB-QKI.M9Q6

GATCATTACTGGGCCTGCGCCGGTTCTCCCACCAGCTGC

>CHD9-RAD51B.C2R8

TGGAGCCCTGGCTTCTCAGGCAGACCTGGTGTCTCCAGCTG

>EIF3E-RAD51B.E1R5

TGATGAGCATTTTGGCTACATTACCCACCAACATGGGAG

>HMGA2-RAD51B.H3R11

CTAAAGCAGCTCAAAAGAAAGCAGAAGCCACTGGAGAAAAACGGCCAAGAGGCAGACCTAGGAAATGGAGACAACATTTTGCTCTGTCACCCAAGCTGAACTGAACT
GGGCTCCAGAAATCCTCCCACCTCAGCCTCCTGAGCAGCTAGGACTACAGATGTGCCACCA

>HMGA2-RAD51B.H3R8.COSF981

GCCCTGGCTTCTCAGGCAGACCTGGTGTCTCCAGCTG

>NPC2-RAD51B.N1R9

TGTGTGATAGCCGCACTAGGAAATACCTGGAGTCACAGTGT

>PCNX-RAD51B.P1R8

GGAGCCCTGGCTTCTCAGGCAGACCTGGTGTCTCCAGCTG

>RB1-RB1.R20R24

CTGAGCACCCAGAATTAGAACATATCATCTGGACCCTTTTCCAGCACACCCTGCAGAATGAGTATGAACTCATGAGAGACAGGCATTTGGACCAAAATCTTAGTATCAA
TTGGTGAATCATTCGGGACTTCTGAGAAGTTCCAGAAAATAAATCAGATGGTATGTAACAGCGACCGTGTGCTCAAAAGAAGTGCTGAAG

>RB1-RB1.R21R23

ACCAATACCTCACATTCCTCGAAGCCCTTACAAGTTTC

>RB1-RB1.R21R25

TGTTCCATGTATGGCATATGCAAAGTGAAGAATATAGACCTTAAATTCAAAATCATTGTAACAGCATACAAGGATCTTCCTCATGCTGTTCAGGAGACTTCTGAGAAGTT
CCAGAAAATAAATCAGATGGTATGTAACAGCGACCGTGTGCTCAAAAGAAGTGCTGAAG

>C11orf95-RELA.C3R2

CCGAGGCGCTGTCTGAGCTCACCCAGTCCCCACCAGAACTGTTCCCCCTCATCTTCCCGGCAGAGCCAGCCCAGGCCTCTGGCCCCTATGTGGAGATCATTGAGCAGC

>C11orf95-RELA.C3R3

CCGAGGCGCTGTCTGAGCTCACCCAGTCCCCACCAGAGCCAGCCCAGGCCTCTGGCCCCTATGTGGAGATCATTGAGCAGC

>ZMYND8-RELA.Z21R2

GCCTCTGGCCCCTATGTGGAGATCATTGAGCAGC

>CCDC6-RET.C1R12.COSF1271

TTGGTTCTTGGAAAAACTCTAGGAGAAGGCGAATTTGG

>HOOK3-RET.H11R12.COSF1509

GAACTTGGTTCTTGGAAAAACTCTAGGAGAAGGCGAATTTGG

>KIF5B-RET.K15R12.COSF1232

AACTTGGTTCTTGGAAAAACTCTAGGAGAAGGCGAATTTGG

>KIF5B-RET.K16R12.COSF1230

GAAGAAAATGAAAAGGAGTTAGCAGCATGTCAGCTTCGTATCTCTCAAGAGGATCCAAAGTGGGAATTCCCTCGGAAGAACTTGGTTCTTGGAAAAACTCTAGGAGAA[illegible]

>KIF5B-RET.K22R12.COSF1253

CTTGGTTCTTGGAAAAACTCTAGGAGAAGGCGAATTTGG

>KIF5B-RET.K23R12.COSF1234

TCTTGGAAAAACTCTAGGAGAAGGCGAATTTGG

>KIF5B-RET.K24R8.COSF1236

ATCGCAAACGCTATCAGCAAGAAGTAGATCGCATAAAGGAAGCAGTCAGGTCAAAGAATATGGCCAGAAGAGGGCATTCTGCACAGATTGATGTGGCCGAGGAGGCG

>KIF5B-RET.K24R11.COSF1262

ATCGCAAACGCTATCAGCAAGAAGTAGATCGCATAAAGGAAGCAGTCAGGTCAAAGAATATGGCCAGAAGAGGGCATTCTGCACAGATTGATCCACTGTGCGACGAG[illegible]

>KTN1-RET.K29R12.COSF1513

CTTGGAAAAACTCTAGGAGAAGGCGAATTTGG

>NCOA4-RET.N7R12.COSF1491

GTTCTTGGAAAAACTCTAGGAGAAGGCGAATTTGG

>PRKAR1A-RET.P7R12.COSF1511

CTTGGTTCTTGGAAAAACTCTAGGAGAAGGCGAATTTGG

>TRIM24-RET.T9R12.COSF1521

CAACACAGATCAGCCTAGCTCAATTACGGCTCCAGCATATGCAGCAACAGGAGGATCCAAAGTGGGAATTCCCTCGGAAGAACTTGGTTCTTGGAAAAACTCTAGGAG[illegible]

>TRIM27-RET.T3R12.COSF1519

CGCTCAGCTAGAAGAGAAGCAGCAGCAGCCCACCAGGGAGCTCCTGCAGGAGGATCCAAAGTGGGAATTCCCTCGGAAGAACTTGGTTCTTGGAAAAACTCTAGGAG[illegible]

>CCDC6-RET.C8R11.COSF1518

CTGAGATGACCTTCCGGAGGCCCGCCCAGGCCTTCCCGGTCAGCTACTCCTCTTCCGG

>CUX1-RET.C10R12

ACTTGGTTCTTGGAAAAACTCTAGGAGAAGGCGAATTTGG

>ERC1-RET.E12R12

AACTTGGTTCTTGGAAAAACTCTAGGAGAAGGCGAATTTGG

>ERC1-RET.E17R12

GGTTCTTGGAAAAACTCTAGGAGAAGGCGAATTTGG

>ERC1-RET.E7R12

GGTTCTTGGAAAAACTCTAGGAGAAGGCGAATTTGG

>FKBP15-RET.F25R12

TTCTTGGAAAAACTCTAGGAGAAGGCGAATTTGG

>KIAA1468-RET.K10R12

CGTTATGTTAATGCTGGGACGCTGCCTGCCACACATTGTTCCCAATGTGCTATTGGCAAAGAGAGAGGAGGATCCAAAGTGGGAATTCCCTCGGAAGAACTTGGTTCTT

>TBL1XR1-RET.T9R11.NGS.1

CAGCATAAAGGCCCTATATTTGCATTAAAATGGAATAAGAAAGGAAATTTCATCCTAAGTGCTGGACTCCATGGAGAACCAGGTCTCCGTGGATGCCTTCAAGATCCTG
GAGGATCCAAAGTGGGAATTCCCTCGGAAGAACTTGGTTCTTGGAAAAACTCTAGGAGAAGGCGAATTTGG

>TBL1XR1-RET.T9R12

TTGGTTCTTGGAAAAACTCTAGGAGAAGGCGAATTTGG

>ACBD5-RET.A11R12

GAATTCCCTCGGAAGAACTTGGTTCTTGGAAAAACTCTAGGAGAAGGCGAATTTGG

>AFAP1-RET.A3R12

GGAATTCCCTCGGAAGAACTTGGTTCTTGGAAAAACTCTAGGAGAAGGCGAATTTGG

>AKAP13-RET.A35R12

CTGCCCAGAAACAGCTTGAGAGGGAACAGGAGCAGCTGCGCCGGGAGGCAGAGCGGCTCAGCCAGCGGCAGACAGAACGGGACCTGTGTCAGGAGGATCCAAAGT
GGGAATTCCCTCGGAAGAACTTGGTTCTTGGAAAAACTCTAGGAGAAGGCGAATTTGG

>AKAP13-RET.A36R12

TTCTTGGAAAAACTCTAGGAGAAGGCGAATTTGG

>CCDC6-RET.C1R13

GGAGACCTACAAACTGAAGTGCAAGGCACTGCAGGAGGAGAACCGCGACCTGCGCAAAGCCAGCGTGACCATCGAGTGAGCTGCGAGACCTGCTGTCAGAGTTCAAC

>CCDC6-RET.C2R12.1

AGGAGAAAGAAACCCTTGCTGTAAATTATGAGAAAGAAGAAGAATTCCCTCGGAAGAACTTGGTTCTTGGAAAAACTCTAGGAGAAGGCGAATTTGG

>CCDC6-RET.C8R11

TGGTTTCACGCCACCAACTTCACTGACTAGAGCTGGAATGTCTTATTACAATTCCCCGGGTCTTCACGTGCAGCACATGGGAACATCCCATGGTATCACAAGTTTGCCCA
CAAGCCACCCATCTCCTCAGCTGAGATGACCTTCCGGAGGCCCGCCCAGGCCTTCCCGGTCAGCTACTCCTCTTCCGG

>CCDC6-RET.C8R12

TGGTTTCACGCCACCAACTTCACTGACTAGAGCTGGAATGTCTTATTACACCACGGTGGCCGTGAAGATGCTGAAAGAGAACGCCTCCCCGAGTGAGCTGCGAGACCTG

>SPECC1L-RET.S10R12

ATCTCAAGTACCAAACCCTGCTGCAGCTGCAATTCCTCGAACGCCCCTGAGCCCAAGTCCTATGAAAACCCCTCCTGCAGCAGCTGTGTCCCCTATGCAGGAGGATCCA
AAGTGGGAATTCCCTCGGAAGAACTTGGTTCTTGGAAAAACTCTAGGAGAAGGCGAATTTGG

>CCDC6-RET.C1R12

GGAGACCTACAAACTGAAGTGCAAGGCACTGCAGGAGGAGAACCGCGACCTGCGCAAAGTGGGAATTCCCTCGGAAGAACTTGGTTCTTGGAAAAACTCTAGGAGAA

>CCDC6-RET.C1R11

GGAGACCTACAAACTGAAGTGCAAGGCACTGCAGGAGGAGAACCGCGACCTGCGCAAAGCCACCCATCTCCTCAGCTGAGATGACCTTCCGGAGGCCCGCCCAGGCCT

>CCDC6-RET.C1R11.1

GACCTTCCGGAGGCCCGCCCAGGCCTTCCCGGTCAGCTACTCCTCTTCCGG

>RUFY2-RET.R9R12

TTCTTGGAAAAACTCTAGGAGAAGGCGAATTTGG

>ERC1-RET.E11R12.COSF1507

CTTGGTTCTTGGAAAAACTCTAGGAGAAGGCGAATTTGG

>GOLGA5-RET.G7R12.COSF1503

GAGGGAGGAAATACAGAAGCTGATGGGCCAGATACATCAGCTCAGATCCGAATTACAGGAGGATCCAAAGTGGGAATTCCCTCGGAAGAACTTGGTTCTTGGAAAAA

>KIF5B-RET.K15R11.COSF1255.1

GATGACCTTCCGGAGGCCCGCCCAGGCCTTCCCGGTCAGCTACTCCTCTTCCGG

>NCOA4-RET.N6R12.COSF1340

CAGCAAATATTGGGCCCTTCCTGGAGAAGAGAGGCTGTATCTCCATGCCAGAGCAGGAGGATCCAAAGTGGGAATTCCCTCGGAAGAACTTGGTTCTTGGAAAAACTC

>PCM1-RET.P29R12.COSF1481

CAGCTGGACCGGCAAATTAAAGCAATTATGAAAGAAGTCATTCCTTTTTTGAAGGAGGATCCAAAGTGGGAATTCCCTCGGAAGAACTTGGTTCTTGGAAAAACTCTAG

>SPECC1L-RET.S10R11.NGS.1

ATCTCAAGTACCAAACCCTGCTGCAGCTGCAATTCCTCGAACGCCCCTGAGCCCAAGTCCTATGAAAACCCCTCCTCAGCTGAGATGACCTTCCGGAGGCCCGCCCAGGC

>TRIM33-RET.T16R12.COSF1525

AATTCCCTCGGAAGAACTTGGTTCTTGGAAAAACTCTAGGAGAAGGCGAATTTGG

>TRIM33-RET.T15R12

AATTCCCTCGGAAGAACTTGGTTCTTGGAAAAACTCTAGGAGAAGGCGAATTTGG

>TRIM33-RET.T11R12

GGTTCTTGGAAAAACTCTAGGAGAAGGCGAATTTGG

>CCDC6-RET.C2R11

AGGAGAAAGAAACCCTTGCTGTAAATTATGAGAAAGAAGAAGAATTCCTCACTAATGAGCTCTCCAGAAAATTGATGCAGATCCACTGTGCGACGAGCTGTGCCGCACG

>MYH13-RET.M35R12

CAGGCTGTCAGAGCAGGAGCTGCTGGACGCCAGCGACCGCGTGCAGCTCCTGCACTCCCAGGAGGATCCAAAGTGGGAATTCCCTCGGAAGAACTTGGTTCTTGGAAA

>CCDC6-RET.C5ins16R11

CCGCCCAGGCCTTCCCGGTCAGCTACTCCTCTTCCGG

>KIF5B-RET.K18R12

TTGGTTCTTGGAAAAACTCTAGGAGAAGGCGAATTTGG

>CCDC6-RET.C1R9

GGAGACCTACAAACTGAAGTGCAAGGCACTGCAGGAGGAGAACCGCGACCTGCGCAAAGCCAGCGTGACCATCGGATCACCAGGAACTTCTCCACCTGCTCTCCCAGC

>EIF3E-RSPO2.E1R2.COSF1307

CTCGCATCGCGCACTTTTTGGATCGGCATCTAGTCTTTCCGCTTCTTGAATTTCTCTCTGTAAAGGAGGTTCGTGGCGGAGAGATGCTGATCGCGCTGAACTGACCGGTG

>EIF3E-RSPO2.E1R3.COSF1309

TCAAAGGACAATGGGTGTAGCCGATGTCAACAGAAGTT

>EIF3E-RSPO2.E1ins351R2

CTGCAACCTCTGCCTCCTTAGTTCAAGCGATTCTCCTGCCTCAGCCTCCTGAGTAGCTGGTACTACAGGTTCGTGGCGGAGAGATGCTGATCGCGCTGAACTGACCGGTG

>GRHL2-RSPO2.G8R2

CCGGTGCGGCCCGGGGGTGAGTGGCGAGTCTCCCT

>PTPRK-RSPO3.P1R2.COSF1311

ACATGCTCAGATTACAATGGATGTTTGTCATGTAAGCCCA

>PTPRK-RSPO3.P7R2.COSF1313

GCTCCCAGGACCTCCACTAATCACCAGAACAAAATGTGCAGTGCATCCTAACGTTAGTCAAGGCTGCCAAGGAGGCTGTGCAACATGCTCAGATTACAATGGATGTTTG

>NOTCH1-SDCCAG3.N21S5

ACTGTGAGGACCTGGTGGACGAGTGCTCACCCAGCCCCTGCCAGAACGGGGCCACCTGCACGGACTACCTGGGCGGCTACTCCTGCAAGCTGAAAGATGAAAATTCTA

>PTEN-SHROOM4.P2S3

GGATGCCCTGAAGCAGCCACCACCATGCATTTCCCTTCTGAAG

>PTEN-SHROOM4.P3S4
CTGCAGAAAGACTTGAAGGCGTATACAGGAACAATATTGATGATGTAGTAAGGTTTTTGGATTCAAAGCATAAAAACCATTACAAGATATACAATCTTGACGTGTGTGT
GCAGTGGTGTCCACTCTCCCGGCATTGCAGCACCGAGAAAAGCAGCTCCATTGGCA

>FGFR2-SHTN1.F17S7

GAAGTTCTTGAACAGAGAAAAGTCTTAGAAAAATGCAATAGAGTGTCCATG

>BRAF-SLC26A4.B3S7

TGGTGATACCAATCTTGCTGATTTCACTGCTGGATTGCTCA

>NOTCH1-SNHG7.N2S4

CTGGAATAAAGAGTAACAAACCCCCTTGGAGGACTCTCCTGCCG

>BRAF-SUGCT.B1S13

GCCTCGTTATGGAGATGGAGCATCCAACTGTGGGGAAG

>FGFR1-TACC1.F17T7.COSF1362

CCCTCACAGAGACCCACCTTCAAGCAGCTGGTGGAAGACCTGGACCGCATCGTGGCCTTGACCTCCAACCAGGGGCTGCTGGAGTCCTCTGCAGAGAAGGCCCCTGTGT

>FGFR1-TACC1.F18T7

TCCGTCCCTGTCCCCTTTCCTGCTGGCAGGAGCCGGCTGCCTACCAGGGGCCTGGGCTGCTGGAGTCCTCTGCAGAGAAGGCCCCTGTGTCGGTGTCCTGTGGAGGTGA

>FGFR2-TACC3.F17T11

GCAGGTGTGAGGAGCTCCACGGGAAGAACCTGGAACTGGGGAAGATCATGGA

>CCDC127-TERT.C2T3

GTCTGCGTGAGGAGATCCTGGCCAAGTTCCTGCACTGGCT

>GLIS3-TERT.G3T3

CTATAAACTGCTGATCCACATGAGAGTCCACTCTGGGGAGAAGCCCAACAAGTGTACGGGGTTGGCTGTGTTCCGGCCGCAGAGCACCGTCTGCGTGAGGAGATCCTG[illegible]

>MTMR12-TERT.M7T3

CAAAGGCAACATGAAGTACAAAGCAGTGAGTGTCAACGAAGGCTATAAAGTCTGTGAGAGGGGTTGGCTGTGTTCCGGCCGCAGAGCACCGTCTGCGTGAGGAGATC[illegible]

>TRIO-TERT.T33T2

AGGCTGTGCGAGCGCGGCGCGAAGAACGTGCTGGCCTTC

>SLC12A7-TERT.S1T3

CATGCCCACCAACTTCACCGTGGTGCCCGTGGAGGCTCACGCCGACGGCGGCGGGGACGAGACTGCCGAGCGGACGGAGGCTCCGGGCACCCCCGAGGGCCCCGAG
CCCGAGCGCCCCAGCCCGGGGGTTGGCTGTGTTCCGGCCGCAGAGCACCGTCTGCGTGAGGAGATCCTGGCCAAGTTCCTGCACTGGCT

>TTLL7-TERT.T1T3

CGGGCTGGGCTTTCCTCACCCGGGGGTTGGCTGTGTTCCGGCCGCAGAGCACCGTCTGCGTGAGGAGATCCTGGCCAAGTTCCTGCACTGGCT

>FGFR2-TXLNA.F17T6

AAGCGCAAGGAGGTGACCTCGCACTTCCAGGTGACACTGAATGACATTC

>MYB-TYK2.M6T18

CAGCTGGCCACACTCACCAGCCAGTGTCTGACCTATGAG

>FGFR2-USP10.F17del11U5

CTCCCAGAGACCAACGTTCAAGCAGTTGGTAGAAGACTTGGATCGAATTCTCACTCTCAAGTTGCTGGAGAATGTAACCCTAATCCATAAACCAGTGTCGTTGCAACCCC[illegible]

>EGFR-VOPP1.E24V2

GGACTCTATCCAACCTATTATATATGCCGCTCCTACGAG

>ESR1-YAP1.E6Y4

GGAGAAATTTACTATATAAACCATAAGAACAAGACCACCTCTT

>MYBL1-YTHDF3.M8Y4int3

AGGAACTTGAGATGCTTCTTATGTCAGCTGAGAATGAAGTTAGAAGAAAGCGAATTCCATCAAGTAACAGCTATCCACCAATGTCAGATCCATACATGCCTAGTTACTAT[illegible]

>MYBL1-YTHDF3.M9Y4int3

CTGTGTTATCCTCTTTGCAGACCATCCCAGAATTTGCAGAGACTCTAGAACTTATTGAATCTAGTAACAGCTATCCACCAATGTCAGATCCATACATGCCTAGTTACTATGC

>FGFR1-ZNF703.F14Z2.COSF720

GGACAATGTGATGAAGATAGCAGACTTTGGCCTCGCACGGGACATTCACCACATCGACTACTATAAAAAGACAACCAACCTGGACGCCAAGAAGAGCCCCTTGGCGCT[illegible]

>MYB-QKI.M9Q5

TTGCGATTCTGAATGGCACCTACAGAGATGCCAACATTAAATCA

>MYB-QKI.M11Q5

GGAGCTTGCGATTCTGAATGGCACCTACAGAGATGCCAACATTAAATCA

>MYB-QKI.M15Q5

TGGAGCTTGCGATTCTGAATGGCACCTACAGAGATGCCAACATTAAATCA

>MYC.ENCTRL.E2E3

CCTGGTGCTCCATGAGGAGACACCGCCCACCACCAGCAGCGACTCTGAGGAGGAACAAGAAGATGAGGAAGAAATCGATGTTGTTTCTGTGGAAAAGAGGCAGGCTC[illegible]

>ITGB7.ENCTRL.E14E15

CCTATCTTGGATGATGGCTGGTGCAAAGAGCGGACCCTGGACAACCAGCTGTTCTTCTTCTTGGTGGAGGATGACGCCAGAGGCACGGTCGTGCTCAGAGTGAGACCC[illegible]

>EML4-BRAF.E6B10

CCCCTGCCTCATTACCTGGCTCACTAACTAACGTG

>TPM3-NTRK1.T7N13

TGGGAGCTGGGGGAGGGCGCCTTTGGGAAGGTCTTCCTTGCTGAGTGCCAC

>BAIAP2L1-BRAF.B12B9

CACCCGACTACTTGGAATGCTTGTCCATGGGGGCAGCTGCCGACAGGAGAGCAGATTCGGCCAGGACGACATCCACCTTTAAGGCCCCAGCGTCCAAGCCCGAGACC
GCGGCTCCTGACTTGATTAGAGACCAAGGATTTCGTGGTGATGGAGGATCAACCACAGGTTT

>EGFR-EGFR.E25E18.KDD

GGTGGATGCCGACGAGTACCTCATCCCACAGCAGGGCTTCTTCAGCAGCCCCTCCACGTCACGGACTCCCCTCCTGAGCTCTCTGCTTGTGGAGCCTCTTACACCCAGTG
GAGAAGCTCCCAACCAAGCTCTCTTGAGGATCTTGAAGGAAACTGAATTCAAAAAGATCAAAGTGCTGGGCT

>LMNA-NTRK1.L3N11

GCCTCTTCCTTTCTACGCTGCTCCTTGTGCTCAACAAATGTGGA

>CIC-NUTM1.C16N4

GTACTGGCAAGAAGGTGAAGGTGCGGCCCCCGCCCCTGAAGAAGACCTTTGACTCTGTGGACAAGTTCATGGAGTTTGAGGCTGAGGAGATGCAGATTCAGAACACA
CAGCTGATGAATGGGTCTCAGGGCCTGTCTCCTGCAACCCCTTTGAAACTTGATCCTCTAGGG

>TPM3-NTRK1.T8N10

GAGCACCTCTGTACACAAAGGATGCTGGACCAGACCCTGCTTGACCTGAATGAGATGTAGACACTAACAGCACATCTGGAGACCCGGTGGAGAAGAAGGACGAAACAC

>AR.E1E2.WT

TTCCACCCCAGAAGACCTGCCTGATCTGTGGAGATGAAGCTTCTG

>BRAF.E5E6.WT

TCACCTTAGCATTTTGTGACTTTTGTCGAAAGCTGCTTTTCCAG

>BRAF.E16E17.WT

AGTAACTGTCCAAAAGCCATGAAGAGATTAATGGCAGAGTGCCTCA

>BRCA2.E5E6.WT

TTGTCTTAGTGAAAGTCCTGTTGTTCTACAATGTACACATGTAACACCACAAAGAGATAAGTCAGTGGTATGTGGGAGTTTGTTTCATACACCAAAGTTTGTGAAGGGTC[illegible]

>BRCA2.E18E19.WT

GCTGGTATACCAAACTTGGATTCTTTCCTGACCCTAGACCTTTTCC

>EGFR.E6E7.WT
CAGTGACTGCTGCCACAACCAGTGTGCTGCAGGCTGCACAGGCCCCCGGGAGAGCGACTGCCTGGTCTGCCGCAAATTCCGAGACGAAGCCACGTGCAAGGACACCT
GCCCCCCACTCATGCTCTACAACCCCACCACGTACCAGATGGATGTGAACCCCGAGGGCAAATACAGCTTTGGTGCCACCT

>EGFR.E26E27.WT

GGCGCCTTGACTGAGGACAGCATAGACGACACCTTCCTC

>MDM4.E1E2.WT

AGCTGCCGTAAGTTTTACCAACAGACTGCAGTTTCTTCACTACCAAAA

>MET.E7E8.WT

TGGATCCTGTAATAACAAGTATTTCGCCGAAATACGGTCCTATGGCTGGTGGCACTT

>MET.E15E16.WT
CATGTAGTGATTGGGCCCAGTAGCCTGATTGTGCATTTCAATGAAGTCATAGGAAGAGGGCATTTTGGTTGTGTATATCATGGGACTTTGTTGGACAATGATGGCAAG
AAAATTCACTGTGCTGTGAAATCCTTGAACAGAATCACTGACATAGGAGAAGTTTCCCA

>RB1.E4E5.WT

TACTGAAATAAATTCTGCATTGGTGCTAAAAGTTTCTTG

>RB1.E26E27.WT

TGAATGATAGCATGGATACCTCAAACAAGGAAGAGAAATGA

>MET.M13M14M15.WT
AGATCAGAATTTCACAGGATTGATTGCTGGTGTTGTCTCAATATCAACAGCACTGTTATTACTACTTGGGTTTTTCCTGTGGCTGAAAAAGAGAAAGCAAATTAAAGATC
TGGGCAGTGAATTAGTTCGCTACGATGCAAGAGTACACACTCCTCATTTGGATAGGCTTGTAAGTGCCCGAAGTGTAAGCCCAACTACAGAAATGGTTTCAAATGAATC

>MET.M18M19M20.WT
AAGATCTTATTGGCTTTGGTCTTCAAGTAGCCAAAGGCATGAAATATCTTGCAAGCAAAAAGTTTGTCCACAGAGACTTGGCTGCAAGAAACTGTATGCTGGATGAAAA
ATTCACAGTCAAGGTTGCTGATTTTGGTCTTGCCAGAGACATGTATGATAAAGAATACTATAGTGTACACAACAAAACAGGTGCAAAGCTGCCAGTGAAGTGGATGGC
TTTGGAAAGTCTGCAAACTCAAAAGTTTACCACCAAGTCAGATGTGTGGTCCTTTGGCGTGCTCCTCTGGGAGCTGATGACAAGAGGAGCCCCACCTTATCCTGACGTA

>BRCA2.B1B2B3.WT
GTCAGCTTACTCCGGCCAAAAAAGAACTGCACCTCTGGAGCGGACTTATTTACCAAGCATTGGAGGAATATCGTAGGTAAAAATGCCTATTGGATCCAAAGAGAGGCC
AACATTTTTTGAAATTTTTAAGACACGCTGCAACAAAGCAGATTTAGGACCAATAAGTCTTAATTGGTTTGAAGAACTTTCTTCAGAAGCTCCACCCTATAATTCTGAACC

>EGFR.E3E4E5.WT
CTGCAGATCATCAGAGGAAATATGTACTACGAAAATTCCTATGCCTTAGCAGTCTTATCTAACTATGATGCAAATAAAACCGGACTGAAGGAGCTGCCCATGAGAAATT
TACAGGAAATCCTGCATGGCGCCGTGCGGTTCAGCAACAACCCTGCCCTGTGCAACGTGGAGAGCATCCAGTGGCGGGACATAGTCAGCAGTGACTTTCTCAGCAACA
TGTCGATGGACTTCCAGAACCACCTGGGCAGCTGCCAAAAGTGTGATCCAAGCTGTCCCAATGGGAGCTGCTGGGGTGCAGGAGAGGAGAACTGCCAGAAACTGACC

>ERBB2.E19E20E21.WT
CCTGACCTCTCCTACATGCCCATCTGGAAGTTTCCAGATGAGGAGGGCGCATGCCAGCCTTGCCCCATCAACTGCACCCACTCCTGTGTGGACCTGGATGACAAGGGCT
GCCCCGCCGAGCAGAGAGCCAGCCCTCTGACGTCCATCATCTCTGCGGTGGTTGGCATTCTGCTGGTCGTGGTCTTGGGGGTGGTCTTTGGGATCCTCATCAAGCGAC

>MDM4.M8M9M10.WT
GCCTTGGTGGTTTTTAGGAAACTTGAGAAGCAACTATACACCTAGAAGTAATGGCTCAACTGATTTACAGACAAATCAGGATGTGGGTACTGCCATTGTTTCAGATACT
ACAGATGACTTGTGGTTTTTGAATGAGTCAGTATCAGAGCAGTTAGGTGTTGGAATAAAAGTTGAAGCTGCTGATACTGAACAAACAAGTGAAGAAGTAGGGAAAGT
AAGTGACAAAAAGGTGATTGAAGTGGGAAAAAATGATGACCTGGAGGACTCTAAGTCCTTAAGTGATGATACCGATGTAGAGGTTACCTCTGAGGATGAGTGGCAGT

>RB1.R21R22R23.WT
TGTTCCATGTATGGCATATGCAAAGTGAAGAATATAGACCTTAAATTCAAAATCATTGTAACAGCATACAAGGATCTTCCTCATGCTGTTCAGGAGACATTCAAACGTGT
TTTGATCAAAGAAGAGGAGTATGATTCTATTATAGTATTCTATAACTCGGTCTTCATGCAGAGACTGAAAACAAATATTTTGCAGTATGCTTCCACCAGGCCCCCTACCT

>EGFR.E13E14E15.WT

ACAGAGGTGAAAACAGCTGCAAGGCCACAGGCCAGGTCTGCCATGCCTTGTGCTCCCCCGAGGGCTGCTGGGGCCCGGAGCCCTGAGCCAAGGGAGTTTGTGGAGA
ACTCTGAGTGCATACAGTGCCACCCAGAGTGCCTGCCTCAGGCCATGAACATCACCTGCACAGGACGGAACACCCTGGTCTGGAAGTACGCAGACGCCGGCCATGTGT

>BRCA1.B20B21B22B23.WT

GAAGTCAGAGGAGATGTGGTCAATGGAAGAAACCACCAAGGTCCAAAGCGAGCAAGAGAATCCCAGGACAGAAAGATCTTCAGGGGGCTAGAAATCTGTTGCTATG
GGCCCTTCACCAACATGCCCACAGATCAACTGGAATGGATGGTACAGCTGTGTGGTGCTTCTGTGGTGAAGGAGCTTTCATCATTCACCCTTGGCACAGGTGTCCACCC

>AR.A4A5A6.WT

TTGCTCTCTAGCCTCAATGAACTGGGAGAGAGACAGCTTGTACACGTGGTCAAGTGGGCCAAGGCCTTGCCTGGCTTCCGCAACTTACACGTGGACGACCAGATGGCT
GTCATTCAGTACTCCTGGATGGGGCTCATGGTGTTTGCCATGGGCTGGCGATCCTTCACCAATGTCAACTCCAGGATGCTCTACTTCGCCCCTGATCTGGTTTTCAATGA

>MDM4.M5M6M7.WT

TGGTGGAGATCTTTTGGGAGAACTACTGGGACGTCAGAGCTTCTCCGTGAAAGACCCAAGCCTCTCTATGATATGCTAAGAAAGAATCTTGTCACTTTAGCCACTGCTA
CTACAGATGCTGCTCAGACTCTCGCTCTCGCACAGGATCACAGTATGGATATTCCAAGTCAAGACCAACTGAAGCAAAGTGCAGAGGAAAGTTCCACTTCCAGAAAAA

>HMBS.ENCTRL.E8E9

CCAGCTCCCTGCGAAGAGCAGCCCAGCTGCAGAGAAAGTTCCCGCATCTGGAGTTCAGGAGTATTCGGGGAAACCTCAACACCCGGCTTCGGAAGCTGGACGAGCAGC

>EML4-ALK.E3p53insA20

CCGGAAGCACCAGGAGCTGCAAGCCATGCAGATGGAGCTGCAGAGCCCTGAG

>PPM1G-ALK.P1A18

GCTTCTCCGCCATGCAAGGCTGGCGCGTCTCCATGGAGTGATGGAAGGCCACGGGGAAGTGAATATTAAGCATTATCTAAACTGCAGTCACTGTGAGGTAG

>KANK2-ALK.K4A16

GGTGGTGGAGGTGGCTGGAATGATAACACTTCCTTGCTCTGGG

>KIF5B-ALK.K24A19

CCACACCTGCCACTCTCGCTGATCCTCTCTGTGGTGACCT

>MCFD2-ALK.M1A20

GGACCAGCTCCGGCATGCGGTCCCAGTGGCCCTCGGCGCGGCAGCGCTCCAGCTCGCTCTCCACCTTCAGTGTACCGCCGGAAGCACCAGGAGCTGCAAGCCATGCAG

>STK32B-ALK.S11A20

CCCGCTGAATGGACACCTGCAGCACTGTTTGGAGACTGTCCGGGAGGAATTCATCATATTCAACAGAGAGAATGTACCGCCGGAAGCACCAGGAGCTGCAAGCCATGC

>CAD-ALK.C35A20

GCTTCCTGATGGCCGCTTCCATCTGCCGCCCCGAATCCATCGAGCCTCCGACCCAGGTTTGCCAGTGTACCGCCGGAAGCACCAGGAGCTGCAAGCCATGCAGATGGAG

>GFPT1-ALK.G18A20.1

CTCAGCGTGATCCCTTTACAGTTGCTGGCTTTCCACCTTGCTGTGCTGAGAGGCTATGATGACCTCCTCCATCAGTGACCTGAAGGAGGTGCCGCGGAAAAACATCACC
CTCATTCGGGGTCTGGGCCATGGCGCCTTTGGGGAGGTGTATGAAGGCCAGGTGTCCGGAATGCCC

>TPR-ALK.T4A20

GGAAGAATTAGAAGCTGAGAAAAGAGACTTAATTAGAACCAATGAGAGACTATCTCAAGAACTTGAATACTTAACAGTGTACCGCCGGAAGCACCAGGAGCTGCAAGC

>EML4-ALK.E19A20

CCTTCCTGGCTGTAGGATCTCATGACAACTTTATTTACCTCTATGTAGTCTCTGAAAATGGAAGAAAATATAGCAGATATGGAAGGTGCACTCACCAGGAGCTGCAAGCC

>CCDC88A-ALK.C12A20

AGGAGCTGCAAGCCATGCAGATGGAGCTGCAGAGCCCTGAG

>KTN1-ALK.K43A19

ACACAGTTACAGCAGTTGCTTCAGGCGGTAAACCAACAGCTCACAAAGGAGAAAGAGCACTACCAGGTGTTAGTGTCACCCACCCCGGAGCCACACCTGCCACTCTCGC

>MSN-ALK.M11int12A20

CTCGAATCTCCCAGCTGGAGATGGCCCGACAGAAGAAGGAGAGTGAGGCTGTGGAGTGGCAGCAGAAGCAGGCAGCATGGGAGAAGGCACTCATGGTTTCGTCAAA
TACTTACTGGAGTTCTCTCAGGAGCTGCAAGCCATGCAGATGGAGCTGCAGAGCCCTGAG

>MYH9-ALK.M34A20del23

CTGGAGATGGACCTGAAGGACCTGGAGGCGCACATCGACTCGGCCAACAAGAACCGGGACGAAGCCATCAAACAGCTGCGGAAGCTGCAGGTCCTGCAAGCCATGCA

>EML4-ALK.E14A20.COSF1064.1

GGATGTTATTAACTGGAGGAGGGAAAGACAGAAAAATAATTCTGTGGGATCATGATCTGAATCCTGAAAGAGAAATAGAGCACCAGGAGCTGCAAGCCATGCAGATG

>EML4-ALK.E14del36A20

GGATGTTATTAACTGGAGGAGGGAAAGACAGAAAAATAATTCTGTGGGATCATGATCTGAATCCTGAAAGAGAAATAGAGATGGAGCTGCAGAGCCCTGAG

>EML4-ALK.E17int17Aint19E20

GACACTGTGCAGATTTTCATCCAAGTGGCACAGTGGTGGCCATAGGAACGCACTCAGGCAGGAGACAAAAACATGAAGTCAATTTTCCCAAAATTAAACTCATTAAAA
AATGTGGAATGCTGCCAGGCCATGTTGCAGCTGACCACCCACCTGCAGTGTACCGCCGGAAGCACCAGGAGCTGCAAGCCATGCAGATGGAGCTGCAGAGCCCTGAG

>BEND5-ALK.B3A20

GTCTGAAATGAAGGAGCTCCGTGACCTTAACCGGAGGCTCCAGGACGTGCTGCTCCTGCGGCTTGGCAGCGTGTACCGCCGGAAGCACCAGGAGCTGCAAGCCATGCA

>CLTC-ALK.C31ins63A20

TGCTTCAGAATCACTGAGAAAAGAAGAAGAACAAGCTACAGAGACACAACCCATTGTTTATGATGGGGTCTCGTCTGTCACCCAGGCTGGAGTGCAGTGGCGTGATCT
CGGCTCACTGCAACCTTTGTACCGCCGGAAGCACCAGGAGCTGCAAGCCATGCAGATGGAGCTGCAGAGCCCTGAG

>FN1-ALK.F20A19

CTGCAGCCTGCATCTGAGTACACCGTATCCCTCGTGGCCATAAAGGGCAACCAAGAGAGCCCCAAAGCCACTGGAGTCTTTACCACACTGTCACCCACCCCGGAGCCAC

>KCNQ5-ALK.K1A10

GCGGGTGCAGAACTACCTGTACAACGTGCTGGAGAGACCCCGCGGCTGGGCGTTCATCTACCACGCTTTCGTGTTCTGGCTGCAGATGGTCGCATGGTGGGGACAAGG

>ATRNL1-ALK.A19A20

ACCACAGGAAAGCAGTGTCAAGATTGTATGCCAGGTTATTATGGAGATCCAACCAATGGTGGACAGTGCACAGTGTACCGCCGGAAGCACCAGGAGCTGCAAGCCATG

>TRMT61B-ALK.T1A9

TTGGTGCTAGTGGAGAACAAAACCGGGAAGGAGCAAGGCAGG

>TFG-ALK.T7A19

CGGCTATGGTGCACAGCAGCCGCAGGCTCCACCTCAGCAGCCTCAACAGTATGGTATTCAGTATTCAGTGTCACCCACCCCGGAGCCACACCTGCCACTCTCGCTGATCC

>GTF3C2-ALK.G1A18

CGTCATAAGACCGCGACCAGACAGGCGGCGCCATCTTCGAACTTAGACTTCCGGAAGGACTTTGGCGAGGATTATCTAAACTGCAGTCACTGTGAGGTAG

>PPFIBP1-ALK.P8A20ins49
GTTAGTGAAATGGACAGTGAGAGACTTCAGTATGAAAAAAAGCTTAAATCAACCAAAGTTACTACGTGCTCGGCAATTTACACATTTCAATTCATTCGATCCTCAGTGTA
CCGCCGGAAGCACCAGGAGCTGCAAGCCATGCAGATGGAGCTGCAGAGCCCTGAG

>MYH9-ALK.M9A6ins10

CTGCAGAGCTCCTTCACTTGTTGGAATGGGACAGTCCTCCAGCTTGGG

>DCTN1-ALK.D29A20

GCCAGCTGCTGGAGACATTGAATCAATTGAGCACACACACGCACGTAGTAGACATCACTCGCACCAGCCCTGTGTACCGCCGGAAGCACCAGGAGCTGCAAGCCATGC

>HIP1-ALK.H30A20

AAAACTGGGAGAGCTTCGGAAAAAGCACTACGAGCTTGCTGGTGTTGCTGAGGGCTGGGAAGAAGTGTACCGCCGGAAGCACCAGGAGCTGCAAGCCATGCAGATG

>CLIP1-ALK.C13A20

CAAAAGGAGGAACAGTTTAACATGCTGTCTTCTGACTTGGAGAAGCTGAGAGAAAACTTAGCAGTGTACCGCCGGAAGCACCAGGAGCTGCAAGCCATGCAGATGGA

>BIRC6-ALK.B10A20

TGAGGAACAGGACACATTTGTTTCTGTGATTTACTGTTCTGGCACAGACAGGCTGTGTGCATGCACCAAAGTGTACCGCCGGAAGCACCAGGAGCTGCAAGCCATGCA

>TERT-ALK.T11A5

ACGGCCTATTCCCCTGGTGCGGCCTGCTGCTGGATACCCGGACCCTGGAGGTGCAGAGCGACTACTCCAGTTGGACAGTGCTCCAGGGAAGAATCGGGCGTCCAGACA

>CLIP4-ALK.C12A23

GGAGAGAGAGTGTTAGTGGTAGGACAGAGACTGGGCACCATTAGGTTCTTTGGGACAACAAACTTCGCTCCAGGCCCCGGTTCATCCTGCTGGAGCTCATGGCGGGG

>EML4-ALK.E6ins18A20

CGGAAGCACCAGGAGCTGCAAGCCATGCAGATGGAGCTGCAGAGCCCTGAG

>EML4-ALK.E13ins90A20

CCTGTAGGAAGTGGCCTGTGTAGTGCTTCAAGGGCCAGG

>EML4-ALK.E14ins2del52A20
GGATGTTATTAACTGGAGGAGGGAAAGACAGAAAAATAATTCTGTGGGATCATGATCTGAATCCTGAAAGAGAAATAGAGGTCCCTGAGTACAAGCTGAGCAAGCTC
CGCACCTCGACCATCATGACCGACTACAACCCCAACTACTGCTTTGCTGGCAAGACCTCCT

>EML4-ALK.E14ins124A20.1
GGATGTTATTAACTGGAGGAGGGAAAGACAGAAAAATAATTCTGTGGGATCATGATCTGAATCCTGAAAGAGAAATAGAGGGAAAGGTTCAGAGCTCAGGGGAGGA
TATGGAGATCCAGGGAGGCTTCCTGTAGGAAGTGGCCTGTGTAGTGCTTCAAGGGCCAGG

>EML4-ALK.E17ins65A20
GACACTGTGCAGATTTTCATCCAAGTGGCACAGTGGTGGCCATAGGAACGCACTCAGGCAGAGTAACAGATTCCCTGGATACCCTTTCAGAAATTTCTTCAAATAAACA
GAACCATTCTTATCCTGTGTACCGCCGGAAGCACCAGGAGCTGCAAGCCATGCAGATGGAGCTGCAGAGCCCTGAG

>EML4-ALK.E17ins68A20
GACACTGTGCAGATTTTCATCCAAGTGGCACAGTGGTGGCCATAGGAACGCACTCAGGCAGAGTCTTGCTCTGTCTCCCAGGCTGGAGTGCAGTGGCAATTTACACAT
TTCAATTCATTCGATCCTCAGTGTACCGCCGGAAGCACCAGGAGCTGCAAGCCATGCAGATGGAGCTGCAGAGCCCTGAG

>EML4-ALK.E17ins30A20_V8a

CAGGAGCTGCAAGCCATGCAGATGGAGCTGCAGAGCCCTGAG

>PRKAR1A-ALK.P2A20.NGS.2
GCACTGCTCGACCTGAGAGACCCATGGCATTCCTCAGGGAATACTTTGAGAGGTTGGAGAAGACCTCCTCCATCAGTGACCTGAAGGAGGTGCCGCGGAAAAACATCA
CCCTCATTCGGGGTCTGGGCCATGGCGCCTTTGGGGAGGTGTATGAAGGCCAGGTGTCCGGAATGCCC

>TRAF1-ALK.T6A20

GGAGCTGCAAGCCATGCAGATGGAGCTGCAGAGCCCTGAG

>PPP4R3B-ALK.P9A2
GCACCACTTTTGACCAATACTTCAGAAGACAAATGTGAAAAGGATAATATAGTTGGATCAAACAAAAACAACACAATTTGTCCCGGTCATAGCTCCTTGGAATCACCAA
CAAACATGCCTTCTCCTTCTCCTGATTATTTTACATGGAATCTCACCTGGATAATGAAAG

>DCTN1-ALK.D26A20

CGCCGGAAGCACCAGGAGCTGCAAGCCATGCAGATGGAGCTGCAGAGCCCTGAG

>EML4-ALK.E21A20

AGGAGCTGCAAGCCATGCAGATGGAGCTGCAGAGCCCTGAG

>A2M-ALK.A22A19

CAGTCATCAAGCCTCTGTTGGTTGAACCTGAAGGACTAGAGAAGGAAACAACATTCAACTCCCTACTTTGTCCATCAGTGTCACCCACCCCGGAGCCACACCTGCCACTC

>TPM1-ALK.T8A20.NGS

CTGAGACTCGGGCTGAGTTTGCGGAGAGGTCAGTAACTAAATTGGAGAAAAGCATTGATGACTTAGAAGTGTACCGCCGGAAGCACCAGGAGCTGCAAGCCATGCAG

>TPR-ALK.T15A20

GAGCTGCAAGCCATGCAGATGGAGCTGCAGAGCCCTGAG

>NCOA1-ALK.N21A1.NGS

CTCAAAACAGAAGCAGATGGAACCCAGCAGGTGCAACAGGTTCAGGTGTTTGCTGACGTCCAGTGTACAGTGAATCTGGTAGGCGGCTGTGGGGCTGCTCCAGTTCAA

>MEMO1-ALK.M2A7

ACAGCTAGAAGGTTGGCTTTCACAAGTACAGTCTACAAAAAGACCTGCTAGAGCCATTATTGCCCCGGAAACTGCCTGTGGGTTTTTACTGCAACTTTGAAGATGGCTTC

>GTF2IRD1-ALK.G7A20

CCTCTCATCCAGAACGTCCATGCCTCCAAGCGCATTCTCTTCTCCATCGTCCATGACAAGTCAGTGTACCGCCGGAAGCACCAGGAGCTGCAAGCCATGCAGATGGAGCT

>HIP1-ALK.H21A20

AGCGACGCCATTGCTCATGGTGCCACCACCTGCCTCAGAGCCCCACCTGAGCCTGCCGACTTGTACCGCCGGAAGCACCAGGAGCTGCAAGCCATGCAGATGGAGCTG

>HIP1-ALK.H28A20

GCGTTGTGGCCTCAACCATTTCCGGCAAATCACAGATCGAAGAGACAGTGTACCGCCGGAAGCACCAGGAGCTGCAAGCCATGCAGATGGAGCTGCAGAGCCCTGAG

>EML4-ALK.E13A20.COSF1062.2

GGAATGGAGATGTTCTTACTGGAGACTCAGGTGGAGTCATGCTTATATGGAGCAAAACTACTGTAGAGCCCACACCTGGGAAAGGACCTAAAGGAAGTGGCCTGTGTA

>EML4-ALK.E14A20.COSF477.1
GGATGTTATTAACTGGAGGAGGGAAAGACAGAAAAATAATTCTGTGGGATCATGATCTGAATCCTGAAAGAGAAATAGAGATATGCTGGATGAGCCCTGAGTACAAG
CTGAGCAAGCTCCGCACCTCGACCATCATGACCGACTACAACCCCAACTACTGCTTTGCTGGCAAGACCTCCT

>EML4-ALK.E17A20.COSF1366.2
GACACTGTGCAGATTTTCATCCAAGTGGCACAGTGGTGGCCATAGGAACGCACTCAGGCAGCATACTATGTATACAAGGGAGTTGCAGAGCCCTGAGTACAAGCTGA
GCAAGCTCCGCACCTCGACCATCATGACCGACTACAACCCCAACTACTGCTTTGCTGGCAAGACCTCCT

>EML4-ALK.E17A20.COSF1367.2

ACCATCATGACCGACTACAACCCCAACTACTGCTTTGCTGGCAAGACCTCCT

>EML4-ALK.E20A20.COSF730.1

CCGGAAGCACCAGGAGCTGCAAGCCATGCAGATGGAGCTGCAGAGCCCTGAG

>EML4-ALK.E2A20.COSF479.1

GATCCAGGGAGGCTTCCTGTAGGAAGTGGCCTGTGTAGTGCTTCAAGGGCCAGG

>EML4-ALK.E6A17

GTCGAAAATACCTTCAACACCCAAATTAATACCAAAAGTTACCAAAACTGCAGACAAGCATAAAGATGTCATCATCAACCAAGGCGGCAATGCAGCCTCAAACAATGAC

>EML4-ALK.E6A18

TATTAAGCATTATCTAAACTGCAGTCACTGTGAGGTAG

>EML4-ALK.E6bA20.AB374362
GTCGAAAATACCTTCAACACCCAAATTAATACCAAAAGTTACCAAAACTGCAGACAAGCATAAAGATGTCATCATCAACCAAGCAAAAATGTCAACTCGCGAAAAAAAC
AGCCAAGTGTACCGCCGGAAGCACCAGGAGCTGCAAGCCATGCAGATGGAGCTGCAGAGCCCTGAG

>EML4-ALK.E7A20.NGS

ACCCCAACTACTGCTTTGCTGGCAAGACCTCCT

>ACTG2-ALK.A2A18

CAGGCTTCGCAGGAGATGATGCCCCCCGGGCTGTCTTCCCCTCCATTGTGGGCCGCCCTCGCCACCAGTGATGGAAGGCCACGGGGAAGTGAATATTAAGCATTATCTA

>CLTC-ALK.C31A20.COSF470

GGAAGTGGCCTGTGTAGTGCTTCAAGGGCCAGG

>FN1-ALK.F23A19.COSF1301

TGTCTCCACCAACAAACTTGCATCTGGAGGCAAACCCTGACACTGGAGTGCTCACAGTCTCCTGGGAGAGGAGCACCACCCCAGTGTCACCCACCCCGGAGCCACACCT

>RNF213-ALK.R20A20

CAGGAGCTGCAAGCCATGCAGATGGAGCTGCAGAGCCCTGAG

>PPFIBP1-ALK.P12A20.COSF1461

GATCTTCGACAGTGCCTGAACAGGTACAAGAAAATGCAAGACACGGTGGTACTGGCCCAAGGTAAAAAAGTGTACCGCCGGAAGCACCAGGAGCTGCAAGCCATGCA

>SQSTM1-ALK.S5A20.COSF1051

AAGCCATGCAGATGGAGCTGCAGAGCCCTGAG

>STRN-ALK.S3A20.COSF1430

CAAGCCATGCAGATGGAGCTGCAGAGCCCTGAG

>TPM4-ALK.T7A20.COSF441

CTGACAAACTGAAAGAGGCTGAGACCCGTGCTGAATTTGCAGAGAGAACGGTTGCAAAACTGGAAAAGTGTACCGCCGGAAGCACCAGGAGCTGCAAGCCATGCAGA

>VCL-ALK.V16A20.COSF1057

AGGAGCTGCAAGCCATGCAGATGGAGCTGCAGAGCCCTGAG

>KIF5B-ALK.K15A20.COSF1381

GAGCAGCTGAGATGATGGCATCTTTACTAAAAGACCTTGCAGAAATAGGAATTGCTGTGGGAAATAATGATGTAAAGTGTACCGCCGGAAGCACCAGGAGCTGCAAGC

>KIF5B-ALK.K15A20.COSF1060.1

GAGCAGCTGAGATGATGGCATCTTTACTAAAAGACCTTGCAGAAATAGGAATTGCTGTGGGAAATAATGATGTAAAGCACCAGGAGCTGCAAGCCATGCAGATGGAGC

>KIF5B-ALK.K17A20.COSF1257

AAAGAAAAGACAGTTGGAGGAATCTGTCGATGCCCTCAGTGAAGAACTAGTCCAGCTTCGAGCACAAGTGTACCGCCGGAAGCACCAGGAGCTGCAAGCCATGCAGAT

>KIF5B-ALK.K24A20.COSF1058

CAGGAGCTGCAAGCCATGCAGATGGAGCTGCAGAGCCCTGAG

>TFG-ALK.T6A20.COSF428

TGCAAGCCATGCAGATGGAGCTGCAGAGCCCTGAG

>TFG-ALK.T4A20.COSF424

AGTGAATCGTTTATTGGATAGCTTGGAACCACCTGGAGAACCAGGACCTTCCACCAATATTCCTGAAAATGTGTACCGCCGGAAGCACCAGGAGCTGCAAGCCATGCAG

>TFG-ALK.T5A20.COSF426

AAAAATGTTATGTCAGCGTTTGGCTTAACAGATGATCAGGTTTCAGTGTACCGCCGGAAGCACCAGGAGCTGCAAGCCATGCAGATGGAGCTGCAGAGCCCTGAG

>TPM3-ALK.T7A20.COSF439

CAGAGACCCGTGCTGAGTTTGCTGAGAGATCGGTAGCCAAGCTGGAAAAGACAATTGATGACCTGGAAGTGTACCGCCGGAAGCACCAGGAGCTGCAAGCCATGCAG

>SEC31A-ALK.S21A20.COSF460

CAAATGCTGCTGGTCAGCTTCCCACATCTCCAGGTCATATGCACACCCAGGTACCACCTTATCCACAGCCACAGCTGTACCGCCGGAAGCACCAGGAGCTGCAAGCCATG

>SEC31A-ALK.S22A20.COSF459

AGGAGCTGCAAGCCATGCAGATGGAGCTGCAGAGCCCTGAG

>RANBP2-ALK.R18A20.COSF415

GAGCTGCAAGCCATGCAGATGGAGCTGCAGAGCCCTGAG

>NPM1-ALK.N4A20.COSF198

GCAAGCCATGCAGATGGAGCTGCAGAGCCCTGAG

>MSN-ALK.M11A20.COSF421

CTCGAATCTCCCAGCTGGAGATGGCCCGACAGAAGAAGGAGAGTGAGGCTGTGGAGTGGCAGCAGAAGCAGGAGCTGCAAGCCATGCAGATGGAGCTGCAGAGCCC

>KLC1-ALK.K9A20.COSF1276

CAAGCAGAAACACTGTACAAAGAGATTCTCACTCGTGCACATGAAAGGGAGTTTGGTTCTGTAGATGTGTACCGCCGGAAGCACCAGGAGCTGCAAGCCATGCAGATG

>CLTC-ALK.C31A20.COSF434

TGCTTCAGAATCACTGAGAAAAGAAGAAGAACAAGCTACAGAGACACAACCCATTGTTTATGTGTACCGCCGGAAGCACCAGGAGCTGCAAGCCATGCAGATGGAGCT

>EML4-ALK.E13A20.COSF408.1

CACCAGGAGCTGCAAGCCATGCAGATGGAGCTGCAGAGCCCTGAG

>EML4-ALK.E15A20.COSF413.1

GACCGACTACAACCCCAACTACTGCTTTGCTGGCAAGACCTCCT

>EML4-ALK.E18A20.COSF487.1

TGGATGCAGAAACCAGAGATCTAGTTTCTATCCACACAGACGGGAATGAACAGCTCTCTGTGATGCGCTACTCAATAGTGTACCGCCGGAAGCACCAGGAGCTGCAAG

>EML4-ALK.E20A20.COSF409.1

TGCAAGCCATGCAGATGGAGCTGCAGAGCCCTGAG

>EML4-ALK.E2A20.COSF478.1

CAAGCCATGCAGATGGAGCTGCAGAGCCCTGAG

>EML4-ALK.E6A19.COSF1296.1

GTCGAAAATACCTTCAACACCCAAATTAATACCAAAAGTTACCAAAACTGCAGACAAGCATAAAGATGTCATCATCAACCAAGTGTCACCCACCCCGGAGCCACACCTGC

>EML4-ALK.E6aA20.AB374361

GCAAGCCATGCAGATGGAGCTGCAGAGCCCTGAG

>ATIC-ALK.A7A20.COSF444

GGAAACAGTACAGCAAAGGCGTATCTCAGATGCCCTTGAGATATGGAATGAACCCACATCAGACCCCTGCCCAGCTGTACACACTGCAGCCCAAGCTTCCCATCACAGT
GTACCGCCGGAAGCACCAGGAGCTGCAAGCCATGCAGATGGAGCTGCAGAGCCCTGAG

>CARS-ALK.C17A20.COSF437

CACAGTCATGCCCTACCTTCAGGTGTTATCAGAATTCCGAGAAGGAGTGCGGAAGATTGCCCGAGAGCAAAAAGTGTACCGCCGGAAGCACCAGGAGCTGCAAGCCAT

>CD74-ROS1.C4R33.NGS

CCATGCAGAATGCCACCAAGTATGGCAACATGACATTTAATGGATCCTGCAGTAGTGTTTGCACATGGAAGTCCAAAAACCTGAAAGGAATATTTCAGTTCAGAGTAGT

>CD74-ROS1.C6R32.COSF1202

AAGGGAGTAAAAATTCAATACAGTGGGAGAAAGCTGA

>CD74-ROS1.C6R34.COSF1200

TATAGTTGGAATATTTCTGGTTGTTACAATCCCACTGA

>CEP85L-ROS1.C8R36

AAAGCAGTGTCAAGATAAAGAGACACAGTTAATATGCCAGAAAAAGAAAGAAAAGGAGTTAGTAACTACCGTTCAGAGTACTCTTCCAACCCAAGAGGAGATTGAAAA

>EZR-ROS1.E10R34.COSF1267

GCTGCAGGACTATGAGGAGAAGACAAAGAAGGCAGAGAGAGATGATTTTTGGATACCAGAAACAAGTTTCATACTTACTATTATAGTTGGAATATTTCTGGTTGTTACA

>GOPC-ROS1.G4R36.COSF1188

CCCTTCGTAGACATATAGCTGTTCTCCAGGCTGAAGTATATGGGGCGAGACTAGCTGCCAAGTACTTGGATAAGGAACTGGCAGGAAGTACTCTTCCAACCCAAGAGGA

>GOPC-ROS1.G8R35.COSF1139

TGGTGCTAGTTGCAAAGACACAAGTGGGGAAATCAAAGTATTACAAGTCTGGCATAGAAGATTAAAGAATCAAAAAAGTGCCAAGGAAGGGGTGACAGTGCTTATAAA

>KDELR2-ROS1.K5R35

AGGGGTGACAGTGCTTATAAACGAAGACAAAGAGTTGGCTGA

>LRIG3-ROS1.L16R35.COSF1269

TCACATCTTCAGGTGCTGGATTTTTCTTACCACAACATGACAGTAGTGTCTGGCATAGAAGATTAAAGAATCAAAAAAGTGCCAAGGAAGGGGTGACAGTGCTTATAAA

>SDC4-ROS1.S2R32.COSF1265

CGGGCAGGAATCTGATGACTTTGAGCTGTCTGGCTCTGGAGATCTGGCTGGAGTCCCAAATAAACCAGGCATTCCCAAATTACTAGAAGGGAGTAAAAATTCAATACAG

>SDC4-ROS1.S2R34

CGGGCAGGAATCTGATGACTTTGAGCTGTCTGGCTCTGGAGATCTGGATGATTTTTGGATACCAGAAACAAGTTTCATACTTACTATTATAGTTGGAATATTTCTGGTTG

>SDC4-ROS1.S4R32.COSF1278

GGTGTCAATGTCCAGCACTGTGCAGGGCAGCAACATCTTTGAGAGAACGGAGGTCCTGGCAGCTGGAGTCCCAAATAAACCAGGCATTCCCAAATTACTAGAAGGGAG

>SDC4-ROS1.S4R34.COSF1280

GGTGTCAATGTCCAGCACTGTGCAGGGCAGCAACATCTTTGAGAGAACGGAGGTCCTGGCAGATGATTTTTGGATACCAGAAACAAGTTTCATACTTACTATTATAGTT

>SLC34A2-ROS1.S13R32.COSF1259

GGCTCCTGAGACCTTTGATAACATAACCATTAGCAGAGAGGCTCAGGCTGGAGTCCCAAATAAACCAGGCATTCCCAAATTACTAGAAGGGAGTAAAAATTCAATACAG

>SLC34A2-ROS1.S13R34.COSF1261

GGCTCCTGAGACCTTTGATAACATAACCATTAGCAGAGAGGCTCAGGATGATTTTTGGATACCAGAAACAAGTTTCATACTTACTATTATAGTTGGAATATTTCTGGTTG

>SLC34A2-ROS1.S13R36

GGCTCCTGAGACCTTTGATAACATAACCATTAGCAGAGAGGCTCAGGGTGAGGTTTTGGAGAAGTGTATGAAGGAACAGCAGTGGACATCTTAGGAGTTGGAAGTGG

>SLC34A2-ROS1.S4R32.COSF1196

CGTGTGCTCCCTGGATATTCTTAGTAGCGCCTTCCAGCTGGTTGGAGCTGGAGTCCCAAATAAACCAGGCATTCCCAAATTACTAGAAGGGAGTAAAAATTCAATACAG

>SLC34A2-ROS1.S4R34.COSF1198

CGTGTGCTCCCTGGATATTCTTAGTAGCGCCTTCCAGCTGGTTGGAGATGATTTTTGGATACCAGAAACAAGTTTCATACTTACTATTATAGTTGGAATATTTCTGGTTGT

>TPM3-ROS1.T7R35.COSF1273

GAAGGGGTGACAGTGCTTATAAACGAAGACAAAGAGTTGGCTGA

>CLIP1-ROS1.C19R36

CAGGGACGAAGTCACAAGTCATCAAAAGCTGGAAGAAGAAAGATCTGTGCTCAATAATCAGTTGTTAGAAATGAAAAAAAGTACTCTTCCAACCCAAGAGGAGATTGA

>ERC1-ROS1.E11R36

CAGGCAGAAGTTGATCGACTCTTAGAAATCTTGAAGGAGGTGGAAAATGAGAAGAATGACAAAGATAAGAAGATAGCTGAGTTGGAAAGTACTCTTCCAACCCAAGA

>HLA_A-ROS1.H7R34

GCTGCAAGCAGTGACAGTGCCCAGGGCTCTGATGTGTCCCTCACAGCTTGTAAAGATGATTTTTGGATACCAGAAACAAGTTTCATACTTACTATTATAGTTGGAATATTT

>KIAA1598-ROS1.K11R36

GAAACGAGTGAACCAGTCTGAGAATTCAGTACCTCCACCACCTCCTCCTCCACCACCACTTCCCCCTCCACCTCCCAATCCTATCCGTACTCTTCCAACCCAAGAGGAGATT

>MYO5A-ROS1.M23R35

GTGCCAAGGAAGGGGTGACAGTGCTTATAAACGAAGACAAAGAGTTGGCTGA

>PPFIBP1-ROS1.P9R35

GACAGTGCTTATAAACGAAGACAAAGAGTTGGCTGA

>PWWP2A-ROS1.P1R36

GTCACGCTGGACCACATCATTGAGGACGCGCTTGTCGTGTCGTTCCGCTTCGGGGAGAAGCTCTTCTCCGGGGTCCTCATGGATCTGTCCAAAAGTACTCTTCCAACCCA

>TPM3-ROS1.T3R36

TACTCTTCCAACCCAAGAGGAGATTGAAAATCTTCCTGCCTTCC

>ZCCHC8-ROS1.Z2R36

CTCCGCGCCGAGAATCAAGAACTTAAACGAAAATTGAACATTCTGACTCGACCGAGTACTCTTCCAACCCAAGAGGAGATTGAAAATCTTCCTGCCTTCC

>AXL-MBIP.A20M4.1

AGATTGACAGACGAATATCTGCATTTATTGAAAGAAAGCAAGCTGAAATCAA

>TFG-ROS1.T4R35

GGAAGGGGTGACAGTGCTTATAAACGAAGACAAAGAGTTGGCTGA

>CD74-ROS1.C6R35.COSF1478

GAAGGGGTGACAGTGCTTATAAACGAAGACAAAGAGTTGGCTGA

>EZR-ROS1.E10R35

GCTGCAGGACTATGAGGAGAAGACAAAGAAGGCAGAGAGAGTCTGGCATAGAAGATTAAAGAATCAAAAAAGTGCCAAGGAAGGGGTGACAGTGCTTATAAACGAA

>MSN-ROS1.M9R34

TAGTTGGAATATTTCTGGTTGTTACAATCCCACTGA

>CCDC6-ROS1.C5R35.1

CGGCTGAAGAAGCAACTGAGAGCTGCTCAGTTACAGCTCTGGCATAGAAGATTAAAGAATCAAAAAAGTGCCAAGGAAGGGGTGACAGTGCTTATAAACGAAGACAA

>KDELR2-ROS1.K5Rintron34
CCAGACCATCCTATACTGTGACTTCTTCTACTTGTACATTACAAAAGTACTCAAGGGAAAGAAGCTCAGCTCAAAGGGAATGGTCAAGTACTGATTGGACTTTTAGAGTT
ATCACAGTGATCATACATTCTTGTTTATTTCTGTTTCAGCACAAATAATAATAGCACTT

>ADAMTS16-TERT.A8T3

CCGTCTGCGTGAGGAGATCCTGGCCAAGTTCCTGCACTGGCT

>AR-OPHN1.A3O3

GTCCATCTTGTCGTCTTCGGAAATGTTATGAAGCAGGGATGACTCTGGGAGATTATTCTTCTGCTGTTCAGAAATTTTCCCAGACGCTGCAGTCATTTCAGTTTGATTTCA

>NCOR2-ROS1.N7R36

TTCCAACCCAAGAGGAGATTGAAAATCTTCCTGCCTTCC

>TMEM106B-ROS1.T3R35
AGATGGAAGAAATGGAGATGTCTCTCAGTTTCCATATGTGGAATTTACAGGAAGAGATAGTGTCACCTGCCCTACTTGTCAGGGAACAGGAAGAATTCCTAGGGTCTG
GCATAGAAGATTAAAGAATCAAAAAAGTGCCAAGGAAGGGGTGACAGTGCTTATAAACGAAGACAAAGAGTTGGCTGA

>YWHAE-ROS1.Y4R36

CTATTCGCTTAGGTCTTGCTCTCAATTTTTCCGTATTCTACTACGAAATTCTTAATTCCCCTGACCGTGCCTGCAGTACTCTTCCAACCCAAGAGGAGATTGAAAATCTTCC

>NFKB2-ROS1.N13R36

CCCAAGAGGAGATTGAAAATCTTCCTGCCTTCC

>FGFR3-TACC3.F17T10.COSF1434

GGACCTGGACCGTGTCCTTACCGTGACGTCCACCGACGTGCCAGGCCCACCCCCAGGTGTTCCCGCGCCTGGGGGCCCACCCCTGTCCACCGGACCTATAGTGGACCTG

>FGFR3-BAIAP2L1.F17B2.COSF1346

GGACCTGGACCGTGTCCTTACCGTGACGTCCACCGACAATGTTATGGAACAGTTCAATCCTGGGCTGCGAAATTTAATAAACCTGGGGAAAAATTATGAGAAAGCTGTA

>FGFR3-TACC3.F17T8.COSF1353

GGACCTGGACCGTGTCCTTACCGTGACGTCCACCGACTTTAAGGAGTCGGCCTTGAGGAAGCAGTCCTTATACCTCAAGTTCGACCCCCTCCTGAGGGACAGTCCTGGT

>FGFR3-TACC3.F17T11.COSF1348

GGACCTGGACCGTGTCCTTACCGTGACGTCCACCGACGTAAAGGCGACACAGGAGGAGAACCGGGAGCTGAGGAGCAGGTGTGAGGAGCTCCACGGGAAGAACCTG

>FGFR3-TACC3.F15T11

GGCCTTGTTTGACCGAGTCTACACTCACCAGAGTGACGTGTAAAGGCGACACAGGAGGAGAACCGGGAGCTGAGGAGCAGGTGTGAGGAGCTCCACGGGAAGAACC

>FGFR3-TACC3.F16T10.COSF1359

ACCCCTGTCCACCGGACCTATAGTGGACCTGCTCCAG

>FGFR3-TACC3.F16T11.COSF1348

GGTGTGAGGAGCTCCACGGGAAGAACCTGGAACTGGGGAAGATCATGGA

>FGFR3-TACC3.F17T13.NGS

TAAAAGAAAAAGACCAACTTACCACAGATCTGAACTCCAT

>FGFR3-TACC3.F17T5

GGACCTGGACCGTGTCCTTACCGTGACGTCCACCGACGCAGCTGCATTCAGCCTCAGCGGAGGACACGCCTGTGGTGCAGTTGGCAGCCGAGACCCCAACAGCAGAGA

>FGFR3-TACC3.F17T6

GGACCTGGACCGTGTCCTTACCGTGACGTCCACCGACGAGAGAGCCTTGAACTCTGCCAGCACCTCGCTTCCCACAAGCTGTCCAGGCAGTGAGCCAGTGCCCACCCAT

>FGFR3-TACC3.F17T9

GGACCTGGACCGTGTCCTTACCGTGACGTCCACCGACCATGCACGGTGCAAATGAGACTCCCTCAGGACGTCCGCGGGAAGCCAAGCTTGTGGAGTTCGATTTCTTGGG

>FGFR3-TACC3.F18T7.NGS

TTCGACCCCCTCCTGAGGGACAGTCCTGGTAGACC

>FGFR3-AES.F17A2

GGACCTGGACCGTGTCCTTACCGTGACGTCCACCGACGGCTCCTCGCACCTACCCCAGCAACTCAAATTCACCACCTCGGACTCCTGCGACCGCATCAAAGACGAATTTC

>FGFR3-ELAVL3.F17E2

GGACCTGGACCGTGTCCTTACCGTGACGTCCACCGACCAGATACTGGGGGCCATGGAGTCTCAGGTGGGGGGGGGCCCGGCCGGCCCGGCCCTGCCCAACGGGCCAC

>FGFR3-TACC3.F14T11

AGGTGTGAGGAGCTCCACGGGAAGAACCTGGAACTGGGGAAGATCATGGA

>FGFR3-TACC3.F18T4and5

GGCGCCTTTCGAGCAGTACTCCCCGAGCCAGCAGCTGCATTCAGCCTCAGCGGAGGACACGCCTGTGGTGCAGTTGGCAGCCGAGACCCCAACAGCAGAGAGCAAGG

>FGFR3-TACC3.F18T10.1

TCCCGCGCCTGGGGGCCCACCCCTGTCCACCGGACCTATAGTGGACCTGCTCCAG

>FGFR3-TACC3.F18T10

GGCGCCTTTCGAGCAGTACTCCCCGGGTGGCCAGGACACCCCCAGCTCCAGCTCCTCAGGGGACGAGGACCTGGATGCAGTGGTAAAGGCGACACAGGAGGAGAAC
CGGGAGCTGAGGAGCAGGTGTGAGGAGCTCCACGGGAAGAACCTGGAACTGGGGAAGATCATGGA

>FGFR3-TACC3.F18T11

GGCGCCTTTCGAGCAGTACTCCCCGGGTGTAAAGGCGACACAGGAGGAGAACCGGGAGCTGAGGAGCAGGTGTGAGGAGCTCCACGGGAAGAACCTGGAACTGGG

>FGFR3-TACC3.TruncatedF17T4

GGACCTGGACCGTGTCCTTACCGTGAATGGAATTCTACAGAAACCAGTGGAGGCTGACACCGACCTCCTGGGGGATGCAAGCCCAGCCTTTG

>FGFR3-TACC3.F17T7

GCACACACGACCTGTACATGATCATGCGGGAGTGCTGGCATGCCGCGCCCTCCCAGAGGCCCACCTTCAAGCAGCTGGTGGAGTACCTGGAGCAGTTTGGAACTTCCT
CGTTTAAGGAGTCGGCCTTGAGGAAGCAGTCCTTATACCTCAAGTTCGACCCCCTCCTGAGGGACAGTCCTGGTAGACC

>FGFR3-TACC3.F17T10

GCACACACGACCTGTACATGATCATGCGGGAGTGCTGGCATGCCGCGCCCTCCCAGAGGCCCACCTTCAAGCAGCTGGTGGAGGACCTGGATGCAGTGGTAAAGGCG
ACACAGGAGGAGAACCGGGAGCTGAGGAGCAGGTGTGAGGAGCTCCACGGGAAGAACCTGGAACTGGGGAAGATCATGGA

>FGFR3-TACC3.F17T11.1

GCACACACGACCTGTACATGATCATGCGGGAGTGCTGGCATGCCGCGCCCTCCCAGAGGCCCACCTTCAAGCAGCTGGTGGAGGACCTCCACGGGAAGAACCTGGAAC

>FGFR3-TACC3.F17T11.2

GCACACACGACCTGTACATGATCATGCGGGAGTGCTGGCATGCCGCGCCCTCCCAGAGGCCCACCTTCAAGCAGCAGGTGTGAGGAGCTCCACGGGAAGAACCTGGAA

>FGFR3-TACC3.F17intron17T4.1

GGTGCCACCCGCCTATGCCCCTCCCCCTGCCGTCCCCGGCCATCCTGCCCCCCAGAGTGCTGAGGTGTGGGGCGGGCCTTCTGGCCCAGGTGCCCTGGCTGACCTGGAC

>FGFR3-TACC3.F17Intron17T9

GGTGCCACCCGCCTATGCCCCTCCCCCTGCCGTCCCCGGCCATCCCTCAGGACGTCCGCGGGAAGCCAAGCTTGTGGAGTTCGATTTCTTGGGAGCACTGGACATTC

>FGFR3-TACC3.F17T14

GGACCTGGACCGTGTCCTTACCGTGACGTCCACCGACAACGAAGAGTCACTGAAGAAGTGCGTGGAGGATTACCTGGCAAGGATCACCCAGGAGGGCCAGAGGTACC

>FGFR3-TACC3.F18T11del5

GGGAAGAACCTGGAACTGGGGAAGATCATGGA

>FGFR3-TACC3.F18T1

GGCGCCTTTCGAGCAGTACTCCCCGGGTGGCCAGGACACCCCCAGCTCCAGCTCCTCAGGGGACGACTCCGGAGGTCCTGGGAGGGTCAGTCTGGCCCGCCTGCCTG
CTGACTTGGGTGTGGCCTGAGCAGGTAAAGGCGACACAGGAGGAGAACCGGGAGCTGAGGAGCAGGTGTGAGGAGCTCCACGGGAAGAACCTGGAACTGGGGAA

>FGFR3-TACC3.F17ins1T10

GCACACACGACCTGTACATGATCATGCGGGAGTGCTGGCATGCCGCGCCCTCCCAGAGGCCCACCTTCAAGCAGCTGGTGGAAGGACCTGGATGCAGTGGTAAAGGC
GACACAGGAGGAGAACCGGGAGCTGAGGAGCAGGTGTGAGGAGCTCCACGGGAAGAACCTGGAACTGGGGAAGATCATGGA

>FGFR3-JAKMIP1.F17J4

AGACCCAGAAGCTGCTTCTGCAGAAAGAGGCTTTGGATGAGCAGC

>FGFR3-TACC3.F17T14.1

AGGCGGAAGCGTTGGCCCTCCAGGCCAGCCTGAGGAAGGAGCAGA

>FGFR3-FBXO28.F17F4

CTTCAAGAATTAAGGGATATATCCTCTATGGCAATGGAGTACTTTGAT

>SLC45A3-ERG.S1E4.COSF1138

CTGGCTCCGGGTGACAGCCGCGCGCCTCGGCCAGGAAGCCTTATCAGTTGTGAGTGAGGACCAGTCGTTGTTTGAGTGTGCCTACG

>TMPRSS2-ERG.T1E2.COSF23.1

GAAAGCCGTGTTGACCAAAAGCAAGACAAATGACTCAC

>TMPRSS2-ERG.T1E3.COSF24

GGGCTGGCTTACTGAAGGACATGATTCAGACTGTC

>TMPRSS2-ERG.T1E4.COSF38

GTAGGCGCGAGCTAAGCAGGAGGCGGAGGCGGAGGCGGAGGGCGAGGGGCGGGGAGCGCCGCCTGGAGCGCGGCAGGAAGCCTTATCAGTTGTGAGTGAGGACC

>TMPRSS2-ERG.T1E5.COSF26

GTAGGCGCGAGCTAAGCAGGAGGCGGAGGCGGAGGCGGAGGGCGAGGGGCGGGGAGCGCCGCCTGGAGCGCGGCAGGAACTCTCCTGATGAATGCAGTGTGGCC

>TMPRSS2-ERG.T1E6.COSF36

GCGGCAGTGGCTGGAGTGGGCGGTGAAAGAATATGGCCTTCCAGA

>TMPRSS2-ERG.T1EIIIc_4

CAAGAATTGCCCCTCCAAGATCTCATTGCTGTTTCTGG

>TMPRSS2-ERG.T2E2.COSF27.1

CAGGTCATATTGAACATTCCAGATACCTATCATTACTCGATGCTGTTGATAACAGCAAGATGGCTTTGAACTCAGTTATTCCAGGATCTTTGGAGACCCGAGGAAAGCCG

>TMPRSS2-ERG.T2E4.COSF28

CAGGTCATATTGAACATTCCAGATACCTATCATTACTCGATGCTGTTGATAACAGCAAGATGGCTTTGAACTCAGAAGCCTTATCAGTTGTGAGTGAGGACCAGTCGTTG

>TMPRSS2-ERG.T2EIIIc_4

CAGGTCATATTGAACATTCCAGATACCTATCATTACTCGATGCTGTTGATAACAGCAAGATGGCTTTGAACTCATTAAACTCCATTGATGATGCACAGTTGACAAGAATTG

>TMPRSS2-ERG.T2E5.COSF29

CAGGTCATATTGAACATTCCAGATACCTATCATTACTCGATGCTGTTGATAACAGCAAGATGGCTTTGAACTCAGAACTCTCCTGATGAATGCAGTGTGGCCAAAGGCGG

>TMPRSS2-ERG.T3E4.COSF30.1

CTGACGCAGGCTTCCAACCCCGTCGTCTGCACGCAGCCCAAATCCCCATCCGGGACAGTGTGCACCTCAAGAAGCCTTATCAGTTGTGAGTGAGGACCAGTCGTTGTTTG

>TMPRSS2-ERG.T4E4.COSF18

CTGTGCATCACCTTGACCCTGGGGACCTTCCTCGTGGGAGCTGCGCTGGCCGCTGGCCTACTCTGGAAGTTCAGAAGCCTTATCAGTTGTGAGTGAGGACCAGTCGTTG

>TMPRSS2-ERG.T4E5.COSF17

CTGTGCATCACCTTGACCCTGGGGACCTTCCTCGTGGGAGCTGCGCTGGCCGCTGGCCTACTCTGGAAGTTCAGAACTCTCCTGATGAATGCAGTGTGGCCAAAGGCGG

>TMPRSS2-ERG.T5E4.COSF16

GTACCTGCATCAACCCCTCTAACTGGTGTGATGGCGTGTCACACTGCCCCGGCGGGGAGGACGAGAATCGGTGTGGAAGCCTTATCAGTTGTGAGTGAGGACCAGTCG

>TMPRSS2-ERG.T5E5

GTACCTGCATCAACCCCTCTAACTGGTGTGATGGCGTGTCACACTGCCCCGGCGGGGAGGACGAGAATCGGTGTGGAACTCTCCTGATGAATGCAGTGTGGCCAAAGG

>TMPRSS2-ETV1.T1E5

CAAGAAAGAACCCCACAGTCCATGTTCAGAAATCAG

>TMPRSS2-ETV1.T2E5

CAGGTCATATTGAACATTCCAGATACCTATCATTACTCGATGCTGTTGATAACAGCAAGATGGCTTTGAACTCATGGCTTTTCATGGCCTGCCACTGAAAATCAAGAAAG

>TMPRSS2-ETV1.T1E4

GTAGGCGCGAGCTAAGCAGGAGGCGGAGGCGGAGGCGGAGGGCGAGGGGCGGGGAGCGCCGCCTGGAGCGCGGCAGCTCAGGTACCTGACAATGATGAGCAGTTT

>TMPRSS2-ETV1.T1bE4

CAGGTCATATTGAACATTCCAGATACCTATCATTACTCGATGCTGTTGATAACAGCAAGATGGCTTTGAACTCACTCAGGTACCTGACAATGATGAGCAGTTTGTACCAG

>TMPRSS2-ETV4.T1bE3

CTTTGATAAATAAGTTTGTAAGAGGAGCCTCAGCATCGTAAAGAGCTTTTCTCCCCGCTTCTCGCAGAAATCGCCCGGAAATGGGAGCTTGCGCGAAGCGCTGATCGGC

>TMPRSS2-ETV5.T1E2.EU314929

GTAGGCGCGAGCTAAGCAGGAGGCGGAGGCGGAGGCGGAGGGCGAGGGGCGGGGAGCGCCGCCTGGAGCGCGGCAGGAGGATCCCTTTTCCCCCAGAAATTACTC

>TMPRSS2-ETV5.T3E2.EU314930.1

CTGACGCAGGCTTCCAACCCCGTCGTCTGCACGCAGCCCAAATCCCCATCCGGGACAGTGTGCACCTCAAGAGGATCCCTTTTCCCCCAGAAATTACTCAATGCTGAAAC

>TMPRSS2-ETV5.T1bE2

AGGACTTCCCCACCTTTACTGAGTGCTTTTGAAGAAGTAGGACCTGCCCTGCTCCGCTACAGAACTACTGGACAGGAGGATCCCTTTTCCCCCAGAAATTACTCAATGCTG

>TPM3-NTRK1.T7N10.COSF1329

CAGAGACCCGTGCTGAGTTTGCTGAGAGATCGGTAGCCAAGCTGGAAAAGACAATTGATGACCTGGAAGACACTAACAGCACATCTGGAGACCCGGTGGAGAAGAAG

>TPR-NTRK1.T21N10.COSF1326

CACCTCAGTAATATGGAAGTCCAAGTTGCTTCTCAGTCTTCACAGAGAACTGGTAAAGACACTAACAGCACATCTGGAGACCCGGTGGAGAAGAAGGACGAAACACCTT

>TPR-NTRK1.T6N12.COSF1324

TGTTAAGTATCGAGAAAAACGCTTGGAGCAAGAAAAGGAATTGCTACATAGGCTCCAAGGCCACATCATCGAGAACCCACAATACT

>MPRIP-NTRK1.M14N12

CCGTCCACCTGTCTTCTGAAGATGGGGGTGACCGGCTCTCCACACACGAGCTGACCTCTCTGCTCGAGAAGGAGGCCCGGCTGTGCTGGCTCCAGAGGATGGGCTGGC

>MPRIP-NTRK1.M18N12

CAGTGCCAGCGTGAGAACCAGGAGCTCAATGCCCACAACCAGGCCCGGCTGTGCTGGCTCCAGAGGATGGGCTGGCCATGTCCCTGCATTTCATGACA

>MPRIP-NTRK1.M21N12

AGTCCCCTGACAGTGCCACGGTGTCCGGATATGGCCCGGCTGTGCTGGCTCCAGAGGATGGGCTGGCCATGTCCCTGCATTTCATGACA

>SQSTM1-NTRK1.S5N10

TGGAGAAGAAGGACGAAACACCTTTTGGGGTCTCG

>SSBP2-NTRK1.S12N12

TCCTCAGCATCTCCTGGGAATTATGTAGGTCCTCCAGGAGGTGGAGGGCCACCAGGAACACCCATCATGCCTAGTCCAGCAGGCCCGGCTGTGCTGGCTCCAGAGGATG

>TFG-NTRK1.T6N10

CGGTGGAGAAGAAGGACGAAACACCTTTTGGGGTCTCG

>BCAN-NTRK1.B13N11

CAAGAGAACGGTCGTTGGGAGGCCCCCCAGATCTCCTGTGTGCCCAGAAGACCTGTCTCGGTGGCTGTGGGCCTGGCCGTCTTTGCCTGCCTCTTCCTTTCTACGCTGCT

>LMNA-NTRK1.L2N11

GAGGGCGAGCTGCATGATCTGCGGGGCCAGGTGGCCAAGGTCTCGGTGGCTGTGGGCCTGGCCGTCTTTGCCTGCCTCTTCCTTTCTACGCTGCTCCTTGTGCTCAACA

>NFASC-NTRK1.N20N10

GATCCGAGTCCAGGCTGAAAATGACTTCGGGAAGGGCCCTGAGCCAGAGTCCGTCATCGGTTACTCCGGAGAAGATTACACTAACAGCACATCTGGAGACCCGGTGGA

>RNF213-NTRK1.R15N12

AAAGGTGGCTCCGAGAAGTTTTTACAAAGAACATGCTCACATCTTCAGGTGCCTCATTCACATACGTCAAGGAAATTGAGGCCCGGCTGTGCTGGCTCCAGAGGATGGG

>IRF2BP2-NTRK1.I1N10.1

GGCCCTTCGAGAGCAAGTTTAAGAAGGAGCCGGCCCTGACTGCAGACACTAACAGCACATCTGGAGACCCGGTGGAGAAGAAGGACGAAACACCTTTTGGGGTCTCG

>TPR-NTRK1.T6N12.1
TGTTAAGTATCGAGAAAAACGCTTGGAGCAAGAAAAGGAATTGCTACATAGTCAGAATACATGGCTGAATACAGAGTTGAAAACCAAAACTGATGAACTTCTTTCATG
ACATTGGGTGGCAGCTCCCTGTCCCCCACCGAGGGCAAAGGCTCTGGGCTCCAAGGCCACATCATCGAGAACCCACAATACT

>CD74-NTRK1.C7N10

ACTGGAGGACCCGTCTTCTGGGCTGGGTGTGACCAAGCAGGATCTGGGCCCAGACACTAACAGCACATCTGGAGACCCGGTGGAGAAGAAGGACGAAACACCTTTTGG

>SQSTM1-NTRK1.S2N10

CCCGGTGGAGAAGAAGGACGAAACACCTTTTGGGGTCTCG

>TP53-NTRK1.T10N9

GAGCTGAATGAGGCCTTGGAACTCAAGGATGCCCAGGCTGGGAAGGAGCCAGGGGGGAGCAGGGCTCACTCCAGTCCCGGCCAGTGTGCAGCTGCACACGGCGGTG

>LMNA-NTRK1.L10N12

CTGGTGCGCTCAGTGACTGTGGTTGAGGACGACGAGGATGAGGATGGAGATGACCTGCTCCATCACCACCACGCCCGGCTGTGCTGGCTCCAGAGGATGGGCTGGCC

>RABGAP1L-NTRK1.R14N16

GCCGCCCGAGAGCATCCTGTACCGTAAGTTCACCACCGAGA

>CHTOP-NTRK1.C5N11

TTCCTTTCTACGCTGCTCCTTGTGCTCAACAAATGTGGA

>TP53-NTRK1.T8N9

CAGAGGAAGAGAATCTCCGCAAGAAAGGGGAGCCTCACCACGAGCTGCCCCCAGGGAGCACTAAGCGAGTCCCGGCCAGTGTGCAGCTGCACACGGCGGTGGAGATG

>TP53-NTRK1.T9N9

CTCCTCTCCCCAGCCAAAGAAGAAACCACTGGATGGAGAATATTTCACCCTTCAGTCCCGGCCAGTGTGCAGCTGCACACGGCGGTGGAGATGCACCACTGGTG

>PPL-NTRK1.P12N13

GGGGAGGGCGCCTTTGGGAAGGTCTTCCTTGCTGAGTGCCAC

>TFG-NTRK1.T6N14

CCTCCTCAGCAGCTCACCCACCAGGCGTTCAGCCACAGCAGCCACCATATACAGGAGCTCAGACTCAAGCAGGTCAGATTGAAGGCACTGAAGGAGGCGTCCGAGAG
TGCTCGGCAGGACTTCCAGCGTGAGGCTGAGCTGCTCACCATGCTGCAGCACCAGCACATCGTG

>TP53-NTRK1.T11N9

TCCCCTCCTTCTCCCTTTTTATATCCCATTTTTATATCGATCTCTTATTTTACAATAAAACTTTGCTGCCACCTGTGTGTCTGAGGGGTGTCCCGGCCAGTGTGCAGCTGCAC

>ARHGEF2-NTRK1.A21N10

GTGGAGAAGAAGGACGAAACACCTTTTGGGGTCTCG

>EPHB2-NTRK1.E3N9

CGCTGCATGTGCAAAGCAGGCTTCGAGGCCGTTGAGAATGGCACCGTCTGCCGAGTCCCGGCCAGTGTGCAGCTGCACACGGCGGTGGAGATGCACCACTGGTG

>CHTOP-NTRK1.C5N10

CGTGGAGGTCCTGGGAGAGGGGGCCTAGGGCGTGGAGCTATGGGTCGTGGCGGAATCGGTGGTAGAGACACTAACAGCACATCTGGAGACCCGGTGGAGAAGAAG

>LMNA-NTRK1.L10N11

CTCTTCCTTTCTACGCTGCTCCTTGTGCTCAACAAATGTGGA

>MRPL24-NTRK1.M1N9

AGGGCCCATGTGCTGAAAATCCGAAGTGCCGCGGAAAGTGGAGTCTCCTTCTCGCCGGTGGACACTAACAGCACATCTGGAGACCCGGTGGAGAAGAAGGACGAAAC

>TPR-NTRK1.T21N9

CACCTCAGTAATATGGAAGTCCAAGTTGCTTCTCAGTCTTCACAGAGAACTGGTAAAGTCTCCTTCTCGCCGGTGGACACTAACAGCACATCTGGAGACCCGGTGGAGA

>TPM3-NTRK1.T7N7.ins

CAGAGACCCGTGCTGAGTTTGCTGAGAGATCGGTAGCCAAGCTGGAAAAGACAATTGATGACCTGGAAGATGGAGTCTCTCTCTGTCACCCAGGCTGGATGGAGTGC
AGTGGCAGTGTGTGTCAAGGCTCACCCCTCCTGCCCTGTGTCCCTACAGACACTAACAGCACATCTGGAGACCCGGTGGAGAAGAAGGACGAAACACCTTTTGGGGTC

>EPS15-NTRK1.E21N9

GAGACCCGGTGGAGAAGAAGGACGAAACACCTTTTGGGGTCTCG

>CEL-NTRK1.C7N7.2

GGATGGCCCAGTGTCTGAAGGTTACTGATCCCCGAGCCCTGACGCTGGGCCATCCCTGGGGCTGACCCTGGCCAATGTCACCAGTGACCTCAACAGGAAGAACGTGAC

>PPL-NTRK1.P22N11

TTTGCCTGCCTCTTCCTTTCTACGCTGCTCCTTGTGCTCAACAAATGTGGA

>PPL-NTRK1.P22N10

TGGAGAAGAAGGACGAAACACCTTTTGGGGTCTCG

>TPR-NTRK1.T16int9N10

GTTGCCATTCCATTACATGCTTCAAGCTTAGATGATGTTTCTCTTGCATCAACTCCAAAACGTCCAAGTACATCACAGACTGTTTCCACTCCTGCTCCATGTGTCCCTACAG
ACACTAACAGCACATCTGGAGACCCGGTGGAGAAGAAGGACGAAACACCTTTTGGGGTCTCG

>EPS15-NTRK1.E21N9.1

ACATCGGTAGAAACGTTGAAGCACAATGATCCTTTTGCTCCTGGTGGAACAGTTGTTGCAGCAAGCGATTCAGTCCCGGCCAGTGTGCAGCTGCACACGGCGGTGGAG

>LMNA-NTRK1.L11N11

GGTCACCCGCTCCTACCTCCTGGGCAACTCCAGCCCCCGAACCCAGGTCTCGGTGGCTGTGGGCCTGGCCGTCTTTGCCTGCCTCTTCCTTTCTACGCTGCTCCTTGTGCT

>TPM3-NTRK1.T7N12

CAGAGACCCGTGCTGAGTTTGCTGAGAGATCGGTAGCCAAGCTGGAAAAGACAATTGATGACCTGGAAGGCCCGGCTGTGCTGGCTCCAGAGGATGGGCTGGCCATG

>LMNA-NTRK1.L5N10

CGCATCGACAGCCTCTCTGCCCAGCTCAGCCAGCTCCAGAAGCAGACACTAACAGCACATCTGGAGACCCGGTGGAGAAGAAGGACGAAACACCTTTTGGGGTCTCG

>LMNA-NTRK1.L6N12

GGCTCCAGAGGATGGGCTGGCCATGTCCCTGCATTTCATGACA

>MRPL24-NTRK1.M1N9.1

AGGGCCCATGTGCTGAAAATCCGAAGTGCCGCGGAAAGTGGAGTCCCGGCCAGTGTGCAGCTGCACACGGCGGTGGAGATGCACCACTGGTG

>NTRK1-NTRK1.N6N8

GGCCAATGTCACCAGTGACCTCAACAGGAAGAACGTGACGT

>ETV6-PDGFRA.E6P12

GGACATGAATATATTTATGTGGACCCGATGCAGC

>SCAF11-PDGFRA.S1P2

CGGAGTGACTGGGACCTGGGAACCTACTGTGGGGCCGCGGCCGGACCGAGCGCCTCGACCTCGGTCTGAGTTTCCCAGAGCTATGGGGACTTCCCATCCGGCGTTCCT

>BCR-PDGFRA.B1P13

GCAGATCTGGCCCAACGATGGCGAGGGCGCCTTCCATGGAGACGCAGGTCGGGTCTTGGGGTCTGGAGCGTTTGGGAAGGTGGTTGAAGGAA

>FIP1L1-PDGFRA.F11P12del45

TGCCTCCAGAAAAGCCAATTCAAGCGTTGGGAAGTGGCAGGATCGATATGGGAGGGCCGAATCACCTGATCTAAGCCAGATGGACATGAATATATTTATGTGGACCCG

>FIP1L1-PDGFRA.F13ins11P12del99

GCACTGCTCCACCTCTGATTCCACCACCGGGAGACTGCTACAGATGGGAGTTTCCAAGAGATGGACTAGTGCTTGGTCGGGTCTTGGGGTCTGGAGCGTTTGGGAAGG

>FIP1L1-PDGFRA.F10P12del47

AGCTGAGTTTACTTCTCCTCCTTCTTTGTTCAAGACTGGGCTTCCACCGAGCAGAGATGGACATGAATATATTTATGTGGACCCGATGCAGC

>FIP1L1-PDGFRA.F12P12del84

GGTCTTGGGGTCTGGAGCGTTTGGGAAGGTGGTTGAAGGAA

>FIP1L1-PDGFRA.F13P12del75

GGGGTCTGGAGCGTTTGGGAAGGTGGTTGAAGGAA

>FIP1L1-PDGFRA.F16ins16P12del71

GTGATCACAGTCCTACACCAAGTGTTTTCAACAGTTTGAATCCATTCAGAATTAATGGTCCAGATGTGGACCCGATGCAGCTGCCTTATGACTCAAGATGGGAGTTTCCA
AGAGATGGACTAGTGCTTGGTCGGGTCTTGGGGTCTGGAGCGTTTGGGAAGGTGGTTGAAGGAA

>FIP1L1-PDGFRA.F11P12del26

TGCCTCCAGAAAAGCCAATTCAAGCGTTGGGAAGTGGCAGGATCGATATGGGAGGGCCGAATCACCTGATCTAAGGGTCATTGAATCAATCAGCCCAGATGGACATGA

>FIP1L1-PDGFRA.F11P12del77

TGCCTCCAGAAAAGCCAATTCAAGCGTTGGGAAGTGGCAGGATCGATATGGGAGGGCCGAATCACCTGATCTAAGGATGCAGCTGCCTTATGACTCAAGATGGGAGT
TTCCAAGAGATGGACTAGTGCTTGGTCGGGTCTTGGGGTCTGGAGCGTTTGGGAAGGTGGTTGAAGGAA

>FIP1L1-PDGFRA.F9P12del38

GCCGAAGACTGTACTATGGAAGTTACACCAGGTGCAGAGATCCAAGATGGCAGATTCAATCTTTTTAAGAATCAGCCCAGATGGACATGAATATATTTATGTGGACCCG

>FIP1L1-PDGFRA.F13P12del71

GCACTGCTCCACCTCTGATTCCACCACCGGGGACCCGATGCAGCTGCCTTATGACTCAAGATGGGAGTTTCCAAGAGATGGACTAGTGCTTGGTCGGGTCTTGGGGTCT

>FIP1L1-PDGFRA.F11P12del77.1

TGCCTCCAGAAAAGCCAATTCAAGCGTTGGGAAGTGGCAGGATCGATATGGGAGGGCCGAATCACCTGATCTAAGGCAGCTGCCTTATGACTCAAGATGGGAGTTTC
CAAGAGATGGACTAGTGCTTGGTCGGGTCTTGGGGTCTGGAGCGTTTGGGAAGGTGGTTGAAGGAA

>FIP1L1-PDGFRA.F10int10P12del22

GACATGAATATATTTATGTGGACCCGATGCAGC

>FIP1L1-PDGFRA.F10int10P12del67

AGCTGAGTTTACTTCTCCTCCTTCTTTGTTCAAGACTGGGCTTCCACCGAGCAGCCATTTTCAAAGGCACTCTGATTATGTGGACCCGATGCAGCTGCCTTATGACTCAAG
ATGGGAGTTTCCAAGAGATGGACTAGTGCTTGGTCGGGTCTTGGGGTCTGGAGCGTTTGGGAAGGTGGTTGAAGGAA

>FIP1L1-PDGFRA.F10int10P12del106

CCACCGAGCAGAATGTATTCCAGTATCAAACTGCAGATCCCAAAAGTGCAAAAACCGAGTTTCCAAGAGATGGACTAGTGCTTGGTCGGGTCTTGGGGTCTGGAGCGTT

>FIP1L1-PDGFRA.F12P12del107

ACTATCAGCCGAGTAGAAGGCAGGCGACGGGCAAATGAGAACAGCAACATACAGGTTTCCAAGAGATGGACTAGTGCTTGGTCGGGTCTTGGGGTCTGGAGCGTTTG

>FIP1L1-PDGFRA.F11P12del24

TGCCTCCAGAAAAGCCAATTCAAGCGTTGGGAAGTGGCAGGATCGATATGGGAGGGCCGAATCACCTGATCTAAGAGGGTCATTGAATCAATCAGCCCAGATGGACAT

>FIP1L1-PDGFRA.F13insP12del91

GCACTGCTCCACCTCTGATTCCACCACCGGTAGATTATTTTTGTCTGTCCTATGATGACTCAAGATGGGAGTTTCCAAGAGATGGACTAGTGCTTGGTCGGGTCTTGGGG

>STRN-PDGFRA.S6P12.1

GCAGCTGCAGATTTCAGTGATGAAGATGAAGATGATGATGTTGATGGAAGAGAGAAAAGCGTCATTGATACTTCAACACTGCCTTATGACTCAAGATGGGAGTTTCCA
AGAGATGGACTAGTGCTTGGTCGGGTCTTGGGGTCTGGAGCGTTTGGGAAGGTGGTTGAAGGAA

>STRN-PDGFRA.S6P12

GGTCTTGGGGTCTGGAGCGTTTGGGAAGGTGGTTGAAGGAA

>KDR-PDGFRA.K13ins35P10

TTTAATGTCGCTGAACATCATCACGGAGATCCACTCCCGAGACAGGA

>BCR-PDGFRA.B7ins24P12

CTCTCTGGAAACTCTGCTCTACAAGCCTGTGGACCGTGTGACGAGGAGCACGCTGGTCCTCCATGTAAGTCACAGCGCCCCTCTGGACCAGCTGCCTTATGACTCAAGA
TGGGAGTTTCCAAGAGATGGACTAGTGCTTGGTCGGGTCTTGGGGTCTGGAGCGTTTGGGAAGGTGGTTGAAGGAA

>BCR-PDGFRA.B12ins12P12

ATGGACATGAATATATTTATGTGGACCCGATGCAGC

>TNKS2-PDGFRA.T25P12

GGAGCTGGCATTTATTTTGCTGAAAACTCTTCCAAAAGCAATCAATATGTATATGGAATTGGAGGAGGTACTGGGTGTCCAGTTCACAAAGACAGATCTTGTTACATTT
GCCACAGAAACCGAGGTATGAAATTCGCTGGAGGGTCATTGAATCAATCAGCCCAGATGGACATGAATATATTTATGTGGACCCGATGCAGC

>CDK5RAP2-PDGFRA.C13ins40P12

AGAAAGTACCAATCAGAAGGACGTGTTGCTTCAGGCCTGGAGCCCTCCCTTCTCAAAGAGAACCCTGCGGGCAACTTATGACTCAAGATGGGAGTTTCCAAGAGATGG
ACTAGTGCTTGGTCGGGTCTTGGGGTCTGGAGCGTTTGGGAAGGTGGTTGAAGGAA

>FOXP1-PDGFRA.F16P12
CAGATATTGCGCAGAACCAAGAATTTTATAAGAACGCAGAAGTTAGACCACCATTTACATATGCATCTTTAATTAGGCAGCTGCCTTATGACTCAAGATGGGAGTTTCC
AAGAGATGGACTAGTGCTTGGTCGGGTCTTGGGGTCTGGAGCGTTTGGGAAGGTGGTTGAAGGAA

>KIF5B-PDGFRA.K23P12

GCGCTGCTCAGAAGCAAAAAATCTCCTTTCTTGAAAATGAAATTCGCTGGAGGGTCATTGAATCAATCAGCCCAGATGGACATGAATATATTTATGTGGACCCGATGCA[illegible]

>DIP2C-PDGFRA.D1P10

CAACAATGTCTCAAACATCATCACGGAGATCCACT

>DIP2C-PDGFRA.D1P11

CAGCCTGGAGGGCATGGCGCTGCCCCTGGAGGTGCGGGCGCGCCTGGCCGAGCTGGAGCTGGAGCTGTCGGAAGCCCTGCGTTCTGAACTCACGGTGGCTGCTGCAG

>CAPRIN1-PDGFRB.C7P11

GGCCCTGGTGGTGCTCACCATCATCTCCCTTATCATCCTCATCATGCTTTGGC

>CCDC6-PDGFRB.C7P11

CTTACACACCTTCTCCGAGTTCAAGCAGGCCTATATCACCTGCCTTGCCCTTTAAGGTGGTGGTGATCTCAGCCATCCTGGCCCTGGTGGTGCTCACCATCATCTCCCTTAT

>CEP85L-PDGFRB.C11P12

GGTGATTGAGTCTGTGAGCTCTGACGGCCATGAGTACATCTACGTGGACCC

>ERC1-PDGFRB.E15P10

CGCTGCTGGGGAACAGTTCCGAAGAGGAGAGCCAGC

>ERC1-PDGFRB.E15P11
GCAGTCTCTGGCAGAAAAGGAAACTCACTTGACTAATCTTCGGGCAGAGAGAAGGAAACACTTAGAGGAAGTTCTGGAGATGAACCTTGCCCTTTAAGGTGGTGGTG
ATCTCAGCCATCCTGGCCCTGGTGGTGCTCACCATCATCTCCCTTATCATCCTCATCATGCTTTGGC

>ETV6-PDGFRB.E4P11

GCCCTGGTGGTGCTCACCATCATCTCCCTTATCATCCTCATCATGCTTTGGC

>ETV6-PDGFRB.E7P10

CCTGCGCCACTACTACAAACTAAACATTATCAGGAAGGAGCCAGGACAAAGGCTTTTGTTCAGGTGTCCACGTGAGCTGCCGCCCACGCTGCTGGGGAACAGTTCCGAA

>GIT2-PDGFRB.G12P11

CTGGCCCTGGTGGTGCTCACCATCATCTCCCTTATCATCCTCATCATGCTTTGGC

>GOLGA4-PDGFRB.G10P11

GGCCCTGGTGGTGCTCACCATCATCTCCCTTATCATCCTCATCATGCTTTGGC

>HIP1-PDGFRB.H30P11

TGGTGGTGCTCACCATCATCTCCCTTATCATCCTCATCATGCTTTGGC

>PDE4DIP-PDGFRB.P16P11

ACTTCATTGACTGCCAAAGAGGATGTCAGCATACCCAGATCCACATTAGCCTTGCCCTTTAAGGTGGTGGTGATCTCAGCCATCCTGGCCCTGGTGGTGCTCACCATCAT[illegible]

>RABEP1-PDGFRB.R14P11
CAGATCCAAGCAGAACAGTGTTTAAAAGAAAATCTTGAAGAAACTCTGCAACTAGAAATAGAAAACTGCAAGGAGGAAATAGCCTTGCCCTTTAAGGTGGTGGTGATC
TCAGCCATCCTGGCCCTGGTGGTGCTCACCATCATCTCCCTTATCATCCTCATCATGCTTTGGC

>SPECC1-PDGFRB.S3P11
TGAAAGAAACCATATTTGAATTGGAAGATCAGGTGGAACAGCACCGGGCTGTCAAGTTACACAATAATCAACTCATCAGTGAGCTAGAAACCTTGCCCTTTAAGGTGG
TGGTGATCTCAGCCATCCTGGCCCTGGTGGTGCTCACCATCATCTCCCTTATCATCCTCATCATGCTTTGGC

>TP53BP1-PDGFRB.T23P11

CAAGCGAGGTCGCAAGTCTGCCACAGTAAAACCTGCCTTGCCCTTTAAGGTGGTGGTGATCTCAGCCATCCTGGCCCTGGTGGTGCTCACCATCATCTCCCTTATCATCCT

>TRIP11-PDGFRB.T16P11

CCTGGTGGTGCTCACCATCATCTCCCTTATCATCCTCATCATGCTTTGGC

>WDR48-PDGFRB.W9P12

CTGCAATTTGGGTTGCAACAACTAAGTCTACAGTAAATAAATGGAAGCCACGTTACGAGATCCGATGGAAGGTGATTGAGTCTGTGAGCTCTGACGGCCATGAGTACAT

>NIN-PDGFRB.N30P12

GAAGGTGATTGAGTCTGTGAGCTCTGACGGCCATGAGTACATCTACGTGGACCC

>TPM3-PDGFRB.T7P11

CCCTGGTGGTGCTCACCATCATCTCCCTTATCATCCTCATCATGCTTTGGC

>DTD1-PDGFRB.D4P12

TCAGAATGATGGGCCTGTGACCATAGAGCTGGAATCGCCAGCTCCCGGCACTGCTACCTCTGACCCAAAGCAGGTGATTGAGTCTGTGAGCTCTGACGGCCATGAGTAC

>BIN2-PDGFRB.B9P12

GAGCAAACTGGAGAAGCAACATTCCAATAAAGTCTTTGTGGTGAAGGGACTGTCAAGAGTTTCTTTAGGGTCTATACTTTCTGTGAGCTCTGTGAGCTCTGACGGCCAT

>NDE1-PDGFRB.N6P11
TCGAAAGAAATGCCTTCCTGGAAAGTGAACTTGATGAAAAAGAGAATCTCCTGGAATCTGTTCAGAGACTGAAGGATGAAGCCAGAGCCTTGCCCTTTAAGGTGGTGG
TGATCTCAGCCATCCTGGCCCTGGTGGTGCTCACCATCATCTCCCTTATCATCCTCATCATGCTTTGGC

>PRKG2-PDGFRB.P6P12

CCTCAGAAGTGTATCCTTGCTGAAGAATTTACCTGAAGATAAATTAACCAAGATCATTGACTGCTTGGAAGTGCCTTTGAATGTGTTTGCGAGTCTGTGAGCTCTGACGG

>CCDC88C-PDGFRB.C10P12

GCTGGAGCTGGAGCTGACCCGCTGCAAGGAGAAGCTGCACGACGTGGACTTCTACAAGGCCCGCATGGAGATCCGATGGAAGGTGATTGAGTCTGTGAGCTCTGACG

>CCDC88C-PDGFRB.C25P11

GCTCACCATCATCTCCCTTATCATCCTCATCATGCTTTGGC

>EBF1-PDGFRB.E15P11

TGGTGGTGCTCACCATCATCTCCCTTATCATCCTCATCATGCTTTGGC

>TNIP1-PDGFRB.T14P11
CGCATGAATGAGGAGAAGGAAGAGCTGAAGAAGCAAGTGGAGAAGCTGCAGGCCCAGGTCACCCTGTCAAATGCCCAGCCTTGCCCTTTAAGGTGGTGGTGATCTCA
GCCATCCTGGCCCTGGTGGTGCTCACCATCATCTCCCTTATCATCCTCATCATGCTTTGGC

>SART3-PDGFRB.S15P11

CAGATGAGGATGATGAGAAAGAGTGGGGCGATGATGAAGAAGCCTTGCCCTTTAAGGTGGTGGTGATCTCAGCCATCCTGGCCCTGGTGGTGCTCACCATCATCTCCC

>MYO18A-PDGFRB.M41P10

GGGTCAAGTCCTGGTTGTCAAAAAACAAGGGACCTTCCAAGGCAGCTTCTGATGATGGCAGCTTAAAGAGTTCCAGGTGTCCACGTGAGCTGCCGCCCACGCTGCTGG

>ATF7IP-PDGFRB.A13P11

TGCTCACCATCATCTCCCTTATCATCCTCATCATGCTTTGGC

>EBF1-PDGFRB.E11P11

CTGGCCCTGGTGGTGCTCACCATCATCTCCCTTATCATCCTCATCATGCTTTGGC

>CCDC88C-PDGFRB.C12P11
AGAAGCAGAGCATGAACGAATCTGCCCACCTTGGCTGGGAGCTGGAGCAGCTGTCCAAGAACGCAGACTTGTCAGACGCCTTGCCCTTTAAGGTGGTGGTGATCTCA
GCCATCCTGGCCCTGGTGGTGCTCACCATCATCTCCCTTATCATCCTCATCATGCTTTGGC

>ETV6-PDGFRB.E4P9

CACCATTCTTCCACCCTGGAAACTCTATACACACACAGCCGGAGGTCATACTGCATCAGAACCATGAAGAAGTCCCTGTCCGAGTGCTGGAGCTAAGTGAGAGCCACCC

>PRKG2-PDGFRB.P3P12

GGTGGAATGCATGTATGGGAGAAACTATCAGCAAGGGAGTTACATTATTAAGCAAGGAGAACCAGGAAACCATATCTTTGTGCTGGCAGAGGGTGAGTCTGTGAGCT

>EBF1-PDGFRB.E14P11

GGCCCTGGTGGTGCTCACCATCATCTCCCTTATCATCCTCATCATGCTTTGGC

>ZEB2-PDGFRB.Z9P9

AAGTGAGAGCCACCCTGACAGTGGGGAACAGACAGTC

>KANK1-PDGFRB.K2P9

GTGAGAGCCACCCTGACAGTGGGGAACAGACAGTC

>GOLGB1-PDGFRB.G10P12

CTGTGAGCTCTGACGGCCATGAGTACATCTACGTGGACCC

>MPRIP-PDGFRB.M20P12

GGTGATTGAGTCTGTGAGCTCTGACGGCCATGAGTACATCTACGTGGACCC

>CPSF6-PDGFRB.C5P11

AGATTTCCTGGGCCAGCAGGACCAGGAGGGCCACCCCCACCTTTTCCAGCCTTGCCCTTTAAGGTGGTGGTGATCTCAGCCATCCTGGCCCTGGTGGTGCTCACCATCAT

>ETV6-PDGFRB.E7ins34P12

CCTGCGCCACTACTACAAACTAAACATTATCAGGAAGGAGCCAGGACAAAGGCTTTTGTTCAGTCAGAATAAGTCTACAAGCTGCTGCACTAGAATTAGCTCTGACGGC

>BCR-JAK2.B1J19.COSF752

GCAGATCTGGCCCAACGATGGCGAGGGCGCCTTCCATGGAGACGCAGATTATGAACTATTAACAGAAAATGACATGTTACCAAATATGAGGATAGGTGCCCTGGGGTT

>ETV6-JAK2.E5J12.COSF995

TTCAGAACGAATGGTGTTTCTGATGTACCAACCTCACCAACATTACAGAG

>ETV6-JAK2.E5J19.COSF989
TCATCGGGAAGACCTGGCTTACATGAACCACATCATGGTCTCTGTCTCCCCGCCTGAAGAGCACGCCATGCCCATTGGGAGAATAGCAGATTATGAACTATTAACAGAA
AATGACATGTTACCAAATATGAGGATAGGTGCCCTGGGGTTTTCTGGTGCCTTTGAAGACCGGGATC

>ETV6-JAK2.E4J17.COSF993

CATTGAAAATCCTAAAAATTTAAATTTGGCAACAGACAAATGGAGTTTTGG

>PAX5-JAK2.P5J19.COSF1071
CGGCTCGTCGTACTCCATCAGCGGCATCCTGGGCATCACGTCCCCCAGCGCCGACACCAACAAGCGCAAGAGAGACGAAGATTATGAACTATTAACAGAAAATGACAT
GTTACCAAATATGAGGATAGGTGCCCTGGGGTTTTCTGGTGCCTTTGAAGACCGGGATC

>PCM1-JAK2.P23J12.COSF1001

CGAATGGTGTTTCTGATGTACCAACCTCACCAACATTACAGAG

>PCM1-JAK2.P26J9.COSF1007

CATCAAACTCAGAACTTACTCCTAGTGAGAGCCTTGCTACTACTGATGATGAAATTGAACTTAGCTCATTAAGGGAAGCTTTGTCTTTCGTGTCATTAATTGATGGATATT

>PCM1-JAK2.P36J9.COSF1009

TGGCTGGAAGTCCTGATACTGAATCTCCAGTGTTAGTGAATGACTATGAAATTGAACTTAGCTCATTAAGGGAAGCTTTGTCTTTCGTGTCATTAATTGATGGATATTATA

>PCM1-JAK2.P36J11.COSF998

TGGCTGGAAGTCCTGATACTGAATCTCCAGTGTTAGTGAATGACTATCGAGAAAATGTCATTGAATATAAACACTGTTTGATTACAAAAAATGAGAATGAAGAGTACAA

>SEC31A-JAK2.S22J17.COSF1069
GCTCCACCATCATCTTCAGCTTATGCACTGCCTCCTGGAACAACAGGTACACTGCCTGCTGCCAGTGAGCTGCCTGCGTCCCAAAGAACAGTTCTTCAGGAGAGAATAC
CATGGGTACCACCTGAATGCATTGAAAATCCTAAAAATTTAAATTTGGCAACAGACAAATGGAGTTTTGG

>SSBP2-JAK2.S4J11.COSF1047

ACACTCAAGTGAAGCAAAAGCCTTCCATGATTACCGAGAAAATGTCATTGAATATAAACACTGTTTGATTACAAAAAATGAGAATGAAGAGTACAACCTCAGTGGGACA

>SSBP2-JAK2.S5J11.COSF1045
TCCCAGTCCAGTGCTAGGAAACATTCCCCCAGGAGATGGCATGCCAGTAGGTCCTGTACCACCAGGGTTCTTTCAGCGAGAAAATGTCATTGAATATAAACACTGTTTG
ATTACAAAAAATGAGAATGAAGAGTACAACCTCAGTGGGACAAAGAAGAACTTCAG

>BCR-JAK2.B1J17.COSF987

GCAGATCTGGCCCAACGATGGCGAGGGCGCCTTCCATGGAGACGCAGTTCTTCAGGAGAGAATACCATGGGTACCACCTGAATGCATTGAAAATCCTAAAAATTTAAAT

>ETV6-JAK2.E4J16.COSF994

AAAATATTCTGCTTATCAGAGAAGAAGACAGGAAGACAGGAAATCCTCC

>PCM1-JAK2.P24J17.COSF1003
TGTTCAAGCAAAAGTATTCAGCAGAAAGAATCATGAGCAACTGGAAAAAATAATAAAATGTAATAGGTCTACAGAAATATCTTCAGTTCTTCAGGAGAGAATACCATG
GGTACCACCTGAATGCATTGAAAATCCTAAAAATTTAAATTTGGCAACAGACAAATGGAGTTTTGG

>ETV6-JAK2.E5J17
TCATCGGGAAGACCTGGCTTACATGAACCACATCATGGTCTCTGTCTCCCCGCCTGAAGAGCACGCCATGCCCATTGGGAGAATAGCAGTTCTTCAGGAGAGAATACCA
TGGGTACCACCTGAATGCATTGAAAATCCTAAAAATTTAAATTTGGCAACAGACAAATGGAGTTTTGG

>PCM1-JAK2.P29J13

CAGCTGGACCGGCAAATTAAAGCAATTATGAAAGAAGTCATTCCTTTTTTGAAGAATGAAAGCCTTGGCCAAGGCACTTTTACAAAGATTTTTAAAGGCGTACGAAGAG

>TERF2-JAK2.T8J19
CCAAAGTACCCAAAGGCAAGTGGAACAGCTCTAATGGGGTTGAAGAAAAGGAGACTTGGGTGGAAGAGGATGAACTGTTTCAAGTTCAGGATTATGAACTATTAACA
GAAAATGACATGTTACCAAATATGAGGATAGGTGCCCTGGGGTTTTCTGGTGCCTTTGAAGACCGGGATC

>PCM1-JAK2.P28J11

AGAATGAAGAGTACAACCTCAGTGGGACAAAGAAGAACTTCAG

>PPFIBP1-JAK2.P12J19

ATGAGGATAGGTGCCCTGGGGTTTTCTGGTGCCTTTGAAGACCGGGATC

>STRN3-JAK2.S9J17

ATTTAAATTTGGCAACAGACAAATGGAGTTTTGG

>PCM1-JAK2.P35J11
ACAGGTCCTACAACGTGACTTTAAAAAGACAGCAGAAAGCAAAAATGTCCCATTGGAACGAGAAGCCACTAGTAAAACGAGAAAATGTCATTGAATATAAACACTGTT
TGATTACAAAAAATGAGAATGAAGAGTACAACCTCAGTGGGACAAAGAAGAACTTCAG

>EBF1-JAK2.E14J17

GCATTGAAAATCCTAAAAATTTAAATTTGGCAACAGACAAATGGAGTTTTGG

>TPR-JAK2.T39J17

TCCTAAAAATTTAAATTTGGCAACAGACAAATGGAGTTTTGG

>ATF7IP-JAK2.A13J17

CTAAAAATTTAAATTTGGCAACAGACAAATGGAGTTTTGG

>SPAG9-JAK2.S25J19

ATAGGTGCCCTGGGGTTTTCTGGTGCCTTTGAAGACCGGGATC

>BICD2-JAK2.B7J13

GAGCTGGACCATGAGCAGACCCGGCGTGGCCGTGCCAAAGCCGCCCCGAAGACCAAGCCAGCCACACCGAGCAATGAAAGCCTTGGCCAAGGCACTTTTACAAAGATT

>TPM3-JAK2.T7J17

ATTGAAAATCCTAAAAATTTAAATTTGGCAACAGACAAATGGAGTTTTGG

>SSBP2-JAK2.S8J18

GCATCAGCTTCCTGCACCAAAGTGGGCAGAATTAGCAAACCT

>SSBP2-JAK2.S10J18

CATCAGCTTCCTGCACCAAAGTGGGCAGAATTAGCAAACCT

>PCM1-JAK2.P36J9ins3

TGGCTGGAAGTCCTGATACTGAATCTCCAGTGTTAGTGAATGACTATCGAGAAATTGAACTTAGCTCATTAAGGGAAGCTTTGTCTTTCGTGTCATTAATTGATGGATATT

>PCM1-JAK2.P36J11ins12

TGGCTGGAAGTCCTGATACTGAATCTCCAGTGTTAGTGAATGACTATAAAAATCTCTGTCACTGTTTGATTACAAAAAATGAGAATGAAGAGTACAACCTCAGTGGGACA

>BCR-JAK2.B1J15

AAATTGTATAAATATATTATGGAAACTTGAAGTTGCTAAACAGTTGGCATG

>OFD1-JAK2.O21J13

CAAAGATTTTTAAAGGCGTACGAAGAGAAGTAGGA

>NACC2-NTRK2.N4N13.COSF1448

CATCCGCTCGTCCACCAGCGACCCCAGCCGGAAGCCGCTGGACAGCCGGGTCCTGAACGCTGTGAAATATTATGGAACTGCAGCGAATGACATCGGGGACACCAC

>QKI-NTRK2.Q6N16.COSF1446

ACACCCTATGAGTACCCCTACACATTGGCACCAGCTACATCAATCCTTGAGTATCCTATTGAACCTAGTGGTGTATTAGGCCCAGCCTCCGTTATCAGCAATGATGATGAC

>AFAP1-NTRK2.A14N12

CTCTGAGCAGTCAGCCAAAGAAAGCGGATCCCGCGGCTGTTGTGAAAAGGACGGGTTCGAGTGCAAACCCAAATTATCCTGATGTAATTTATGAAGATTATGGAACTGC

>AGBL4-NTRK2.A6N16

AGCCTCCGTTATCAGCAATGATGATGACTCTGCCAGCCCAC

>SQSTM1-NTRK2.S5N17

CTTCTGGTCCATCGGAGGATCCGAGTGTGAATTTCCTGAAGAACGTTGGGGAGAGTGTGGCAGCTGCCCTTAGCCCTCTGGTTGTTCAGCACATCAAGCGACATAACAT

>TRIM24-NTRK2.T12N15

GCCTCCGTTATCAGCAATGATGATGACTCTGCCAGCCCAC

>VCL-NTRK2.V16N12

TGATGTAATTTATGAAGATTATGGAACTGCAGCGAATGACATCGGGGACACCAC

>DAB2IP-NTRK2.D1N17

GACATAACATTGTTCTGAAAAGGGAGCTAGGCG

>TRIM24-NTRK2.T12N16

CTCAAACAGAACGGTCCAGTCACCAAATTCATCAGTGCCATCTCCAGGCCTTGCAGGCCCAGCCTCCGTTATCAGCAATGATGATGACTCTGCCAGCCCAC

>NAV1-NTRK2.N15N11

CTATCACATTTCTCGAATCTCCAACCTCAGACCACCACTGGT

>SLMAP-NTRK2.S14N16

CGCCCAAATGGATGAGCAAGACCTAAATGAGCCTCTTGCCAAAGTGTCCCTTTTAAAAGGCCCAGCCTCCGTTATCAGCAATGATGATGACTCTGCCAGCCCAC

>ETV6-NTRK3.E4N15.COSF823.2

CACCATTCTTCCACCCTGGAAACTCTATACACACACAGCCGGAGGTCATACTGCATCAGAACCATGAAGAAGATGTGCAGCACATTAAGAGGAGAGACATCGTGCTGAA

>ETV6-NTRK3.E5N15.COSF571.1

TCATCGGGAAGACCTGGCTTACATGAACCACATCATGGTCTCTGTCTCCCCGCCTGAAGAGCACGCCATGCCCATTGGGAGAATAGCAGATGTGCAGCACATTAAGAGG

>BTBD1-NTRK3.B4N14

GAAAAGCACTTTCCTTAATCCGGTTCCCACTGATGACAATTGAGGAATTTGCAGCAGGTCCCGTGGCTGTCATCAGTGGTGAGGAGGACTCAGCCAGCCCACTGCACCA

>COX5A-NTRK3.C1N15

GGATGTGCAGCACATTAAGAGGAGAGACATCGTGCTGAAGCGAGAACT

>ETV6-NTRK3.E4N14.1

CCAGCCCACTGCACCACATCAACCACGGCATCACC

>ETV6-NTRK3.E5N14

GGTGAGGAGGACTCAGCCAGCCCACTGCACCACATCAACCACGGCATCACC

>FAT1-NTRK3.F2N7

ATGATGAGGACGCCAGAAGAGATGGGGAGATCCGATACTCCATTAGAGATGGCTCTGGCGTTGGTGTTTTCAAAATAGGTGAAGAGACAGGCAGTTGGAGCAGAACT

>EML4-NTRK3.E2N14

GAGGACTCAGCCAGCCCACTGCACCACATCAACCACGGCATCACC

>AKAP13-NTRK3.A14N14

AGCCCACTGCACCACATCAACCACGGCATCACC

>LYN-NTRK3.L8N14

GTGGTGAGGAGGACTCAGCCAGCCCACTGCACCACATCAACCACGGCATCACC

>RBPMS-NTRK3.R5N14

GGACTCCAAACCCCAGTACTCCTCTGCCCAACACTGTACCTCAGTTCATTGCCAGAGAGCCATGTCCCGTGGCTGTCATCAGTGGTGAGGAGGACTCAGCCAGCCCACTG

>SRGAP3-RAF1.S12R10.COSF661

CCTCTCTCAGTGTATAGCCATAAACTCTTTAACGGCAGTATGGAAGCATTCATTAAGAGGCCTCGTGGACAGAGAGATTCAAGCTATTATTGGGAAATAGAAGCCAGTG[illegible]

>B4GALT1-RAF1.B1R8.1

ATCAGCCTCACCTTCAGCCCTGTCCAGTAGCCCCAACAATCTGAGCCCAACAGGC

>ESRP1-RAF1.E13R6.COSF826

AGCCCTCCACAGCGTACTACCCAGCAGGCACTCAGCTCTTCATGAATTACACAGCGTACTATCCCAGATTGTTTCCAAATTCCACTATTGGTGATAGTGGAGTCCCAGCAC

>AGGF1-RAF1.A5R8

GAATCAGCCTCACCTTCAGCCCTGTCCAGTAGCCCCAACAATCTGAGCCCAACAGGC

>MPRIP-RAF1.M22R8

CCTCACCTTCAGCCCTGTCCAGTAGCCCCAACAATCTGAGCCCAACAGGC

>PAPD7-RAF1.P11R10

CCAATGCCCAGTGGCAAACCTCAGCCCACCACTTCCAGAACACTGATCATGACAACCAACAATCAGAGGCCTCGTGGACAGAGAGATTCAAGCTATTATTGGGAAATAG[illegible]

>HACL1-RAF1.H16R8

CTTCAGCCCTGTCCAGTAGCCCCAACAATCTGAGCCCAACAGGC

>CNTLN-RAF1.C5R8

TTCAGCCCTGTCCAGTAGCCCCAACAATCTGAGCCCAACAGGC

>PDZRN3-RAF1.P5R8

CCAAATGACTACATTGGAGACATCCATCAGGAGATGGACAGGGAGGAGCTGGAGCTGGAGGATGCAATTCGAAGTCACAGCGAATCAGCCTCACCTTCAGCCCTGTCC[illegible]

>CLCN6-RAF1.C2R8

ACAGGAGGAGGAGGATGAGATTCTTCCAAGGAAAGACTATGAGGATGCAATTCGAAGTCACAGCGAATCAGCCTCACCTTCAGCCCTGTCCAGTAGCCCCAACAATCT[illegible]

>TRAK1-RAF1.T9R8

CCTTCAGCCCTGTCCAGTAGCCCCAACAATCTGAGCCCAACAGGC

>TRIM33-RAF1.T9R10

AGCACCTGTACCAACTACAACAACAACAACACAACAGCATCCTAGACAAGCAGCCCCTCAGATGTTACAACAACAGAGGCCTCGTGGACAGAGAGATTCAAGCTATTAT[illegible]

>SRGAP3-RAF1.S11R8
AGGCCAAGCACGATTTACTCAAGCAGACCCTGGGCGAAGGGGAAAGAGCAGAATGCGGCACCACCAGGGGAAAGAGCAGAATGCGGCACCACCAGGATGCAATTCG
AAGTCACAGCGAATCAGCCTCACCTTCAGCCCTGTCCAGTAGCCCCAACAATCTGAGCCCAACAGGC

>LMNA-RAF1.L10R8

TCAGCCCTGTCCAGTAGCCCCAACAATCTGAGCCCAACAGGC

>GOLGA4-RAF1.G16R8

AAGATGATGATCTAAAACGAACAGCCAAAAGATATGAAGAAATCCTTGATGATGCAATTCGAAGTCACAGCGAATCAGCCTCACCTTCAGCCCTGTCCAGTAGCCCCAA[illegible]

>FYCO1-RAF1.F11R6

AGTCCCAGCACTACCTTCTTTGACTATGCGTCGTATGCGA

>QKI-RAF1.Q3R8

TGTCCAGTAGCCCCAACAATCTGAGCCCAACAGGC

>FGFR3-TACC3.F17T4

AGCCGAGACCCCAACAGCAGAGAGCAAGGAGAGAGCCTT

>BRAF-MACF1.B8M15

CCTCATCAGCTCCCAATGTGCATATAAACACAATAGAACCTGTCAATATTGATATGAAACTGGAGCGAGCAGAGTGGGGCAATGACCTGCCTAGTGTGGAGTTGCAGCT[illegible]

>AR-AR.E4E7.V56

GATGAACTTCGAATGAACTACATCAAGGAACTCGATCG

>AR-AR.E7E8.V14

CATCCTGCTCAAGACGCTTCTACCAGCTCACCAAGCTCCTGGACTCCGTGCAGCCTATCACACCTGATGCCATGTACTTGTGAGAGAGGATGCAGTTTTGTT

>MYB-ESR1.M15E8

TTCGACATGCTGCTGGCTACATCATCTCGGTTCCG

>WHSC1L1-FGFR1.W1F2

TGGAGATGTGGAGCCTTGTCACCAACCTCTAACTGCAG

>BRCA1-BRCA1.B19B21.V20es

GGATGGTACAGCTGTGTGGTGCTTCTGTGGTGAAGGAGC

>BRCA1-BRCA1.B13B15.V14es

ATGCACAGTTGCTCTGGGAGTCTTCAGAATAGA

>BRCA1-BRCA1.B10B16.V11

AATCTGGAATCAGCCTCTTCTCTGATGACCCTGAATCTGATCC

>BRCA1-BRCA1.B11B16

AGACTGCTCAGGGCTATCCTCTCAGAGTGACATTTTAACCACTCAGAGGGAACCCCTTACCTGGAATCTGGAATCAGCCTCTTCTCTGATGACCCTGAATCTGATCC

>MDM4-MDM4.M5M10
CTTCTCCGTGAAAGACCCAAGCCCTCTCTATGATATGCTAAGAAAGAATCTTGTCACTTTAGCCACTGCTACTACAGGTGATTGAAGTGGGAAAAAATGATGACCTGGA
GGACTCTAAGTCCTTAAGTGATGATACCGATGTAGAGGTTACCTCTGAGGATGAGTGGCAGTGTACTGAATGCAA

>BRCA1-BRCA1.B7B9.V8

AACCAGGGATGAAATCAGTTTGGATTCTGCAAAAAAGGCTGC

>BRCA1-BRCA1.B16B18

AGCTTCAACAGAAAGGGTCAACAAAAGAATGTCCATGGTGGTGTCTGGCCTGACCCCAGAAGAATTTATGCTGAGTTTGTGTGTGAACGGACACTGAAATATTTTCTAG[illegible]

>EGFR-GNS.E17G14

GTTTGACCCCCGTCTCATGTTCAGCAATCGCGGCAGTGTCAGG

>ERLIN2-FGFR1.E8F2

CATGGAGATGTGGAGCCTTGTCACCAACCTCTAACTGCAG

>BRAF-AP3B1.B8A23

CCTCATCAGCTCCCAATGTGCATATAAACACAATAGAACCTGTCAATATTGATGTCAGTACTCCTGCATTTGTACCAACGAAAACTCACGTGCTGCTTCATCGAATGAGTG[illegible]

>BRAF-CIITA.B9C6

GGCAGACCTGAAGCACTGGAAGCCAGCTGAGC

>TRIM24-BRAF.T11B2

TGATTAAGTTGACACAGGAACATATAGAGGCCCTATTGGACAAATTTGGTGGG

>BBS9-BRAF.B19B4

CGAGACAGTCTAAAGAAAGCACTGATGATGAGAGGTCTAAT

>SND1-BRAF.S9B2

CAGGAACATATAGAGGCCCTATTGGACAAATTTGGTGGG

>FGFR1-PLAG1.F2P2.COSF1111

TGCTGGTCACAGCCACACTCTGCACCGCTAGGCCGTCCCCGACCTTGCCTGAACAAGGTTGCCTCTTGGTGCTGCCTTGGCCGTATTTGGCACCCAGAATGCTTCATTCTG

>FGFR1-PLAG1.F2P3.COSF1113

TGCTGGTCACAGCCACACTCTGCACCGCTAGGCCGTCCCCGACCTTGCCTGAACAAGATTGGCCAAAATGGGAAGGATTGGATTCCACTCTCTTCCACGAAGAGTCAATG

>FGFR2-CCDC6.F17C2

CACTTTATTCAAGAAAATTCAGGCTTTGCAGAAGGAGAAAGAAACCC

>LRP1.ENCTRL.E57E58

AGCATGATCCGAAGGATGCACCTTAACGGGAGCAATGTGC

>TBP.ENCTRL.E3E4

GACTCCCATGACCCCCATCACTCCTGCCACGCCAGCTTCGGAGAGTTCTGGGATTGTACCGCAGCTGCAAAATATTGTATCCACAGTGAATCTTGGTTGTAAACTTGACCT

>ALK.5p.E14E15
TGCATTGGAGAGAACAATGTGATAGAAGAAGAAATCCGTGTGAACAGAAGCGTGCATGAGTGGGCAGGAGGCGGAGGAGGAGGGGGTGGAGCCACCTACGTATTT
AAGATGAAGGATGGAGTGCCGGTGCCCCTGATCATTGCAGCCGGAGGTGGTGGCAGGGCCTACGGGGCCAAGACAGACACGTTCCACCCAGAGA

>ALK.3p.E22E23
AAGTGGCTGTGAAGACGCTGCCTGAAGTGTGCTCTGAACAGGACGAACTGGATTTCCTCATGGAAGCCCTGATCATCAGCAAATTCAACCACCAGAACATTGTTCGCT
GCATTGGGGTGAGCCTGCAATCCCTGCCCCGGTTCATCCTGCTGGAGCTCATGGCGGGGGGAGACCTCAAGTCCTTCCTCCGAGAGACC

>NTRK1.E3E4.WT

TTCGTGGCGCCAGATGCCTTCCATTTCACTCCTCGGCTCAGTC

>NTRK1.E16E17.WT

GTGCCTGCCCACCAGAGGTCTACGCCATCATGCGG

>AR.E7E8.WT

CATCCTGCTCAAGACGCTTCTACCAGCTCACCAAGCTCCTGGACTCCGTGCAGCCTATTGCGAGAGAGCTGCATCAGTTCACTTTTGACCTGCTAATCAAGTCACACATGG

>BRCA1.E5E6.WT

CTACAGAAACCGTGCCAAAAGACTTCTACAGAGTGAACCCG

>MDM4.E3E4.WT

ACATAATGGTGAAGCAACTTTATGATCAGCAGGAGCAGC

>ALK-PTPN3.A11P3

CCAGGTTCTTCTGGATATGGTGCACAACCACCTGGGTGTGACTG

>ALK.Int19.ATI

CCAGACTAACATGACTCTGCCCTATATAATACAAATAATTATTTTCCATATATCTGATTTTTAGCTTTGCATTTACTTTAAATCATGCTTCAATTAAAGACACACCTTCTTTA
ATCATTTTATTAGTATTTCTAAGTATGATGGAAAGGTTCAGAGCTCAGGGGAGGATATGGAGATCCAGGGAGGCTTCCTGTAGGAAGTGGCCTGTGTAGTGCTTCAAG

>NTRK1.N6N7N8.WT

GCGGAGTGCCTGAACAGAAGCTGCAGTGTCATGGGCAAGGGCCCCTGGCCCACATGCCCAATGCCAGCTGTGGTGTGCCCACGCTGAAGGTCCAGGTGCCCAATGCC
TCGGTGGATGTGGGGGACGACGTGCTGCTGCGGTGCCAGGTGGAGGGGCGGGGCCTGGAGCAGGCCGGCTGGATCCTCACAGAGCTGGAGCAGTCAGCCACGGTG

>BRCA1.B19B20B21.WT

GGGTGACCCAGTCTATTAAAGAAAGAAAAATGCTGAATGAGCATGATTTTGAAGTCAGAGGAGATGTGGTCAATGGAAGAAACCACCAAGGTCCAAAGCGAGCAAG
AGAATCCCAGGACAGAAAGATCTTCAGGGGGCTAGAAATCTGTTGCTATGGGCCCTTCACCAACATGCCCACAGATCAACTGGAATGGATGGTACAGCTGTGTGGTGC

>BRCA1.B13B14B15.WT

GATTCGCATATACATGGCCAAAGGAACAACTCCATGTTTTCTAAAAGGCCTAGAGAACATATATCAGTATTAACTTCACAGAAAAGTAGTGAATACCCTATAAGCCAGA
ATCCAGAAGGCCTTTCTGCTGACAAGTTTGAGGTGTCTGCAGATAGTTCTACCAGTAAAAATAAAGAACCAGGAGTGGAAAGGTCATCCCCTTCTAAATGCCCATCATT

>BRCA1.B7B8B9.WT

GAACTGTGAGAACTCTGAGGACAAAGCAGCGGATACAACCTCAAAAGACGTCTGTCTACATTGAATTGGGATCTGATTCTTCTGAAGATACCGTTAATAAGGCAACTTA
TTGCAGTGTGGGAGATCAAGAATTGTTACAAATCACCCCTCAAGGAACCAGGGATGAAATCAGTTTGGATTCTGCAAAAAAGGCTGC

>BRCA1.B16B17B18.WT

AGCTTCAACAGAAAGGGTCAACAAAAGAATGTCCATGGTGGTGTCTGGCCTGACCCCAGAAGAATTTATGCTCGTGTACAAGTTTGCCAGAAAACACCACATCACTTTA
ACTAATCTAATTACTGAAGAGACTACTCATGTTGTTATGAAAACAGATGCTGAGTTTGTGTGTGAACGGACACTGAAATATTTTCTAGGAATTGCGGGAGGAAAATG

>MRPL13.ENCTRL.E1.E2

CCTGGCAAACTTGCTGCTATGGCATCTATAAGAC

Expression Controls:

MYC

chr8:128751265

HMBS

chr11:118960975

MRPL13

chr8:121455461

ITGB7

chr12:53585787

LRP1

chr12:57591167

TBP

chr6:170871321
