# Supplementary material for: Utilisation of semiconductor sequencing for detection of actionable fusions in solid tumours
Source: PLoS One. 2022 Aug 19;17(8):e0246778. doi: 10.1371/journal.pone.0246778 (PMC9390944; doi:10.1371/journal.pone.0246778)
Supplement: S5 Table — (PDF) [file pone.0246778.s007.pdf]

Supplementary Table 5. Quality control metrics.

Quality Metric	Controlled parameters
Chip loading	≥60%
Total reads	>45 million reads
Enrichment	100% ideal
Clonal: Polyclonal ratio	Up to 55% polyclonal
Low Quality	≤26% approximately.
Usable reads	≥30%
Aligned bases	≥80% Can be less if base coverage, and % reads on target is high
Unaligned bases	≤20%
Mean raw accuracy	This value should be as close to 100% as possible
Overall read length histogram	Median read length 98-115bp for DNA only, 65-90bp RNA only, DNA & RNA 100bp ±20

DNA

Quality Metric	Controlled parameters
Number of mapped reads	>4.5 million
Percent reads on target	>90%
Average base coverage depth	>1200
Uniformity of amplicon (base) coverage	>90% (degraded 85-90%)
Percent assigned amplicon reads	>90%
Amplicons with no strand bias	>90%
Amplicons reading end to end	>80%
% base reads on target	>85%
coverage at 1X	>90% coverage is required at 500X for samples with tumour percentages below 40%
coverage at 20X	
coverage at 100X	
coverage at 500X	
MAPD value	In the presence of a copy number variant, this value should be <0.5

RNA

Quality Metric	Controlled parameters
Mapped reads	>40,000 for software to call a fusion, (should be over 500,000)
Expression Control Genes MYC	>15 read counts indicates the gene is present (5 out of 6 genes should be present to accept the presence of a fusion)
Expression Control Genes HMBS	
Expression Control Genes TBP	
Expression Control Genes LRP1	
Expression Control Genes ITGB7	
Expression Control Genes MRPL13	
