# Supplementary material for: Utilisation of semiconductor sequencing for detection of actionable fusions in solid tumours
Source: PLoS One. 2022 Aug 19;17(8):e0246778. doi: 10.1371/journal.pone.0246778 (PMC9390944; doi:10.1371/journal.pone.0246778)
Supplement: S6 Table — (PDF) [file pone.0246778.s008.pdf]

Supplementary Table 6. Detected actionable gene fusions in the sample cohort (n=1112).

Sample No.	Primary site	Histological sub-type	Primary gene	HGVS	Tumour content	Read count
1	Lung	NSCLC	RET	KIF5B(16)-RET(12)	30%	252543
2	Glioblastoma/Glioma	Glioblastoma	MET	CAPZA2(4)-MET(2)	85%	8004
3	Glioblastoma/Glioma	Glioblastoma	PDGFRA	FIP1L1(13)-PDGFRA(12)	85%	101
4	Head and neck	Tongue, squamous cell carcinoma	PIK3CA	TBL1XR1(1)-PIK3CA(2)	70%	454
5	Head and Neck	Right cheek,Squamous cell carcinoma	FGFR3	FGFR3(17)-TACC3(11)	90%	83057
6	Head and Neck	Tongue, squamous cell carcinoma	PIK3CA	TBL1XR1(1)-PIK3CA(2)	85%	65
7	Glioblastoma/Glioma	Glioblastoma	EGFR	EGFR(1)-EGFR(8)	80%	26217
8	Kidney	Renal Cell Carcinoma (clear cell)	MET	MET(13)-MET(15)	60%	1057
9	Prostate	Adenocarcinoma	FGFR3	FGFR3(17)-TACC3(10)	80%	40666
10	Glioblastoma/Glioma	Glioblastoma	FGFR3	FGFR3(17)-TACC3(6)	70%	115731
11	Breast	Invasive ductal carcinoma	PIK3CA	TBL1XR1(1)-PIK3CA(2)	80%	48
12	Colorectal	Colon adenocarcinoma	PIK3CA	TBL1XR1(1)-PIK3CA(2)	40%	56
13	Lung	NSCLC	MET	MET(13)-MET(15)	35%	150806
14	Colorectal	Right colon adenocarcinoma	RSPO2	EIF3E(1)-RSPO2(2)	80%	972
15	Kidney	Renal Cell Carcinoma (clear cell)	FGFR1	WHSC1L1(1)-FGFR1(2)	80%	67
16	Small bowel	Metastatic adenocarcinoma of duodenum in a lymph node	FGFR1	WHSC1L1(1)-FGFR1(2)	95%	269
17	Breast	Invasive ductal carcinoma	FGFR1	WHSC1L1(1)-FGFR1(2)	60%	804
18	Breast	Metastatic carcinoma to the liver	FGFR1	WHSC1L1(1)-FGFR1(2)	80%	106
19	Pancreatic	Metastatic pancreatic adenocarcinoma to the peritoneum	PIK3CA	FNDC3B(3)-PIK3CA(2)	30%	58
20	Pancreatic	Metastatic pancreatic adenocarcinoma to the peritoneum	PIK3CA	TBL1XR1(1)-PIK3CA(2)	30%	174
21	Glioblastoma/Glioma	Glioblastoma	EGFR	EGFR(24)-SEPT14(10)	95%	25476
22	Lung	NSCLC	MET	SND1(10)-MET(2)	30%	1236
23	Liver	Cholangiocarcinoma	FGFR2	FGFR2(17)-BICC1(3)	75%	211834
24	Colorectal	Rectum adenocarcinoma	MET	TMEM178B(2)-MET(2)	80%	2455
25	CUP	Poorly differentiated adenocarcinoma in the liver	PIK3CA	TBL1XR1(1)-PIK3CA(2)	85%	116
26	Sarcoma	Angiosarcoma	BRAF	PCM1(29)-BRAF(4)	70%	1495
27	Endometrial	Metastatic Endometrium adenocarcinoma in the lung	PIK3CA	TBL1XR1(1)-PIK3CA(2)	35%	67
28	Lung	NSCLC	PIK3CA	TBL1XR1(1)-PIK3CA(2)	70%	236
29	Breast	Invasive ductal carcinoma	FGFR1	WHSC1L1(1)-FGFR1(2)	75%	434
30	Pancreatic	Adenocarcinoma	PIK3CA	TBL1XR1(1)-PIK3CA(2)	90%	343
31	Sarcoma	Metastatic osteosarcoma to the lung	FGFR1	WHSC1L1(1)-FGFR1(2)	90%	202
32	Cervical	Adenocarcinoma	PIK3CA	TBL1XR1(1)-PIK3CA(2)	30%	376
33	Lung	NSCLC	PIK3CA	TBL1XR1(1)-PIK3CA(2)	85%	174
34	Lung	NSCLC	RET	NCOA4(7)-RET(12)	85%	11721
35	Glioblastoma/Glioma	Glioblastoma	MET	CAPZA2(1)-MET(6)	95%	97
36	Glioblastoma/Glioma	Glioblastoma	MET	MET(13)-MET(15)	95%	1450
37	Other	Vulva, pecoma	PIK3CA	TBL1XR1(1)-PIK3CA(2)	70%	248
38	Colorectal	Rectum adenocarcinoma	MET	MET(13)-MET(15)	55%	1024
39	Upper GI	Metastatic GOJ adenocarcinoma to the liver	PIK3CA	TBL1XR1(1)-PIK3CA(2)	80%	231
40	Prostate	Adenocarcinoma	BRAF	SND1(10)-BRAF(9)	90%	187
41	Glioblastoma/Glioma	Anaplastic astrocytoma	EGFR	EGFR(1)-EGFR(8)	70%	84070
42	Other	Squamous Cell Carcinoma right buttock	MET	MET(13)-MET(15)	90%	4686
43	Pancreatic	Adenocarcinoma	MET	MET(13)-MET(15)	30%	1072
44	Glioblastoma/Glioma	Glioblastoma	EGFR	EGFR(24)-SEPT14(10)	95%	228049
45	CUP	Metastatic adenocarcinoma in liver (CUP)	FGFR1	WHSC1L1(1)-FGFR1(2)	95%	1229
46	Glioblastoma/Glioma	Anaplastic oligodendroglioma	EGFR	EGFR(24)-SEPT14(10)	95%	157146
47	Colorectal	Metastatic anal squamous cell carcinoma to the liver	EGFR	EGFR(24)-SEPT14(10)	65%	48
48	CUP	Metastatic adenocarcinoma in the liver	MET	MET(13)-MET(15)	70%	2928
49	Glioblastoma/Glioma	Glioblastoma	EGFR	EGFR(1)-EGFR(8)	90%	1372
50	Glioblastoma/Glioma	Glioblastoma	MET	PTPRZ1(1)-MET(2)	90%	312
51	Glioblastoma/Glioma	Glioblastoma	FGFR3-TACC3	FGFR3(17)-TACC3(6)	40%	279210
52	Colorectal	Colon Metastatic Adenocarcinoma to the liver	PIK3CA	TBL1XR1(1)-PIK3CA(2)	80%	111
53	Glioblastoma/Glioma	Glioblastoma	MET	PTPRZ1(1)-MET(2)	40%	36161
54	Glioblastoma/Glioma	Glioblastoma	PIK3CA	TBL1XR1(1)-PIK3CA(2)	40%	77
55	Liver	Cholangiocarcinoma	FGFR2	FGFR2(17)-BICC1(3)	90%	837995
56	Glioblastoma/Glioma	Glioblastoma	AGK-BRAF	AGK(2)-BRAF(8)	80%	310
57	Pancreatic	Adenocarcinoma	PIK3CA	TBL1XR1(1)-PIK3CA(2)	60%	11616
58	Bladder	Adenocarcinoma	MET	MET(13)-MET(15)	80%	1869
59	Colorectal	Rectum adenocarcinoma	PIK3CA	TBL1XR1(1)-PIK3CA(2)	30%	116
60	Colorectal	Metastatic colon carcinoma to the liver	MET	CAPZA2(4)-MET(2)	90%	257
61	Colorectal	Metastatic colon carcinoma to the liver	MET	MET(13)-MET(15)	90%	3358
62	Upper GI	Squamous Cell Carcinoma	PIK3CA	TBL1XR1(1)-PIK3CA(2)	95%	268
63	Other	Teratoma	BRAF	TMEM178B(2)-BRAF(9)	90%	1662
64	Breast	Metastatic Adenocarcinoma to an axillary lymph node	FGFR1	WHSC1L1(1)-FGFR1(2)	90%	132
65	Breast	Metastatic Carcinoma to pleura	NTRK3	KANK1(2)-NTRK3(14)	90%	1152
66	Upper GI	Adenocarcinoma	FGFR1	WHSC1L1(1)-FGFR1(2)	90%	315
67	Prostate	Adenocarcinoma	BRAF	BRAF(1)-MRPS33(2)	30%	124
68	Upper GI	Adenocarcinoma	PIK3CA	TBL1XR1(1)-PIK3CA(2)	60%	167
69	Glioblastoma/Glioma	Ephitelial Glioblastoma	MET	MET(13)-MET(15)	90%	1503
70	Ovarian	Ovarian Carcinoma	PIK3CA	TBL1XR1(1)-PIK3CA(2)	90%	395
71	Breast	Metastatic Carcinoma to the liver	FGFR1	FGFR1(1)-NRG1(2)	90%	1462
72	Prostate	Adenocarcinoma	EGFR	EGFR(1)-EGFR(8)	30%	3476
73	Ovarian	Endometrioid Adenocarcinoma With Yolk Sac Differentiation	FGFR1	WHSC1L1(1)-FGFR1(2)	90%	523
74	Glioblastoma/Glioma	Glioblastoma	EGFR	EGFR(1)-EGFR(8)	90%	10784
75	Glioblastoma/Glioma	Glioblastoma	EGFR	EGFR(1)-EGFR(8)	90%	17111
76	Sarcoma	Leiomyosarcoma abdomen	FGFR1	WHSC1L1(1)-FGFR1(2)	90%	162
77	Thyroid	Anaplastic Thyroid Carcinoma	PIK3CA	TBL1XR1(1)-PIK3CA(2)	65%	80
78	Endometrial	Metastatic Adenocarcinoma to a lymph node	PIK3CA	TBL1XR1(1)-PIK3CA(2)	90%	158
79	Glioblastoma/Glioma	High Grade Glioma	FGFR3	FGFR3(18)-TACC3(10)	60%	33232
80	Glioblastoma/Glioma	Glioblastoma	MET	MET(13)-MET(15)	90%	1182
81	Colorectal	Adenocarcinoma	RSPO3	PTPRK(1)-RSPO3(2)	90%	41
82	Thyroid	Anaplastic Carcinoma	PIK3CA	TBL1XR1(1)-PIK3CA(2)	80%	
83	Head and Neck	Squamous cell carcinoma in left lateral cervical subcutaneous tissue	PIK3CA	TBL1XR1(1)-PIK3CA(2)	60%	74

84	Thyroid	Poorly Differentiated Carcinoma	RET	CCDC6(1)-RET(12)	60%	55557
85	Glioblastoma/Glioma	Glioblastoma	PIK3CA	TBL1XR1(1)-PIK3CA(2)	70%	133
86	Ovarian	High Grade Serous Carcinoma in omentum	PIK3CA	TBL1XR1(1)-PIK3CA(2)	70%	343
87	Glioblastoma/Glioma	Glioblastoma	EGFR	EGFR(1)-EGFR(8)	90%	5643
88	Pancreatic	Adenocarcinoma metastatic to the liver	PIK3CA	TBL1XR1(1)-PIK3CA(2)	50%	90
89	Lung	Metastatic Adenocarcinoma to a lymph node	RET	CCDC6(1)-RET(12)	80%	43120

Highlighted rows indicate two gene fusions detected in the same sample.
