# Supplementary material for: Utilisation of semiconductor sequencing for detection of actionable fusions in solid tumours
Source: PLoS One. 2022 Aug 19;17(8):e0246778. doi: 10.1371/journal.pone.0246778 (PMC9390944; doi:10.1371/journal.pone.0246778)
Supplement: S7 Table — (PDF) [file pone.0246778.s009.pdf]

Supplementary Table 7. Low frequency gene fusions detected in the sample cohort.

Fusion	Cancer Type (n=)	Total n= 40
**FGFR3-TACC3**	Head & Neck, squamous cell carcinoma (1)<br>Prostate, adenocarcinoma (1)<br>Glioblastoma (2)<br>High grade glioma (1)	5
**EGFR VIII**	Glioblastoma (6)<br>Prostate (1)	7
**EGFR-SEPT14**	Glioblastoma (2)<br>Anaplastic oligodendroglioma (1)<br>Colorectal squamous cell carcinoma (1)	4
**CAPZA2- MET**	Glioblastoma (2)<br>Colorectal adenocarcinoma (1)	3
**FGFR2-BICC1**	Cholangiocarcinoma (2)	2
**PTPRZ1-MET**	Glioblastoma (2)	2
**CCDC6-RET**	Thyroid carcinoma (1)<br>Lung adenocarcinoma (1)	2
**AGK-BRAF**	Glioblastoma	1
**EIF3E-RSPO2**	Colorectal adenocarcinoma	1
**FIP1L1-PDGFRA**	Glioblastoma	1
**FNDC3B-PIK3CA**	Pancreatic adenocarcinoma	1
**KIF5B-RET**	NSCLC	1
**PCM1-BRAF**	Angiosarcoma	1
**NCOA4-RET**	NSCLC	1
**SND1-BRAF**	Prostate, adenocarcinoma	1
**SND1-MET**	NSCLC	1
**TMEM178B-MET**	Rectal adenocarcinoma	1
**TMEM178B-BRAF**	Teratoma	1
**BRAF-MRPS33**	Prostate, adenocarcinoma	1
**FGFR1-NRG1**	Breast adenocarcinoma	1
**KANK1-NTRK3**	Breast adenocarcinoma	1
**PTPRK-RSPO3**	Colorectal adenocarcinoma	1
