# Supplementary material for: Utilisation of semiconductor sequencing for detection of actionable fusions in solid tumours
Source: PLoS One. 2022 Aug 19;17(8):e0246778. doi: 10.1371/journal.pone.0246778 (PMC9390944; doi:10.1371/journal.pone.0246778)
Supplement: S8 Table — (PDF) [file pone.0246778.s010.pdf]

Supplementary Table 8. Frequency of actionable gene fusion and cancer type.

PRIMARY SITE	Frequency of detected fusions in each tumour type	Fusion gene detected	Tumour samples n= 1112
**Thyroid**	33.33%	TBL1XR1-PIK3CA (2); CCDC6-RET (1)	9
**Glioblastoma**	23.46%	EGFR VIII (6); FGFR3-TACC3 (3); MET-MET (3); EGFR-SEPT14 (3); CAPZA2-MET (2); PTPRZ1-MET (2); TBL1XR1-PIK3CA (2); FIP1L1-PDGFRA (1); AGK-BRAF (1)	81
**Small bowel**	14.29%	WHSC1L1-FGFR1 (1)	7
**Other**	13.04%	TMEM178B-BRAF (1); MET-MET (1); TBL1XR1-PIK3CA (1)	23
**Head and neck**	12.12%	TBL1XR1-PIK3CA (3); FGFR3-TACC3 (1)	33
**Kidney**	11.11%	WHSC1L1-FGFR1 (1); MET-MET (1)	18
**Prostate**	9.52%	FGFR3-TACC3 (1); SND1-BRAF (1); BRAF-MRPS33 (1); EGFR VIII (1)	42
**Endometrial**	8.70%	TBL1XR1-PIK3CA (2)	23
**Lung**	8.11%	TBL1XR1-PIK3CA (2); NCOA4-RET (1); SND1-MET (1); KIF5B-RET (1); CCDC6-RET (1); MET-MET (1)	74
**CUP**	7.89%	TBL1XR1-PIK3CA (1); WHSC1L1-FGFR1 (1); MET-MET (1)	38
**Pancreatic**	7.14%	TBL1XR1-PIK3CA (4); FNDC3B-PIK3CA (1); MET-MET (1)	70
**Liver**	6.25%	FGFR2-BICC1 (2)	32
**Upper GI**	5.33%	TBL1XR1-PIK3CA (3); WHSC1L1-FGFR1 (1)	75
**Sarcoma**	5.26%	WHSC1L1-FGFR1 (2); PCM1-BRAF (1)	57
**Colorectal**	5.17%	TBL1XR1-PIK3CA (3); MET-MET (2); EGFR-SEPT14 (1); EIF3E-RSPO2 (1); TMEM178B-MET (1); CAPZA2-MET (1); PTPRK-RSPO3 (1)	174
**Cervical**	4.55%	TBL1XR1-PIK3CA (1)	22
**Bladder**	4.17%	MET-MET (1)	24
**Breast**	3.98%	WHSC1L1-FGFR1 (4); TBL1XR1-PIK3CA (1); FGFR1-NRG1 (1); KANK1-NTRK3 (1)	176
**Ovarian**	3.57%	TBL1XR1-PIK3CA (2); WHSC1L1-FGFR1 (1)	84
