# Supplementary material for: Utilisation of semiconductor sequencing for detection of actionable fusions in solid tumours
Source: PLoS One. 2022 Aug 19;17(8):e0246778. doi: 10.1371/journal.pone.0246778 (PMC9390944; doi:10.1371/journal.pone.0246778)
Supplement: S9 Table — (PDF) [file pone.0246778.s011.pdf]

Supplementary Table 9. Detected driver genes and fusions by gender and age.

Driver Gene	Fusion	Male	Female	Median Age	
**PIK3CA**	TBL1XR1-PIK3CA	11	16	57	3
	FNDC3B-PIK3CA	0	1	86	1
	Total	*11*	*17*	*58*	
**MET**	MET-MET	5	6	61	
	CAPZA2-MET	2	1	62	3
	PTPRZ1-MET	2	0	53	2
	SND1-MET	0	1	48	
	TMEM178B-MET	0	1	46	
	Total	*9*	*9*	*61*	
**FGFR1**	WHSC1L1-FGFR1	0	11	52	4
	FGFR1-NRG1	0	1	61	
	Total	*0*	*12*	*53.5*	
**EGFR**	EGFR VIII	4	3	57	
	EGFR-SEPT14	2	2	56	7
	Total	*6*	*5*	*57*	
**FGFR3**	FGFR3-TACC3	5	0	62	36
**RET**	CCDC6-RET	0	2	55	12
	KIF5B-RET	1	0	63	
	RET-NCOA4	1	0	75	1
	Total	*2*	*2*	*64*	
**BRAF**	AGK-BRAF	1	0	45	2
	BRAF-MRPS33	1	0	52	2
	PCM1-BRAF	0	1	74	
	SND1-BRAF	1	0	61	3
	TMEM178B-BRAF	1	0	9	
	Total	*4*	*1*	*52*	
**FGFR2**	FGFR2-BICC1	0	2	42	3
**NTRK3**	KANK1-NTRK3	0	1	52	
**PDGFRA**	FIP1L1-PDGFRA	1	0	68	
**RSPO2**	EIF3E-RSPO2	1	0	58	
**RSPO3**	PTPRK-RSPO3	1	0	60	8
**Grand Total**		**40**	**49**	**58**	
