# Supplementary material for: Utilisation of semiconductor sequencing for detection of actionable fusions in solid tumours
Source: PLoS One. 2022 Aug 19;17(8):e0246778. doi: 10.1371/journal.pone.0246778 (PMC9390944; doi:10.1371/journal.pone.0246778)
Supplement: Attachment — Submitted filename: Reviewer Comments_Fusions.docx [file pone.0246778.s012.docx]

4/3/2021

Dear Dr., Reddi,

Thank you for inviting me to review the original research article titled “**Utilisation of semiconductor sequencing for detection of actionable fusions in solid tumors”** by Loddo et al.

This is a well-written manuscript emphasizing the importance of fusion gene analysis and precision medicine in the NGS era. Especially, the discussion section of the manuscript covered and linked an array of topics. I do not see any major flaws in the manuscript, and I recommend that this manuscript can be accepted for publication once addressing the following suggestions.

**Comments**

1) Line 34 has a typo with an extra “W” before the word “Where”.

2) In Line 65, the sentence starting with “This platform enables..” can be rephrased as “This platform enables detection of 867 druggable driver-partner oncogenic fusions via analysis of 51 driver and 349 partner genes, with linkage to 140 targeted 68 therapy protocols”.

3) Line 96 can be rephrased as “ The NGS platform includes the targeting of 51 driver genes and 349 partner genes enabling detection of 867 druggable driver-partner oncogenic fusions that is linked to 140 targeted therapy protocols.”

4) In Lines 137 and 141 – I would not call EGFRvIII as a gene fusion event, but it is a gene rearrangement.

5) In Line 228 – there is a typo “ keeping wFith”

6) Line 231 – The first sentence can be rephrased.

Thanks,

Pavalan
